# Human Milk Oligosaccharides in Exercise Nutrition: Structure-Specific Evidence, Human Efficacy, and Translational Bottlenecks

**DOI:** 10.3390/nu18203417

**Published:** 2026-10-18

**Authors:** Chuyuan Qiao, Sitong Guo, Shenglei Yang, Chuwei Yang, Yan Wang

**Affiliations:** 1 School of Sports Medicine and Rehabilitation, Beijing Sport University, Beijing 100084, China; xiaosadashuai@163.com (C.Q.); y17616251939@163.com (S.Y.); chuizigou@163.com (C.Y.); 2 National Institute of Biological Sciences, Beijing 102206, China; guositong@nibs.ac.cn

**Keywords:** human milk oligosaccharides, exercise nutrition, skeletal muscle, gut microbiota, mitochondrial metabolism, training adaptation, translational evidence

## Abstract

**Background/Objectives:** Human milk oligosaccharides (HMOs) are structurally diverse bioactive carbohydrates being investigated beyond early-life nutrition. General gut–muscle and short-chain fatty acid (SCFA) frameworks are well described. This review aimed to assess structure-specific evidence for HMO effects on human exercise outcomes and identify the main translational bottlenecks. **Methods:** This critical translational review systematically searched PubMed/MEDLINE (Medical Literature Analysis and Retrieval System Online), Embase, Web of Science Core Collection, Scopus, and SPORTDiscus. Evidence was assigned non-exclusively to Layer A (direct exercise evidence), Layer B (exercise-relevant mechanisms), or Layer C (adult translational evidence). We separately evaluated HMO structure, prespecified human efficacy outcomes, within-model causal support, and distance from healthy human exercise. **Results:** The five databases yielded 4787 records; 40 reports representing 38 independent studies were included. Several HMOs were tolerated at the doses and durations tested and produced structure-, dose-, and host-dependent microbial responses; limited systemic exposure was documented for selected structures. Some intestinal and disease-model pathways had perturbation, knockout, transfer, or rescue support. However, direct human exercise evidence was sparse and inconsistent: primary or overall interaction tests were generally null, whereas favorable findings were mainly secondary, interim, within-group, or exploratory. No study established the complete sequence from oral exposure to target engagement, healthy skeletal-muscle adaptation, and improved performance. **Conclusions:** Current evidence supports biological activity and testability of selected HMOs, but not an HMO class effect, a confirmed ergogenic mechanism, or a recommendation for exercise-performance use. Translation is constrained by four unresolved links: structure to exposure, exposure to target engagement, target engagement to muscle phenotype, and muscle phenotype to functional benefit.

## 1. Introduction

Human milk oligosaccharides (HMOs) are structurally diverse unconjugated carbohydrates whose concentrations and profiles vary with maternal secretor and Lewis status, lactation stage, and geographical population [1,2,3,4,5,6,7]. Research has focused on early-life nutrition, where HMOs influence the infant microbiota, mucosal barrier, and immune development [7,8,9,10]. These findings inform adult research but do not establish adult efficacy. Scalable production now enables studies of tolerability, microbiome effects, and systemic availability. Trials indicate that 2′-fucosyllactose (2′-FL), lacto-N-neotetraose (LNnT), and 6′-sialyllactose (6′-SL) are generally tolerated and can modify microbiome features in some adults [11,12,13]. Orally administered 2′-FL has also been detected in plasma and urine, supporting limited systemic availability [13]. Microbial and metabolic responses can vary with HMO structure and dose, age-related or baseline microbiota, and metabolic phenotype, limiting class-wide inference [11,13,14,15].

At the molecular level, HMOs comprise glucose, galactose, N-acetylglucosamine, fucose, and N-acetylneuraminic acid arranged around a reducing-end lactose core. Differences in chain extension, branching, and glycosidic linkage generate distinct structures and positional isomers. They are commonly grouped as neutral fucosylated, neutral non-fucosylated, and acidic sialylated oligosaccharides: 2′-FL, lacto-N-tetraose (LNT)/LNnT, and 3′-sialyllactose (3′-SL)/6′-SL illustrate these categories, respectively [16]. LNT and LNnT differ in the linkage of terminal galactose, whereas 3′-SL and 6′-SL differ in the position of sialic-acid attachment. HMOs largely resist host digestive enzymes and can serve as substrates for selected gut microbes; their biological functions also include interactions with epithelial and immune systems [17]. These structural distinctions provide a biochemical basis for evaluating individual HMOs separately, but do not establish equivalent biological responses or exercise benefits across the class.

In the mammary gland, glycosyltransferases extend and modify the lactose core to generate diverse HMOs. Maternal secretor status reflects the activity of α1,2-fucosyltransferase 2 (FUT2), which enables synthesis of 2′-FL and related α1,2-fucosylated HMOs. Non-secretor milk generally contains markedly lower or undetectable amounts of these structures, rather than lacking HMOs altogether. α1,3/4-Fucosyltransferase 3 (FUT3), associated with Lewis status, further influences fucosylated HMO profiles [18]. Lactation stage, gestational age, and maternal characteristics, including diet, have also been associated with HMO composition, although the evidence and underlying mechanisms vary across factors [19]. These determinants describe natural milk composition; they should not be conflated with the defined single structures or mixtures administered in adult studies, nor interpreted as evidence that maternal secretor status predicts adult supplementation efficacy.

Microbial fermentation of HMOs can contribute to the production of short-chain fatty acids (SCFAs), principally acetate, propionate, and butyrate. SCFAs act through both non-epigenetic and epigenetic mechanisms. They provide metabolic substrates, including fuel for colonocytes, and activate cell-surface receptors such as free fatty acid receptor 2 (FFAR2; also known as G-protein-coupled receptor 43, GPR43) and free fatty acid receptor 3 (FFAR3; also known as G-protein-coupled receptor 41, GPR41), influencing epithelial and immune signaling. Intracellularly, butyrate and propionate can inhibit histone deacetylases (HDACs), modifying histone acetylation and gene expression in a cell- and exposure-dependent manner [20]. Receptor-mediated signaling and intracellular effects can interact, rather than forming mutually exclusive pathways. These general SCFA mechanisms do not establish that HMO supplementation produces sufficient metabolite exposure to mediate healthy human muscle adaptation or improved exercise performance.

HMO biology intersects with exercise nutrition because performance and adaptation depend on substrate supply, barrier integrity, redox and inflammatory regulation, mitochondrial function, protein turnover, and recovery [21,22,23,24]. Exercise also modifies gastrointestinal permeability and the gut microbiome in a context-dependent manner [25,26,27,28]. Across disparate models, selected HMOs alter microbial output, support intestinal-barrier signaling, or reach systemic and muscle-related targets [28,29,30,31]. Exercise and muscle-atrophy models also report changes in myosin heavy chain (MHC), oxidative phosphorylation (OXPHOS), AMP-activated protein kinase (AMPK), phosphoinositide 3-kinase (PI3K)/protein kinase B (AKT), and protein-degradation pathways [32,33,34,35]. These findings support biological plausibility, but model and exposure differences preclude a definitive human mechanism. Sports-nutrition recommendations require prespecified efficacy and safety evidence in defined populations [36].

Direct evidence remains limited and heterogeneous. In the resistance-training study, 6′-SL did not improve prespecified primary outcomes; favorable interim or secondary signals were inconsistent [37]. A 2′-FL lifestyle intervention likewise showed no between-group benefit for primary body-composition or aerobic outcomes, and favorable patterns were mainly exploratory [38]. Animal and nematode models reported more favorable endurance, fatigue, and muscle phenotypes, but remain translationally distant [32,33,34,35,39]. Exercise may also regulate HMOs upstream: a maternal exercise–milk 3′-SL pathway was supported in mice, whereas acute exercise did not alter milk 3′-SL or 6′-SL in humans [40,41]. These directions and time scales should be interpreted separately.

The central question is whether a defined HMO, delivered at a characterized dose, achieves sufficient exposure and target engagement to modify healthy human muscle and a prespecified exercise outcome. Adult studies establish feasibility and microbial responsiveness, model systems provide local mechanistic support, and small exercise studies test selected outcomes. These components do not yet form a human causal chain with demonstrated temporal order, mediator necessity, and reproducible efficacy.

The primary objective was to assess whether structure-specific HMO evidence supports human exercise benefits and to identify the main translational bottlenecks. The three-layer framework organized direct exercise evidence (A), exercise-relevant mechanistic evidence (B), and adult translational evidence (C) without treating them as interchangeable.

**Figure 1:**
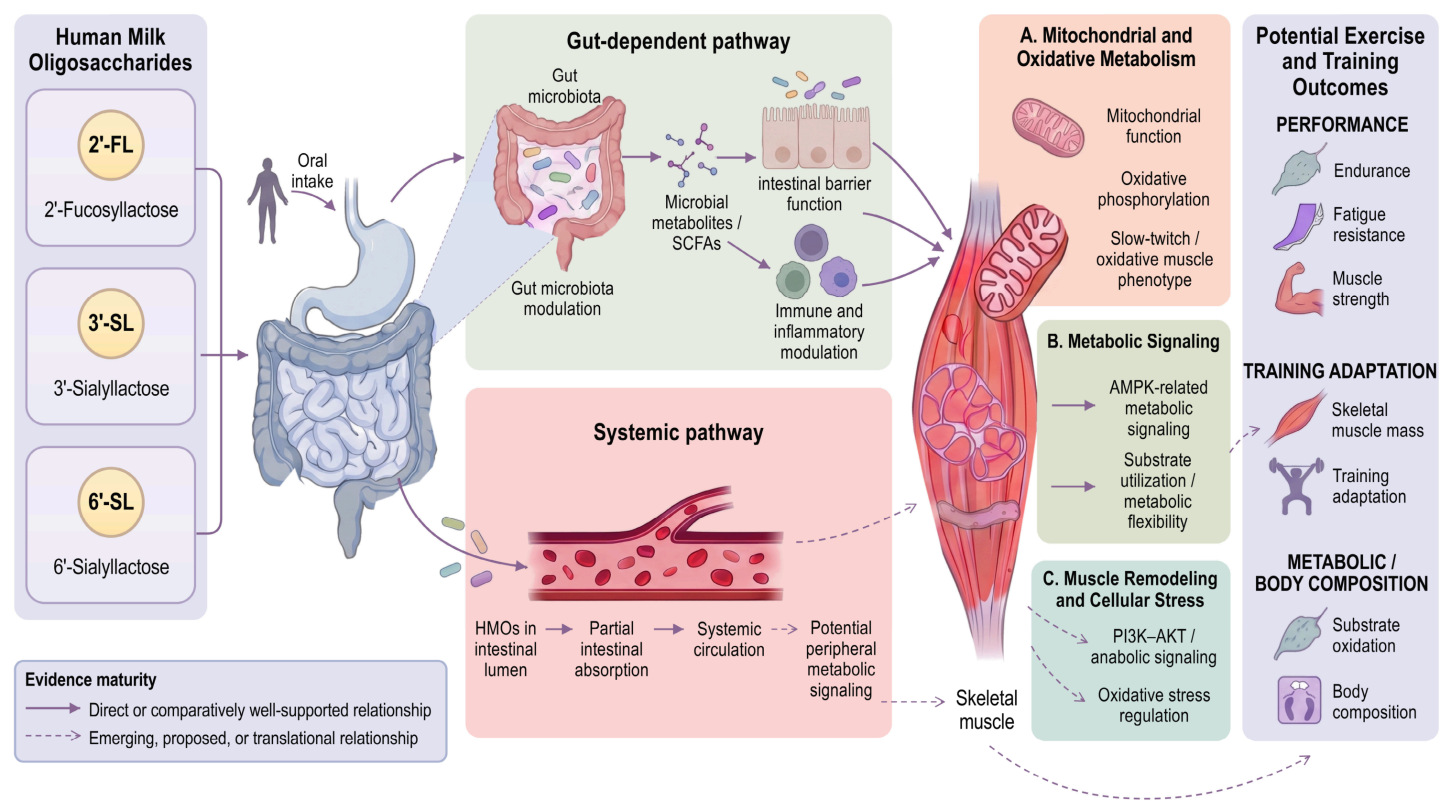
Evidence-organizing framework for structure-specific HMO pathways relevant to exercise adaptation. The gut-dependent and systemic routes provide a scaffold for locating evidence, not a claim that the general gut–muscle axis is novel or that the full pathway has been validated. Solid arrows indicate relationships directly measured and supported by functional perturbation, transfer, inhibition, genetic deletion, or rescue within the cited experimental system; dashed arrows indicate association, cross-model extrapolation, or an unclosed translational connection. No complete causal chain has yet linked a defined oral HMO exposure to target engagement, healthy human skeletal-muscle adaptation, and improved exercise performance. HMO, human milk oligosaccharide. 2′-FL, 2′-fucosyllactose; 3′-SL, 3′-sialyllactose; 6′-SL, 6′-sialyllactose; SCFAs, short-chain fatty acids; AMPK, AMP-activated protein kinase; PI3K, phosphoinositide 3-kinase; AKT, protein kinase B.

Figure 1 organizes evidence into gut-dependent and systemic pathways. Solid lines indicate within-model relationships supported by direct measurement or perturbation, whereas dashed lines mark unverified links to healthy muscle and performance. The framework maps translational gaps without presuming exercise efficacy.

## 2. Materials and Methods

### 2.1. Review Design and Registration

This critical translational review used a structured and reproducible literature-identification process. Eligible evidence included human and animal studies, direct exercise outcomes, mechanistic experiments, and adult translational studies. HMO structure, exposure protocol, population, model, exercise paradigm, comparator, and outcome definition varied substantially across studies. A single pooled effect was therefore neither planned nor considered scientifically appropriate. The review protocol and methodological decisions were registered with the Open Science Framework (OSF; https://doi.org/10.17605/OSF.IO/YVTS8).

### 2.2. Information Sources and Literature Identification

We searched PubMed/MEDLINE (Medical Literature Analysis and Retrieval System Online), Embase, Web of Science Core Collection, Scopus, and SPORTDiscus. Representative search terms included “human milk oligosaccharide*”, fucosyllactos*, sialyllactos*, “lacto-N-neotetraose”, LNnT, “lacto-N-tetraose”, and difucosyllactos* in the HMO block; exercis*, train*, endurance, fatigue, “skeletal muscle*”, and “body composition” in the exercise block; mitochondri*, “oxidative phosphorylation”, “substrate oxidation”, and “oxidative stress” in the mechanistic block; and adult*, older, or elderly combined with microbiom*, microbiota, SCFA*, “intestinal barrier”, “systemic availability”, absorp*, or pharmacokinetic* in the adult-translational block. Synonyms were combined using OR, and the HMO block was joined to the evidence blocks using AND; syntax and controlled vocabulary were adapted to each database. Database-specific strategies, interfaces, dates, and export settings are reported in the Supplementary Methods, with complete search strings in Supplementary File S1.

Backward and forward citation tracking was conducted for included reports and prespecified seed studies. Trial registrations were managed separately from bibliographic records. The 25 records retrieved from Embase Clinical Trials were used only to identify ongoing or unpublished translational studies and were not merged into the bibliographic screening dataset.

### 2.3. Eligibility Framework and Study Selection

Studies were eligible if they informed at least one non-mutually exclusive evidence layer: direct evidence on HMOs and exercise, performance, skeletal muscle, fatigue, or body composition (Layer A); exercise-relevant HMO mechanisms (Layer B); or adult evidence on safety, microbiota, metabolites, barrier-related outcomes, systemic exposure, or pharmacokinetics (Layer C). A study could contribute to more than one layer. We excluded duplicate records, ineligible publication types, studies outside these domains, reports with insufficient information for eligibility assessment, and reports for which full text remained unavailable after documented retrieval attempts. Unretrieved reports and their access status are listed in Supplementary Table S1. Reports excluded after full-text assessment and their single primary exclusion reason are listed in Supplementary Table S2. Because this review integrated human intervention studies with observational, animal, cellular, organoid, ex vivo fermentation, and pharmacokinetic evidence, eligibility could not be adequately represented by a single conventional population, intervention, comparator, outcomes, and study design (PICOS) framework. We therefore used the prespecified A/B/C layered framework to classify direct exercise evidence, exercise-relevant mechanistic evidence, and adult translational evidence; PICOS elements were applied where relevant to human intervention studies.

Two reviewers independently reassessed records retained after title and abstract screening and manually confirmed all full-text eligibility decisions. Disagreements were resolved by discussion. Study selection is reported in the Results and the Preferred Reporting Items for Systematic Reviews and Meta-Analyses (PRISMA) 2020 flow diagram [42]. Before study-level synthesis, reports arising from the same cohort or experimental protocol were linked as study families. Complementary reports retained their non-overlapping outcomes but contributed only once to the count of independent studies, preventing double-counting of participants and inflation of the evidence base. Supplementary Table S3 maps the 40 included reports to 38 independent studies.

### 2.4. Data Extraction and Evidence Synthesis

At the report level, we extracted the study identifier; population or model; HMO structure or composition; route, dose, timing, and duration of exposure; comparator; exercise or training context; outcome definitions and assessment times; reported numerical results and uncertainty; author-defined primary and secondary outcomes; and study limitations. For reports within the same study family, non-overlapping outcomes were retained, whereas participants were counted once at the independent-study level. Supplementary Table S4 presents the evidence layer, design, direction of evidence, translational distance, and main boundary for each included report.

We used a structured narrative synthesis consistent with guidance for synthesis without meta-analysis [43]. Evidence was first organized by the A/B/C framework and then grouped by population or species, HMO structure and exposure, exercise context, outcome domain, and study design. Within each original outcome domain, findings were coded as favorable, null, unfavorable, mixed or heterogeneous, or unclear or underreported. These codes supported descriptive evidence mapping only; they were neither quantitative scores nor vote counts. Interpretation considered study design, precision, risk of bias, biological plausibility, and translational distance. Effect estimates were not pooled.

### 2.5. Risk of Bias and Study Quality Appraisal

Risk of bias was appraised with tools matched to study design and was not converted into a cross-design numerical score. Randomized human outcomes were assessed with Risk of Bias 2 (RoB 2), using the crossover version where applicable [44]. Of 10 key outcomes from randomized parallel or crossover trials, 4 were judged at low risk of bias and 6 raised some concerns; none were judged at high risk. Eligible non-randomized interventions were assessed with Risk of Bias in Non-randomized Studies of Interventions (ROBINS-I). Two were judged at serious risk of bias for causal effect estimation but retained for descriptive evidence on dose, microbiome response, tolerability, or feasibility [45]. The identified longitudinal cohort was assessed with the Joanna Briggs Institute (JBI) cohort framework rather than ROBINS-I [46]. Animal studies were assessed across the 10 domains of the Systematic Review Centre for Laboratory Animal Experimentation (SYRCLE) tool, with judgments reported as low, high, or unclear risk and no unsupported overall rating [47]. In vitro and ex vivo studies were evaluated with a structured mechanistic checklist that separated internal mechanistic support from translational distance. Supplementary Table S5A–E provide the complete design-specific appraisals.

Risk of bias was interpreted separately from reporting completeness, statistical precision, indirectness, and translational relevance. Animal and mechanistic studies were therefore not presented as direct evidence of benefit for healthy, physically active adults. Statistical significance in a single study was likewise insufficient to establish an exercise-nutrition effect.

## 3. Results

### 3.1. Literature Search and Study Selection

The five databases yielded 4787 records: 1151 from PubMed/MEDLINE, 398 from Embase Articles, 1228 from Web of Science Core Collection, 1985 from Scopus, and 25 from SPORTDiscus. Automated high-confidence matching removed 2261 duplicates. Manual adjudication of 31 ambiguous duplicate clusters identified 15 additional duplicates, giving a total of 2276 removed records.

After deduplication, 2511 unique records underwent title and abstract screening. We excluded 2200 records and sought 311 reports for full-text retrieval. Despite searches of public sources and manual retrieval attempts, 124 reports could not be obtained; 187 reports underwent full-text eligibility assessment. Of these, 147 were excluded, most commonly because they concerned only infants or young children without an exercise-relevant mechanism (*n* = 46), lacked a link to physical activity (*n* = 33), were not original research or served only as background sources (*n* = 32), lacked adult translational evidence (*n* = 15), or did not investigate HMOs (*n* = 14). Seven additional reports were excluded because they addressed only HMO biosynthesis, provided a protocol without results, had an ineligible design or scope, or used HMOs only as incidental markers. Supplementary Tables S1 and S2 provide report-level details.

**Figure 2:**
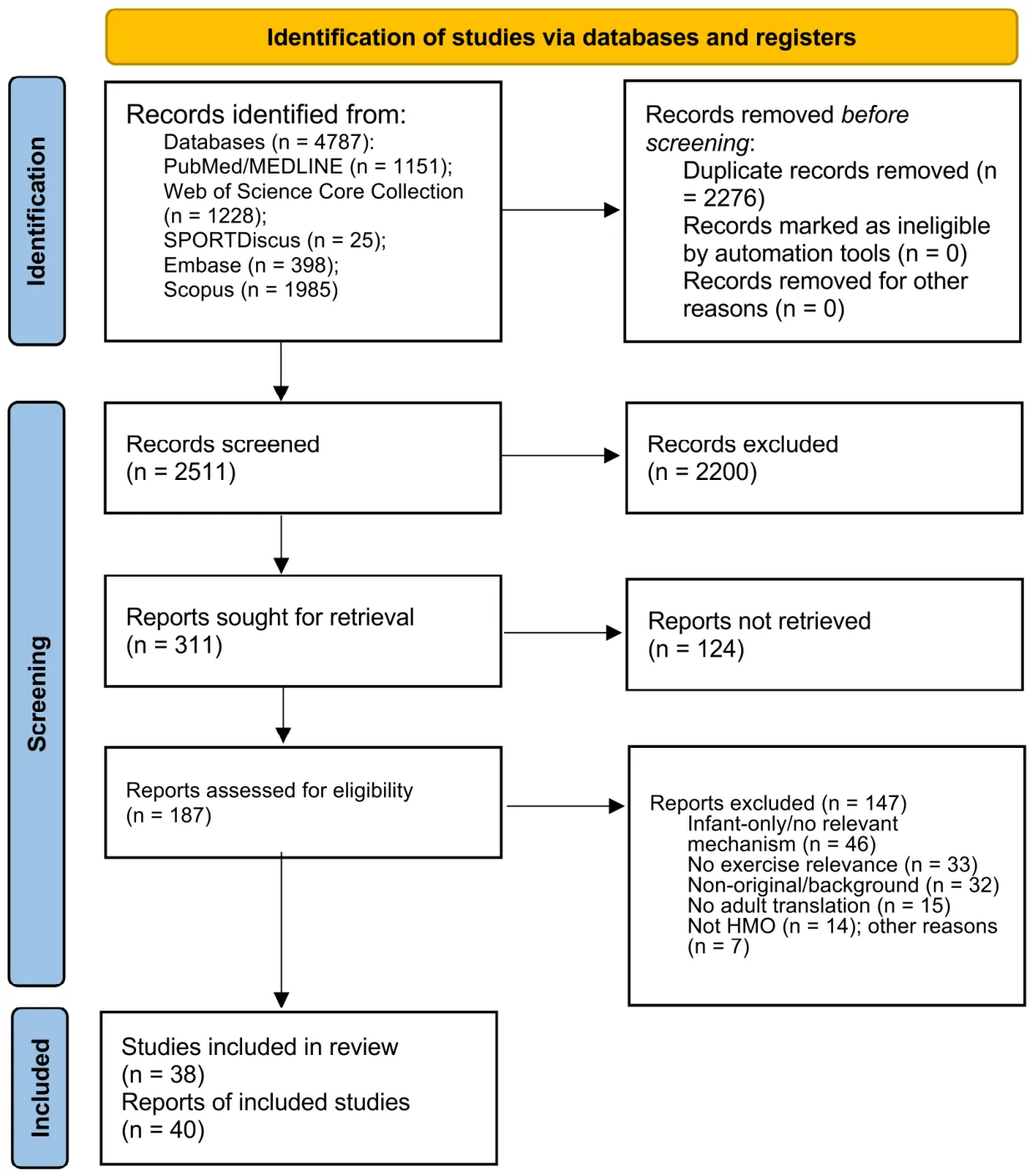
PRISMA flow diagram of literature identification, screening, eligibility assessment, and study inclusion. Five bibliographic databases yielded 4787 records. After removing 2276 duplicates, 2511 unique records were screened, and 311 reports were identified for retrieval. Of the 187 reports assessed in full text, 147 were excluded, and 40 reports representing 38 independent studies were included. Twenty-five trial registrations identified through Embase Clinical Trials were retained separately for verification of ongoing or unpublished evidence and were not included in the bibliographic screening counts. HMO, human milk oligosaccharide. MEDLINE, Medical Literature Analysis and Retrieval System Online; PRISMA, Preferred Reporting Items for Systematic Reviews and Meta-Analyses; n, number of records, reports, or studies as indicated. The underlying flow counts are provided in Supplementary Table S6A.

Forty original research reports were included. Study-family linkage showed that the two Park et al. [32,33] reports published in 2024 arose from one underlying study, as did the two Zhang et al. [48,49] reports published in 2025. The 40 reports therefore represented 38 independent studies. Twenty-five Embase Clinical Trials registrations were retained separately to identify ongoing or unpublished evidence and were excluded from the bibliographic screening counts. Figure 2 presents the PRISMA 2020 study-selection process [42], and Supplementary Table S3 provides the study-family mapping.

### 3.2. Characteristics and Quality Assessment of the Included Evidence

The evidence base included randomized parallel and crossover trials, non-randomized adult interventions, observational studies, animal disease and exercise models, germ-free and human-microbiota-colonized models, cell and organoid studies, and ex vivo fermentation systems. Of the 40 reports, 7 described randomized parallel-group trials and 3 described randomized crossover trials. The remainder comprised non-randomized interventions, observational or multistage human–animal studies, and experimental animal, cellular, organoid, fermentation, or cross-model investigations.

At the independent-study level, 10 studies contributed to Layer A, 22 to Layer B, and 19 to Layer C. Because assignment was non-exclusive, these counts cannot be summed. Exposures included 2′-FL, 3-fucosyllactose (3-FL), 3′-SL, 6′-SL, LNnT, HMO mixtures, and endogenous HMOs. Study duration ranged from a single acute exposure to several months, with substantial variation in population, dose, administration route, exercise paradigm, and outcome definition.

Randomized human evidence was assessed at the outcome level with RoB 2. Four of 10 key outcomes were judged at low risk and 6 raised some concerns; none were judged at high risk. Both non-randomized interventions were judged at serious risk of bias with ROBINS-I, although they retained descriptive value. The longitudinal pregnancy cohort was assessed with the JBI cohort framework. Eighteen animal reports underwent domain-level SYRCLE assessment. No high-risk judgments were identified, but allocation concealment, random housing, caregiver blinding, random outcome assessment, and assessor blinding were often unclear because reporting was insufficient. Nine in vitro, ex vivo, or cross-model studies were assessed with a structured mechanistic checklist. Evidence weight reflected model replication, functional outcomes, perturbation, and rescue, interpreted separately from translational distance to human exercise. Supplementary Table S5A–E provide complete judgments.

Evidence was distributed asymmetrically across the translational chain. Human studies most directly supported short-term tolerability and structure- or host-dependent microbiome responsiveness. Perturbation, knockout, transfer, and rescue experiments strengthened selected intestinal or disease-model mechanisms, but only within those experimental systems. By contrast, healthy-human skeletal-muscle target engagement and reproducible exercise efficacy were not established. This distinction—not the presence of a generic microbiota–metabolite–muscle framework—defines the evidential contribution of the review. Table 1 summarizes direct evidence; Supplementary Tables S4 and S5A–E provide the complete evidence matrix and design-specific appraisals.

### 3.3. Direct Evidence Linking HMOs with Exercise-Related Outcomes

#### 3.3.1. Human Supplementation Studies

Direct evidence from athletes primarily comes from small-sample proof-of-concept studies. Among male recreational athletes undergoing supervised resistance training, 12 weeks of 6′-SL supplementation did not improve the prespecified primary endpoints of body composition, five-repetition maximum strength, or blood lactate levels, nor did it yield a clear between-group benefit in sprint performance. The multivariate group × time test for overall aerobic outcomes was negative (*p* = 0.277), but interactions were observed for several univariate secondary endpoints, yielding exploratory signals in comparisons such as relative peak oxygen consumption and fat oxidation at week 6; by week 12, these signals did not form a consistent favorable pattern, and ventilatory anaerobic threshold (VANT) and its corresponding oxygen uptake (VO_2_) were actually lower. The study employed Fisher’s least significant difference (LSD) test for numerous pairwise comparisons and interpreted values where 0.05 < *p* < 0.10 as trends; no family-level adjustment for multiple comparisons was performed. Therefore, this study supports further validation of the prespecified metabolic endpoints but does not confirm that 6′-SL provides training benefits [37].

In a study of overweight adults simultaneously undergoing step-count targets and energy restriction, the 2′-FL group experienced a similar degree of weight loss as the control group. The overall group × time test for the prespecified set of primary body composition outcomes was negative (*p* = 0.760), and no significant interactions were observed for individual body composition measures; fat mass reduction at week 12 was significant only within the 2′-FL group, while neither body fat percentage nor lean body mass retention showed significant between-group effects. The group × time test for the composite of aerobic capacity was also negative (*p* = 0.452), and there was no significant interaction for resting fat oxidation. Whole-body bone mineral content measured by dual-energy X-ray absorptiometry in the 2′-FL group decreased slightly from baseline (−22.9 g, *p* = 0.049), but the between-group difference was not statistically significant (*p* = 0.082). This was an aggregate bone measure, not a reported decrease in specific mineral concentrations. Due to the combined lifestyle intervention, multiple primary and secondary endpoints, Fisher’s LSD post hoc comparisons, and the lack of family-level adjustment for multiplicity, some of the favorable statements in the abstract and discussion should be downgraded to within-group or exploratory patterns rather than treatment-group effects [38].

#### 3.3.2. Animal and Experimental Exercise Models

The most comprehensive evidence for exercise effects in animal studies comes from two study reports within the same mouse study family. Oral administration of 6′-SL was associated with greater running capacity to exhaustion, grip strength, muscle mass, and muscle fiber size, accompanied by increased MHC; another study report observed reduced blood lactate after exercise and elevated expression of slow-twitch MHC and OXPHOS proteins. Both study reports share the same cohort and protocol and should be regarded as complementary phenotypes of a single study rather than two independent replicates; changes in protein expression also do not prove that the corresponding pathways are necessary for changes in exercise performance [32,33].

In a dexamethasone-induced muscle atrophy model, 6′-SL preserved MHC, muscle morphology, and exercise performance, and attenuated the responses of myostatin, muscle RING-finger protein-1 (MuRF1), and atrogin-1; in middle-aged forced-swimming mice, 2′-FL was associated with improvements in muscle mass, thickness, and strength, accompanied by changes in muscle damage, oxidative stress, PI3K/protein kinase B alpha (AKT1), and protein degradation markers [34,39]. These results support a candidate role in muscle protein homeostasis; however, disease-induced muscle atrophy and forced-swimming stress cannot be equated with adaptive training in healthy adults.

Studies in *Caenorhabditis elegans* have shown that both 3′-SL and 6′-SL reduce swimming-related exhaustion, and mutant experiments suggest that the response depends on AMPK and adenosine receptor signaling [35]. This design enhances the internal causal credibility of the model, but questions regarding mammalian exposure, tissue distribution, and functional relevance in humans remain unresolved. Studies using streptozotocin-induced metabolic stress models and high-fat diet mice provide exercise-related evidence regarding liver–muscle metabolism, energy expenditure, or spontaneous activity, but lack controlled training endpoints [49,50].

#### 3.3.3. Exercise as an Upstream Regulator of HMOs

Harris et al. [39] found that maternal exercise before and during gestation increased milk 3′-SL. Cross-fostering to trained dams improved adult offspring adiposity, glucose regulation, and cardiac ejection fraction, with sex-dependent responses. Global *St3gal4* deletion, disrupting the sialyltransferase required for 3′-SL synthesis, removed the additional benefits of maternal exercise; trained knockout dams also failed to confer these benefits to wild-type pups. Supplementation of 3′-SL during nursing increased circulating 3′-SL and improved adult glucose regulation mainly in males and cardiac function mainly in females [40]. These loss-of-function and supplementation findings support milk 3′-SL as a mediator of lactational programming, but do not establish its downstream receptor or metabolite pathway or efficacy in adult exercisers.

A corresponding randomized crossover trial in humans found no significant changes in 3′-SL or 6′-SL concentrations in breast milk during a 4-h post-exercise observation window following either moderate-intensity continuous exercise or high-intensity interval training [41]. This result limits inferences regarding rapid exercise-induced changes in these two HMOs but does not rule out effects of long-term training, other HMO structures, or different postpartum stages.

#### 3.3.4. What the Direct Evidence Currently Supports

**Figure 3:**
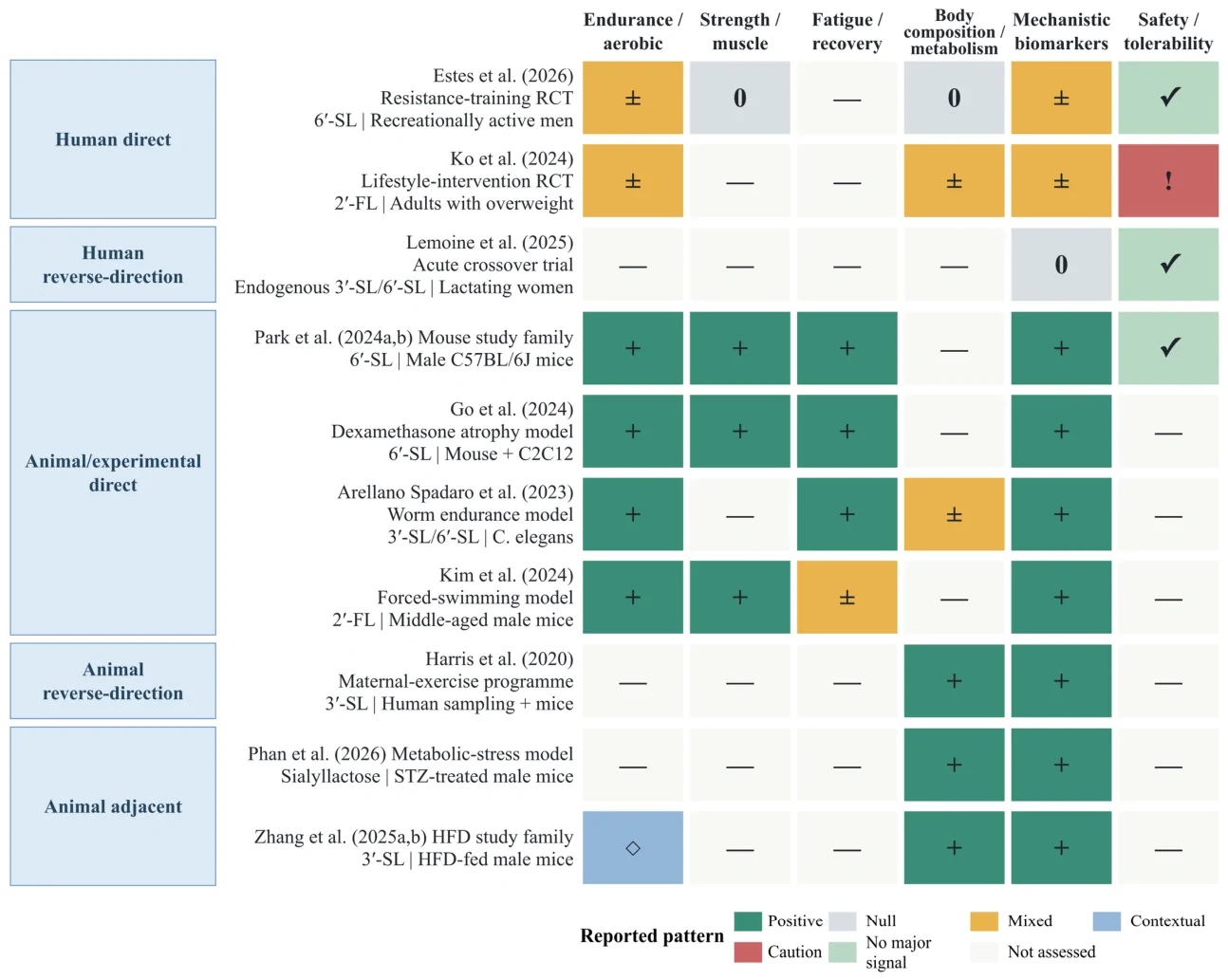
Direction-of-evidence map for direct and exercise-adjacent HMO outcomes. Rows represent independent studies or study families, and columns represent prespecified outcome domains. Cell colors and symbols describe the reported pattern within each study: positive (+), null (0), mixed (±), exercise-adjacent or indirect contextual evidence (◇), a safety finding requiring caution (!), no major safety signal reported within the study (✓), or not assessed (—). Direction codes summarize within-study outcome patterns and do not represent standardized effect sizes, statistical weights, or cross-study vote counts. The two reports by Park et al. [32,33] are displayed as a single mouse study family. The two reports by Zhang et al. [49,50] are also treated as a single study family, with the exercise-adjacent pattern derived from the report containing the relevant activity and calorimetry outcomes. The other studies shown are Estes et al. (2026) [37], Ko et al. (2024) [38], Lemoine et al. (2025) [41], Go et al. (2024) [34], Arellano Spadaro et al. (2023) [35], Kim et al. (2024) [39], Harris et al. (2020) [40], and Phan et al. (2026) [50]. Human trials are placed above experimental models to make the boundary of translational evidence explicit. 2′-FL, 2′-fucosyllactose; 3′-SL, 3′-sialyllactose; 6′-SL, 6′-sialyllactose; *C. elegans*, *Caenorhabditis elegans*; C2C12, mouse myoblast cell line; C57BL/6J, an inbred mouse strain. HFD, high-fat diet; HIIT, high-intensity interval training; HMO, human milk oligosaccharide; RCT, randomized controlled trial; STZ, streptozotocin. The underlying direction codes are provided in Supplementary Table S6B.

Direct evidence comprised three distinct lines of inquiry: small adult supplementation studies with mixed findings, muscle and endurance phenotypes in non-human models, and exercise as an upstream regulator of HMOs. The adult trials do not establish an ergogenic effect: their prespecified primary or overall interaction tests were generally null, and favorable signals were largely secondary, interim, within-group, descriptive, or post hoc. Animal and mechanistic studies identify plausible targets and priority endpoints but cannot upgrade this human efficacy judgment. Current direct evidence therefore supports further structure-specific testing, not a class-wide efficacy claim or practical recommendation for performance enhancement (Table 1; Figure 3).

### 3.4. Gut-Dependent Mechanisms Relevant to Exercise Adaptation

#### Microbial Responses Are Structure- and Host-Dependent

Some HMOs act as selective fermentation substrates for adult-derived microbiota, but the responding taxa, metabolites, and effect magnitude depend on HMO structure, dose, host age, and baseline community composition. In mice colonized with human microbiota, HMO dose altered the steady-state abundance of specific *Bifidobacterium* species and the production of lactate, acetate, and indole metabolites [51]. Fermentation studies using adult-derived microbiota likewise showed age- and structure-dependent differences in responding taxa, cross-feeding, and SCFA kinetics [14,52,53].

Sialylated HMOs do not necessarily produce the *Bifidobacterium*-dominant response often reported for other structures. In the Simulator of the Human Intestinal Microbial Ecosystem (SHIME), a dynamic in vitro gut model that reproduces digestion and colonic fermentation using adult fecal microbiota, 3′-SL and 6′-SL increased propionate and butyrate alongside other community changes, and the resulting fermentation supernatants improved Caco-2 barrier indices [54]. *Bifidobacterium* abundance alone therefore cannot represent the biological response to all HMOs.

An adult microbiota–epithelium model showed that 2′-FL, LNnT, and their combination increased *Bifidobacterium* and SCFAs. Fermentation supernatants reduced paracellular permeability, modified claudin expression, and lowered interleukin-6 (IL-6), with broadly concordant findings across SHIME, Caco-2, and colon-organoid-chip systems [28]. A separate study used fecal microbiota transplantation and pantothenate rescue to strengthen causal support for a local microbial-metabolism-to-barrier pathway [29]. It did not show that one metabolite explained the entire effect or connect the pathway to exercise or muscle outcomes.

In cell, organoid, and mouse models, 2′-FL increased a disintegrin and metalloproteinase 17 (ADAM17) activity and promoted heparin-binding epidermal growth factor-like growth factor (HB-EGF)–epidermal growth factor receptor (EGFR)/extracellular signal-regulated kinase (ERK) signaling. Neutralization, EGFR deletion, and intestinal epithelial-specific deletion attenuated or abolished barrier protection [30]. This pathway has unusually strong within-model causal support for a local intestinal mechanism. It has not yet been tested alongside exercise-induced barrier stress, circulating mediators, skeletal-muscle adaptation, and performance within one experimental framework.

### 3.5. Systemic Availability and Extra-Intestinal Mechanisms

After oral administration of 2′-FL, 6′-SL, and LNnT to adult rats, intact compounds were detected in plasma and excreted in urine, supporting limited systemic availability [55]. In a randomized trial of healthy older adults, supplemented 2′-FL was detected in plasma and urine in a subset of participants [14]. The primary cytokine-response endpoint was null, while most other circulating outcomes were secondary or derived from responder analyses. Multiple endogenous HMOs were also detectable in serum during pregnancy [56]. This observation establishes physiological plausibility for circulating HMOs but cannot substitute for oral pharmacokinetic evidence in the general adult population.

In a GNE (UDP-N-acetylglucosamine 2-epimerase/N-acetylmannosamine kinase) myopathy model, long-term oral 6′-SL produced measurable systemic exposure, restored muscle sialylation, improved muscle size and strength, and preserved spontaneous activity [30]. Dose–response assessment, activity controls, cellular uptake experiments, pharmacokinetic measurements, and tissue phenotyping strengthened this within-model chain. The findings nevertheless depended on a specific hyposialylation disorder and relatively high doses. They support systemic exposure and muscle effects under pathological conditions, not direct action in healthy adult muscle.

### 3.6. Skeletal Muscle, Mitochondrial, and Substrate Metabolism Pathways

#### 3.6.1. Muscle Structure and Protein Homeostasis

Studies of exercise and muscle atrophy models have repeatedly reported changes in muscle structure and muscle protein homeostasis, including markers related to MHC, slow-twitch MHC, myostatin, MuRF1, atrogin-1, and PI3K/AKT1 [31,32,33,38]. These findings form clusters of mechanisms pointing in a similar direction; however, most involve changes in expression rather than muscle-specific knockouts, pharmacological blockade, or rescue. Studies in aging mice showing that 2′-FL did not improve skeletal muscle mass also indicate that metabolic or gut benefits do not necessarily translate into muscle gains [57].

#### 3.6.2. Mitochondrial Function and Substrate Utilization

The human 6′-SL training study found no effect on prespecified primary endpoints or the overall aerobic group × time test. Signals were confined to several interim secondary outcomes without multiplicity adjustment and were not consistent at the end of the intervention [37]. By contrast, the mouse study family reported increased slow-type MHC and OXPHOS proteins, while *Caenorhabditis elegans (C. elegans)* mutant experiments indicated that endurance responses required AMPK and adenosine-receptor signaling and coincided with altered substrate use [34,35]. AMPK–peroxisome proliferator-activated receptor gamma coactivator 1-alpha (PGC-1α) signaling associated with 2′-FL in adipocytes offers additional context, but the high in vitro concentrations, tissue differences, and absence of direct respiration measurements limit extrapolation to skeletal muscle [58].

#### 3.6.3. Oxidative Stress, Inflammation, and Cross-Organ Metabolism

Disease, inflammatory, and aging models have reported changes in nuclear factor erythroid 2-related factor 2 (Nrf2), nuclear factor kappa B (NF-κB), GPR43, AMPK/sirtuin 1 (SIRT1), antioxidant enzymes, and inflammatory markers, generally without testing pathway necessity [59,60,61,62]. Gut–liver–muscle, gut–brain, and systemic energy links in aging, streptozotocin, and high-fat-diet models rely mainly on parallel biomarkers and omics networks [48,49,50,63]. Layer B therefore supports local barrier mechanisms and selected systemic-to-muscle effects in disease models. The chain from HMO exposure to healthy adult muscle and training adaptation remains unverified (Table 2).

### 3.7. Adult Translation: Safety, Microbiome Responsiveness, and Host Outcomes

#### 3.7.1. Safety and Tolerability

In adult interventions, safety and gastrointestinal tolerability are outcomes supported relatively consistently. Healthy adults supplemented with 6′-SL at 6 g/day for 12 consecutive weeks did not experience any serious adverse reactions or clinically significant abnormalities in liver or kidney function; 2′-FL, LNnT, and their combinations were also generally well tolerated in short-term trials [11,12]. Other randomized studies in adults did not identify any clear signals of serious safety concerns [13,15,64,65,66]. These data only support “overall good tolerability at the studied doses and durations” and cannot be extrapolated to long-term use, all populations, high-intensity exercise conditions, or rare adverse events.

#### 3.7.2. Microbiome Responsiveness

Randomized trials in adults consistently support that HMOs can alter specific microbial communities. 2′-FL and LNnT increase Actinobacteria or *Bifidobacterium* in healthy adults; the increase in *Bifidobacterium* associated with high-dose 2′-FL in healthy older adults declined during the washout period; signs of 2′-FL-induced microbiome resilience following vancomycin-induced disruption were primarily observed early on and did not persist by week 8 [11,13,64]. In a crossover trial for ulcerative colitis, 2′-FL increased *Bifidobacterium* but did not improve the primary endpoint; in a trial of working adults, the independent host effect of 2′-FL alone was also limited [65,66].

Uncontrolled studies and human ex vivo studies have provided additional information regarding dose, age, and baseline microbiome dependence; however, they cannot support equivalent causal inferences [14,52,53,54,67,68]. Therefore, future exercise trials in adults require baseline microbiome stratification and repeated sampling at multiple time points; changes in a single bacterial genus should not be directly interpreted as a marker of exercise responsiveness.

#### 3.7.3. Host Metabolic Effects and the Translational Gap

Metabolic findings in adult hosts are less robust than those regarding the microbiota. The primary cytokine-response score was null in trials involving healthy older adults, and early microbiota signals observed in antibiotic perturbation studies did not translate into sustained improvements in SCFAs, glucagon-like peptide-1 (GLP-1), insulin sensitivity, or most metabolic markers [13,64]. In an acute crossover study, 2′-FL increased some circulating SCFAs, but the effect depended on common substrates or specific phenotypes, and the source of circulating metabolites could not be directly determined [15]. In lifestyle co-interventions, group × time analyses of key body composition measures, resting substrate oxidation, and aerobic capacity did not reveal clear treatment effects; corresponding favorable signals should be considered within-group or exploratory patterns and cannot be completely separated from dietary and activity prescriptions [38].

Overall, Layer C supports preliminary evidence that selected HMOs are tolerated by adults, produce structure- and host-dependent microbiome responses, and can achieve limited systemic availability. It does not establish sustained metabolic benefit, healthy-muscle target engagement, or enhanced exercise performance. The evidence chain fails at four successive transitions: structure to dose–exposure, exposure to target engagement, target engagement to a temporally ordered muscle phenotype, and muscle phenotype to reproducible functional benefit. Table 3 summarizes the adult translational evidence.

## 4. Discussion

### 4.1. Key Findings and Translational Evidence Boundaries

Current evidence supports a conclusion narrower than efficacy. Selected HMOs are biologically active and testable in adults, but are not established ergogenic aids. Tolerability and structure- or host-dependent microbial responses are supported more consistently than systemic target engagement, healthy-muscle effects, or exercise benefit; findings cannot be generalized across the HMO class.

Direct human efficacy remains sparse and inconclusive. In the 6′-SL resistance-training study, primary endpoints and overall aerobic interactions were null, with favorable signals limited to interim or secondary comparisons [37]. The 2′-FL lifestyle study likewise showed null key interaction tests, and favorable interpretations were mainly exploratory [38]. These findings support better-powered replication, not a general performance, body-composition, or training-adaptation benefit.

Mechanistic evidence is stronger within selected models than across translation. Perturbation, knockout, transfer, or rescue can support causal claims within those systems, but not in healthy exercisers without joint evidence of exposure, tissue engagement, temporal mediation, and functional consequence. Changes in AMPK, OXPHOS, PI3K/AKT, MHC, or protein-degradation markers remain mechanistic leads rather than proof of pathway necessity.

**Figure 4:**
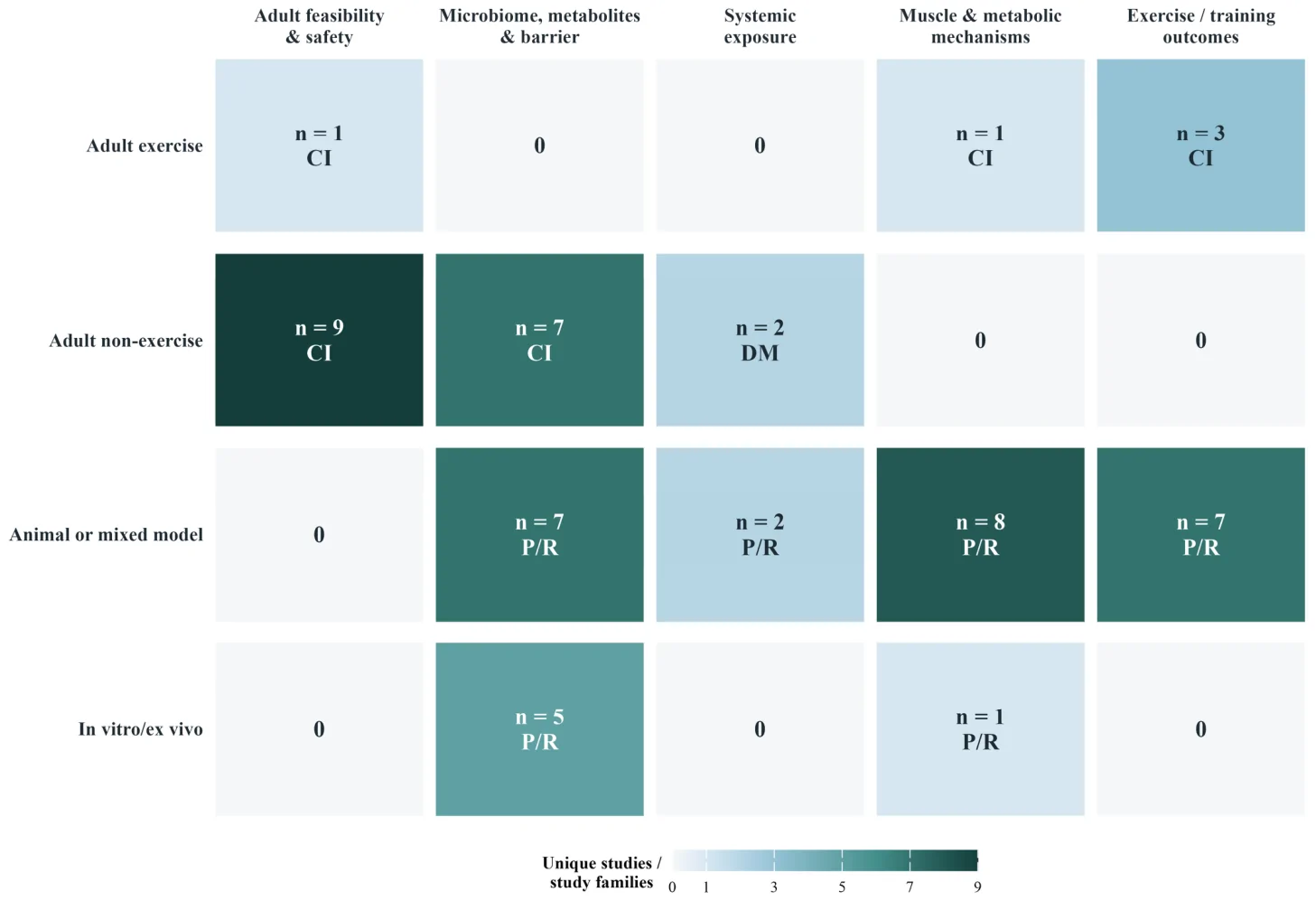
Translational evidence ladder and causal-strength matrix for HMO–exercise research. Columns represent successive evidence stages ranging from adult intervention feasibility and gut-local responses to systemic exposure, skeletal-muscle mechanisms, and exercise or training outcomes. Rows distinguish adult exercise studies, adult non-exercise studies, animal or mixed models, and in vitro or ex vivo systems. Cell fill represents the number of unique studies or study families contributing evidence to that cell; counts are non-exclusive across stages and should not be summed. Cell annotations indicate the highest causal-support class represented: P/R, functional perturbation, transfer, genetic deletion or rescue; CI, controlled intervention; DM, direct measurement without a causal intervention; A/I, association or indirect evidence. The causal-support class does not replace study-level risk-of-bias assessment and does not indicate certainty in humans. The matrix highlights a translational bottleneck between gut-local or preclinical evidence and directly verified effects on muscle and performance in healthy humans. HMO, human milk oligosaccharide. n, number of unique studies or study families contributing to a cell. Underlying cell counts are provided in Supplementary Table S6C.

This review identifies tested HMO structures, evaluates which human efficacy claims survive endpoint-level scrutiny, and separates within-model causal support from healthy-human evidence. It cannot estimate a class-wide effect or recommend an HMO for performance. A pooled analysis would obscure null primary endpoints, related study families, structural heterogeneity, and translational distance; the A/B/C synthesis preserves these boundaries (Figure 4).

### 4.2. Structure-Specific Pathways: Within-Model Causality Versus Human Translation

The microbiota–metabolite–muscle axis is established background rather than this review’s principal novelty [70,71,72,73]. The HMO-specific question is whether individual structures modify that axis reproducibly. Adult, ex vivo, and human-microbiota-colonized studies show that local microbial and barrier responses depend on structure, dose, age, baseline community, and sampling time [11,13,14,51,52,53,54]. Fermentation supernatants, microbiota transfer, and metabolite rescue strengthen selected microbiota-to-barrier mechanisms within models [28,29]. None shows that the same mediator is necessary for muscle adaptation during exercise.

For 2′-FL, neutralization and receptor or epithelial-specific knockout support ADAM17–HB-EGF–EGFR/ERK signaling as a local barrier mechanism [30]. However, these intestinal-injury models did not integrate exercise stress, circulating mediation, muscle target engagement, and performance. Permeability interpretation also depends on the marker, sampling protocol, and clinical context [74]. The evidence therefore supports within-model intestinal causality, not a validated human ergogenic pathway.

Systemic evidence is more restricted. Plasma or urinary detection of orally administered 2′-FL, 6′-SL, or LNnT establishes limited bioavailability, not an effective concentration in healthy muscle [13,55]. In GNE myopathy, 6′-SL exposure, muscle sialylation, and functional improvement form a comparatively complete within-model chain [31], but this pathological state is not representative of healthy exercisers. Broader SCFA and gut–muscle research provides biological context [75,76]; it does not demonstrate HMO-specific mediation.

Gut-dependent and systemic pathways may converge on inflammation, redox balance, mitochondrial function, substrate use, and protein homeostasis, but their relative contributions remain unresolved. Causality requires tests of pathway necessity within a model; human translation additionally requires structure-specific exposure, temporal target engagement, muscle mediation, and a prespecified functional outcome.

Galactooligosaccharides (GOSs) and fructooligosaccharides (FOSs) may reproduce some prebiotic and immunomodulatory functions associated with HMOs, but overlapping functions do not establish structural or biological equivalence [77]. Commercial β-GOSs are predominantly lactose-derived galactose oligomers whose linkages influence microbial utilization [78]; they do not reproduce the structural diversity of fucosylated and sialylated HMOs. Their sports-nutrition relevance is supported by some, but not consistent, human findings. A 24-week GOS trial in elite rugby players reported shorter upper respiratory symptom duration and lower gastrointestinal symptom incidence and severity [79], whereas a 12-week pilot trial in recreational endurance athletes found no group-level benefits for gastrointestinal symptoms or general wellness [80]. These outcomes concern athlete health rather than demonstrated gains in exercise performance, and neither trial establishes equivalence to HMOs. GOSs/FOSs therefore merit evaluation as comparators or complementary prebiotics, not established HMO substitutes. Their use requires formulation-specific assessment of gastrointestinal tolerance and prespecified functional outcomes; effects observed with GOSs should not be assumed for FOSs or transferred to HMOs.

### 4.3. Structure Specificity and Four Translational Bottlenecks

The first bottleneck is structure to exposure. Fucosylated, neutral non-fucosylated, and sialylated HMOs differ in microbial use, receptor interactions, absorption, and host effects and are not interchangeable [1]. Exercise studies have tested 2′-FL, 3′-SL, and 6′-SL across dissimilar populations and protocols, with almost no head-to-head comparison. Structure-specific dose–concentration relationships at rest and during exercise are required before considering a class effect.

The second bottleneck is exposure to target engagement. Plasma or urinary detection does not establish skeletal-muscle delivery or target modulation. Sex, age, diet, training status, baseline microbiota, disease state, and exercise timing may modify exposure and response [13,14,52,53,54,64,65,66,67,69]. Future studies should measure intact HMOs and candidate metabolites with tissue-proximal markers, prespecify modifiers, and avoid post hoc responder definitions. Potential interactions between HMOs and dietary polyphenols through microbial, epithelial, and immune pathways have been discussed primarily in the allergy-focused literature [81]. Adult studies of 2′-FL with or without resistant starch also highlight the need to consider co-administered fermentable substrates when interpreting circulating SCFA responses [15]. Future trials should record and, where feasible, standardize polyphenol and fermentable-fiber intake. Claims of synergy require designs that distinguish the effects of each component from their combination, rather than inference from overlapping pathways. Current evidence does not establish that HMO–polyphenol combinations improve exercise performance.

The third bottleneck is target engagement to muscle phenotype. Existing models and interventions use incompatible time scales and outcome windows [32,33,35,37,38,39,40,41]. Mediation requires that a biomarker follows exposure, precedes or coincides with the muscle response, and weakens after pathway disruption. Human trials therefore need repeated sampling with concurrent assessment of exposure, mediators, and muscle function.

The fourth bottleneck is muscle phenotype to functional benefit. Changes in AMPK, OXPHOS, substrate use, protein turnover, or body composition cannot substitute for prespecified performance or training adaptation [82,83,84]. Existing studies used heterogeneous outcomes, small samples, repeated time points, and numerous secondary comparisons. Future trials require core outcomes, meaningful effect thresholds, adverse-event reporting, adequately powered between-group tests, and independent replication.

### 4.4. Future Directions and Falsifiable Hypotheses

**Figure 5:**
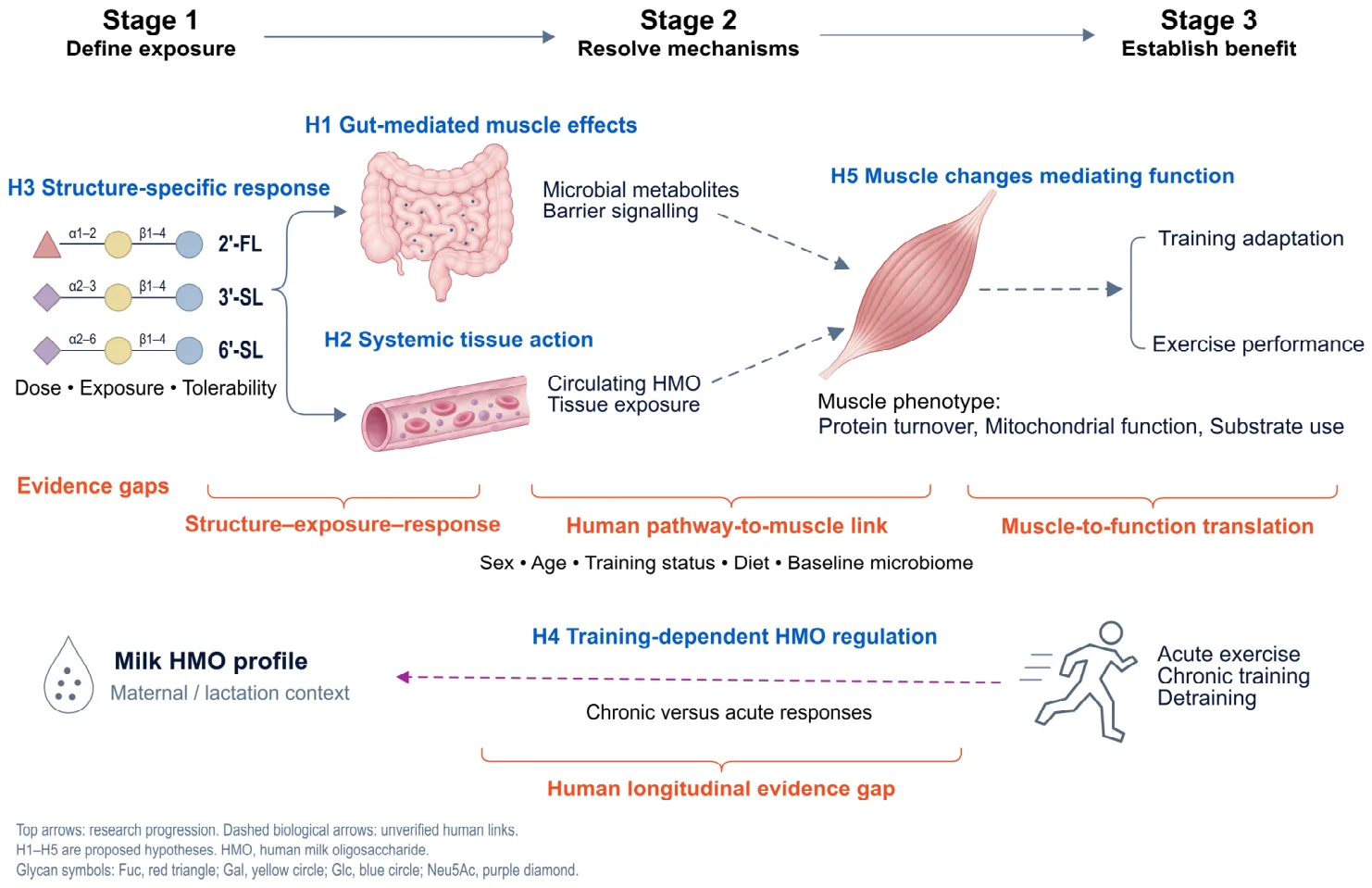
Falsifiable, gated roadmap addressing the four translational bottlenecks in HMO–exercise research. Stage 1 resolves structure to dose–exposure by defining structure-specific safety, tolerability, and pharmacokinetics at rest and during exercise. Stage 2 tests exposure to target engagement and target engagement to muscle phenotype through temporally ordered measurements of microbiome function, microbial and circulating metabolites, intestinal barrier status, intact HMO exposure, and validated muscle outcomes, with perturbation or prespecified mediation where feasible. Stage 3 tests whether a proximal muscle phenotype translates into a reproducible functional benefit in adequately powered, preregistered randomized trials. H1–H5 are falsifiable hypotheses rather than established mechanisms, and dashed arrows denote unverified human links. Sex, age, training status, diet, and baseline microbiome are prespecified effect modifiers. HMO, human milk oligosaccharide; PK, pharmacokinetics; RCT, randomized controlled trial. 2′-FL, 2′-fucosyllactose; 3′-SL, 3′-sialyllactose; 6′-SL, 6′-sialyllactose; Fuc, fucose; Gal, galactose; Glc, glucose; Neu5Ac, N-acetylneuraminic acid.

A gated translational program should precede large efficacy trials. Stage 1 should establish structure-specific safety and pharmacokinetics at rest and during exercise. Stage 2 should test temporally ordered gut-dependent and systemic pathways with concurrent exposure, mediator, and muscle measurements. Stage 3 should proceed only after target engagement and consistent functional signals, using adequately powered randomized trials with preregistered performance or training outcomes and independent replication (Figure 5).

Five competing hypotheses follow from this framework (Table 4). H1, the gut-dependent hypothesis, predicts that specific HMOs affect skeletal muscle or recovery through microbial function, metabolites, and barrier status. Persistence of the effect after microbiota depletion or disruption of key metabolite transport would weaken H1. H2, the systemic hypothesis, predicts that intact HMOs or host-derived products enter the circulation and act outside the gut independently of the microbiota. It requires structure-specific pharmacokinetic and pharmacodynamic relationships, evidence of tissue exposure, and effects that persist after the intestinal pathway is controlled. H3, the structure-specific hypothesis, predicts that 2′-FL, 3′-SL, and 6′-SL have non-interchangeable exercise-related effects. Reproducibly equivalent effects in standardized head-to-head studies would weaken H3.

H4, the temporal exercise-to-HMO hypothesis, predicts that exercise-related changes in milk HMOs reflect chronic training or pregnancy–lactation adaptation more strongly than a single acute session. It requires within-cohort time-series comparisons across acute exercise, sustained training, and detraining. H5, the muscle-phenotype hypothesis, predicts that muscle protein homeostasis, slow-fiber or OXPHOS characteristics, and substrate utilization are more stable proximal outcomes than broad improvements across performance domains. Failure of these markers to precede, mediate, or predict functional change would narrow or reject H5.

These hypotheses define conditions for rejection. Future studies should include diverse exercise populations, prespecified endpoints, complete reporting, and longer safety follow-up. Athlete-microbiome guidance similarly recommends evaluating substrate specificity, dose, safety, and functional endpoints within one evidence chain rather than treating microbiome change as efficacy [85]. Exercise-nutrition applications should advance only after exposure, mechanism, and performance findings are independently reproduced.

## 5. Strengths and Limitations

This structured review addresses an emerging, heterogeneous topic through a structure-specific, endpoint-level audit that separates human efficacy from model-internal causality. Reporting 40 reports as 38 independent studies avoided treating related publications as replications. The A/B/C framework and design-specific appraisal distinguished direct, mechanistic, and translational evidence while limiting causal overstatement.

Direct human exercise evidence remains sparse, small, and inconsistent. Trained athletes, women, and adults across the lifespan are underrepresented. Favorable secondary or exploratory findings must be interpreted against null primary endpoints and multiplicity, while small samples reduce precision, subgroup assessment, and detection of uncommon adverse events.

Evidence was heterogeneous in HMO structure, dose, route, duration, species, disease state, exercise modality, sampling, and outcomes. Disease, forced-swimming, atrophy, aging, and invertebrate models inform mechanisms but do not reproduce adaptive training in healthy humans. A stratified narrative synthesis was therefore preferable to a pooled estimate, but cannot provide a summary effect or formal heterogeneity statistic.

Full-text access was incomplete: 124 of 311 reports sought could not be obtained, creating potential availability bias despite documented retrieval attempts and exclusions. The prespecified HMO × exercise scope also excluded comprehensive synthesis of infant clinical nutrition, improving focus but limiting conclusions about the broader early-life literature.

Publication and selective-reporting bias remain plausible because positive findings may receive more attention and multiple outcomes permit selective emphasis. Randomized human outcomes were generally at low risk or raised some concerns, whereas non-randomized evidence had serious confounding and attribution problems. In animal studies, reporting of housing, concealment, blinding, and prespecified analyses was often insufficient; unclear SYRCLE judgments therefore limit confidence without confirming high risk.

The dual-pathway framework and H1–H5 hypotheses organize evidence but are not fully validated causal models. Selected local and disease-model pathways have within-model support, yet no complete chain links oral HMO exposure to mediation, healthy muscle, and exercise performance. Hypotheses should be narrowed or rejected if temporal order, dose–exposure relationships, mediator necessity, and independent reproducibility are not confirmed.

## 6. Conclusions

HMOs are structurally diverse glycans with emerging relevance to exercise nutrition. This review assessed structure-specific human efficacy and distinguished model-internal causal evidence from adult translational support.

Selected HMOs are tolerated at the adult doses and durations studied, can alter microbial responses, and may achieve limited systemic exposure. Some intestinal and disease models support causal mechanisms, but direct human exercise evidence remains sparse and inconclusive. Favorable exploratory signals do not establish reproducible performance benefits. Translation remains limited by four unresolved links: structure to exposure, exposure to target engagement, target engagement to muscle phenotype, and muscle phenotype to functional benefit.

Future research should establish structure-specific safety and pharmacokinetics at rest and during exercise, test temporally ordered mechanisms, and use adequately powered trials with prespecified functional endpoints and independent replication. HMOs remain research candidates, not an established class of ergogenic aids. Practical recommendations require reproducible efficacy and tolerability for defined compounds, doses, and exercise populations.

## Figures and Tables

**Table 1:** Direct and exercise-adjacent evidence linking HMOs with exercise-related phenotypes.

Study and Design	HMO Exposure and Exercise Context	Principal Outcome Pattern	Critical Evidence Boundary	Synthesis Confidence
Estes L et al. [37] Double-blind, placebo-controlled proof-of-concept trial; 19 recreationally active men	6′-SL, 900 mg/day for 12 weeks Exercise: Supervised resistance training	Primary outcomes showed no added benefit for body composition, 5RM strength, or blood lactate; sprint outcomes were also null. The omnibus aerobic group-by-time test was null, although several univariate secondary outcomes and week-6 contrasts generated transient signals	Very small male-only sample of study completers; numerous outcomes and Fisher LSD comparisons without family-wise multiplicity control; secondary signals were not sustained	Low–moderate
Ko J et al. [38] Double-blind, placebo-controlled trial; 41 sedentary adults who were overweight	2′-FL, 3 g/day for 12 weeks Exercise: Step target plus energy-restriction program in both groups	Weight loss was comparable. The omnibus primary body composition group-by-time test and the aerobic capacity interaction were null; favorable changes in fat mass, fat-free mass, fat oxidation, and aerobic capacity were predominantly within-group, descriptive, or post hoc. A small decrease in bone mineral content (BMC) was observed in the 2′-FL group	Combined lifestyle intervention; multiple primary and secondary outcomes; Fisher LSD comparisons without family-wise multiplicity control; several claims were not supported by significant between-group interactions	Low–moderate
Lemoine MCC et al. [41] Randomized three-condition crossover trial; 20 lactating women	REST versus MICT versus HIIT; no HMO supplementation Exercise: Acute endurance exercise	No clear acute change in milk 3′-SL or 6′-SL from immediately after exercise to 4 h later	Tests exercise→HMO rather than HMO → performance; acute exposure, two HMOs, and no downstream functional outcome	Moderate–high
Park EJ et al. [32]/Park EJ et al. [33] Two reports from a single controlled mouse study; 20 male C57BL/6J mice	6′-SL, 100 mg/kg orally, 5 days a week for 12 weeks Exercise: Exhaustive treadmill testing, grip strength, and post-exercise metabolic recovery	Greater running capacity, grip strength, muscle mass and fiber size, and MHC expression; lower post-exercise lactate levels; increased slow-MHC and OXPHOS expression	Single sex, species, and dose; small outcome-specific samples; pathway necessity not tested	Moderate
Harris JE et al. [40] Multistage human observational and mouse mechanistic program	Maternal exercise-associated 3′-SL; knockout, cross-fostering, and lactational rescue experiments Exercise: Maternal exercise and offspring metabolic/cardiac phenotypes	Mouse loss-of-function and rescue studies supported an Exercise → milk 3′-SL→offspring phenotype pathway	Reverse causal direction relative to supplementation for athletes; the human component did not establish downstream outcomes; possible global-knockout pleiotropy	Moderate–high internal consistency within animals; low for human outcomes
Go H et al. [34] C2C12 myotubes and mice with dexamethasone-induced muscle atrophy	6′-SL; oral dosing in mice included 100 mg/kg Exercise: Performance assessed in an atrophy model rather than a training study	Preserved MHC, myotube morphology, muscle size, and exercise performance; reduced myostatin, MuRF1, and atrogin-1 responses	Disease/atrophy model; incomplete reporting of allocation and blinding; no direct pathway blockade	Moderate
Arellano Spadaro J et al. [35] *C. elegans* wild-type and signaling-mutant experiments	3′-SL and 6′-SL dietary supplementation Exercise: 120-min swimming/endurance paradigm	Both sialyllactoses reduced exhaustion; the benefit depended on AMPK and adenosine receptor signaling and was accompanied by a metabolic shift	Strong evidence of perturbation within the model, but a major gap in translation from invertebrates to humans and in dose extrapolation	Moderate within-model evidence; very low translational relevance
Phan LP et al. [50] STZ-induced metabolic stress mouse study; *n* = 5 per group	Sialyllactose/6′-SL, 25 or 75 mg/kg pretreatment Exercise: Liver–skeletal muscle metabolic axis; no direct exercise test	Improved glucose and hepatic biochemical profiles with protection of the liver and skeletal muscle	Very small groups, prophylactic design, acute disease model, and no performance endpoint	Low
Kim et al. [39] Middle-aged male mouse forced-swimming study	2′-FL, multiple oral doses Exercise: Repeated forced swimming	Improved muscle mass, thickness, and strength; changes in PI3K/AKT1, proteolysis-related markers, oxidative stress, and muscle damage	Multiple endpoints; pathway changes are associative rather than indicative of causality; human-equivalent exposure uncertain	Low–moderate
Zhang et al. [48] High-fat-diet mouse dose–response study	3′-SL, multiple gavage doses Exercise: Spontaneous activity and calorimetry; no prescribed exercise program	Improved metabolic phenotype with higher energy expenditure and activity, and changes in the gut–liver pathway	Activity was a secondary outcome; the dose–response relationship was not monotonic; and this was not a controlled exercise test	Low–moderate

**Abbreviations:** 2′-FL, 2′-fucosyllactose; 3′-SL, 3′-sialyllactose; 5RM, five-repetition maximum; 6′-SL, 6′-sialyllactose; BMC, bone mineral content; HIIT, high-intensity interval training; HMO, human milk oligosaccharide; MHC, myosin heavy chain; MICT, moderate-intensity continuous training; OXPHOS, oxidative phosphorylation; STZ, streptozotocin.

**Table 2:** Mechanistic evidence relevant to HMO-mediated exercise and skeletal muscle adaptation.

Study and Model/Population	HMO/Exposure	Mechanistic Evidence	Within-Model Causal Support	Translational Relevance and Scope
Schalich KM et al. [29] DSS/TNBS mouse colitis, FMT, *B. infantis*, and intestinal epithelial models	2′-FL	Microbiota remodeling, pantothenol/pantothenate, GPX, ZO-1, permeability, and inflammation	FMT transfer and metabolite rescue support a microbiota–metabolite–barrier pathway, but do not establish complete mediation	Strong evidence supporting a local gut pathway; no evidence regarding muscle or exercise outcomes
Kaur H et al. [30] Intestinal epithelial cells, colon organoids, epithelial Egfr knockout mice	2′-FL	ADAM17 activation, HB-EGF release, EGFR/ERK transactivation, preservation of tight junctions, and reduced apoptosis	HB-EGF neutralization and cellular, organoid, and epithelial-specific Egfr loss attenuated the effect	A relatively strong direct epithelial mechanism; downstream gut–muscle and performance links remain untested
Šuligoj T et al. [28] Adult-donor SHIME, Caco-2 monolayers, and colon organ-on-chip models	2′-FL, LNnT, or a combination	Bifidogenic/SCFA responses, reduced permeability, altered claudins, and lower IL-6	Consistency across models, but complex fermentation supernatants prevent attribution to a single metabolite	Supports a fermented HMO–barrier pathway without an exercise challenge
Sato Y et al. [54] Adult-donor SHIME and Caco-2 monolayers	3′-SL or 6′-SL	Propionate/butyrate production, non-bifidogenic community shifts, and improved epithelial barrier readouts	No isolate-specific or metabolite-specific mediation test	Demonstrates that *Bifidobacterium* is not a universal mechanistic marker; the number of donors was small
Reens AL et al. [51] Gnotobiotic mice with adult human microbiota	HMO mixture with or without B. infantis	Dose-dependent B. infantis abundance and changes in lactate, acetate, indole metabolites, and cross-feeding products	Dose–response relationships and controlled microbial addition support conclusions regarding substrate–microbe interactions	Mechanistically informative but based on few donors and no host performance outcome
Firrman J et al. [13]/Bajic D et al. [52]/Sato Y et al. [53] Adult- or age-stratified donor ex vivo fermentation	2′-FL, 3-FL, LNnT, 3′-SL, and/or 6′-SL	Age-, structure-, dose-, and time-dependent microbiota and SCFA responses	Descriptive dose-time patterns without host-level causal testing	Supports responder stratification and repeated sampling; predicted exposure cannot define an effective exercise dose
Vazquez E et al. [55] Adult and suckling rats	Oral administration of 2′-FL, 6′-SL, or LNnT	Plasma detection, urinary excretion, and dose-dependent 2′-FL exposure	Direct pharmacokinetic measurement supports systemic accessibility	Does not establish bioavailability, tissue distribution, or an exercise-effective concentration in adult humans
Carter MM et al. [13] Randomized trial in healthy older adults	2′-FL, 1 or 5 g/day	Plasma/urine detection, transient bifidogenic response, and circulating metabolic/protein markers	Randomization supports exposure comparisons; the primary cytokine endpoint was null	Human systemic-accessibility bridge; no exercise endpoint and several secondary and responder analyses
Jantscher-Krenn E et al. [56] Longitudinal pregnancy cohort	Endogenous circulating HMOs	Sixteen HMOs detected in serum, showing patterns related to gestational stage and secretor status	Direct detection in humans, but observational and physiologically specific	Demonstrates that HMOs can be present in the adult circulation; cannot substitute for oral pharmacokinetics in exercising adults
Yonekawa T et al. [31] GNE-myopathy mice and patient-derived myoblasts	Oral 6′-SL	Circulating exposure, muscle sialylation, muscle size/strength, pathology, and voluntary activity	Dose, NeuAc comparator, uptake, pharmacokinetics, and tissue measurements form a multilevel disease-mechanism chain	Strong disease-specific systemic-muscle evidence; high dose and GNE pathology limit translation to healthy adults
Park EJ et al. [32]/Park EJ et al. [33] A family of linked mouse exercise studies	Oral 6′-SL	Muscle mass/fiber size, total and slow MHC, OXPHOS proteins, lactate recovery, grip strength, and endurance	Complementary phenotyping without pathway blockade	Direct relevance to mouse exercise; the linked reports constitute a single study, not independent replications
Go H et al. [34] C2C12 myotubes and dexamethasone-induced atrophy mice	6′-SL	Preservation of MHC and attenuation of myostatin, MuRF1, and atrogin-1 responses	Consistency across cell and animal models without the need for target-specific testing	Supports muscle protein maintenance in an atrophy model rather than healthy training adaptation
Kim et al. [39] Middle-aged male mice undergoing forced swimming	Oral 2′-FL	Muscle morphology/strength, PI3K/AKT1, proteolysis-related markers, oxidative stress, and tissue damage	Multiple associated pathways; no pathway-specific blockade	Exercise-related muscle support with uncertain human-equivalent exposure and substantial multiplicity
Arellano Spadaro J et al. [35] Wild-type and signaling-mutant *C. elegans*	3′-SL or 6′-SL	AMPK/adenosine-receptor-dependent endurance- -phenotype, mitochondrial changes, and substrate shift	Genetic dependence strengthens causal inference regarding within-model	Significant pathway perturbation within an invertebrate model; very large translational distance
Chen S et al. [58] 3T3-L1 and C3H10T1/2 adipocyte models	2′-FL	p-AMPK, PGC-1α, UCP1, mitochondrial staining, and beige-adipocyte markers	Compound C attenuated responses, but off-target effects and a lack of genetic validation remain	Supports an adipose-energy pathway, not a demonstrated skeletal-muscle mitochondrial mechanism
Li A et al. [57]/Wang J et al. [59] Mice aged naturally or with d-galactose	2′-FL	Changes related to microbiota, the barrier, SCFAs, the immune system, AMPK/SIRT1/PGC-1α, and antioxidants	Predominantly parallel changes without depletion, transfer, receptor blockade, or rescue	Metabolic plausibility only; skeletal muscle mass was not measured in the natural-aging study
Phan LP et al. [50]/Zhang et al. [48]/Zhang et al. [49] STZ- or high-fat-diet mouse models	Sialyllactose/3′-SL	Liver–muscle or gut–liver metabolic, inflammatory, energy expenditure, and activity-associated networks	Primarily correlational molecular and multi-omic evidence	Cross-organ support in disease models; no established causal link to exercise adaptation

Abbreviations: 2′-FL, 2′-fucosyllactose; 3-FL, 3-fucosyllactose; 3′-SL, 3′-sialyllactose; 6′-SL, 6′-sialyllactose; ADAM17, a disintegrin and metalloproteinase 17; AMPK, AMP-activated protein kinase; DSS, sodium dextran sulfate; EGFR, epidermal growth factor receptor; ERK, extracellular signal-regulated kinase; FMT, fecal microbiota transplantation; GNE, UDP-N-acetylglucosamine 2-epimerase/N-acetylmannosamine kinase; GPX, glutathione peroxidase; HB-EGF, heparin-binding EGF-like growth factor; HMO, human milk oligosaccharide; LNnT, lacto-N-neotetraose; MHC, myosin heavy chain; OXPHOS, oxidative phosphorylation; PK, pharmacokinetics; SCFA, short-chain fatty acid; SHIME, Simulator of the Human Intestinal Microbial Ecosystem; STZ, streptozotocin; TNBS, 2,4,6-trinitrobenzene sulfonic acid; ZO-1, zonula occludens-1.

**Table 3:** Adult translational evidence for HMO safety, microbiome responsiveness, systemic availability, and host outcomes.

Study and Design/Population	HMO Exposure and Duration	Main Translational Findings	Critical Boundary	Appraisal
Kim JH et al. [12] Randomized, triple-blind, placebo-controlled trial; 60 healthy adults	6′-SL, 6 g/day Duration: 12 weeks	No serious adverse reactions; gastrointestinal symptoms and clinically relevant laboratory abnormalities were not increased compared with placebo	Designed to assess safety rather than efficacy; underpowered to detect rare events or subgroup effects	RoB 2: Low risk for safety outcomes
Elison E et al. [11] Randomized, double-blind, placebo-controlled multidose trial; 100 healthy adults	2′-FL and/or LNnT, up to 20 g/day Duration: 2 weeks	Generally well tolerated; increased Actinobacteria/*Bifidobacterium* and altered community composition	Short duration, small treatment groups, and no downstream functional endpoint	RoB 2: Some concerns
Carter MM et al. [13] Three-arm randomized placebo-controlled trial; 89 healthy older adults	2′-FL, 1 or 5 g/day Duration: 6 weeks plus a washout period	Transient high-dose bifidogenic response; detection of 2′-FL in plasma and urine; several circulating metabolic and protein markers	The primary Cytokine Response Score was null; incomplete plasma detection and multiple secondary and responder analyses	RoB 2: Some concerns
Vliex LMM et al. [64] Randomized, double-blind, placebo-controlled trial following vancomycin treatment; 37 adults completed the study	2′-FL, 12 g/day Duration: 8 weeks	Early signs of microbiome resilience and lower IL-6 levels at week 8; generally well tolerated	No sustained benefit in overall resilience, diversity, fecal SCFAs, GLP-1, or most metabolic outcomes	RoB 2: Low risk
Kennedy JM et al. [65] Randomized, double-blind, placebo-controlled crossover trial; 38 adults with mild-to-moderate ulcerative colitis	2′-FL, 5 g/day Duration: 28 days per period	Increased *Bifidobacterium*; no signal of serious adverse events	No primary SCCAI clinical response; attrition, per-protocol biological analysis, and exploratory cross-fluid findings	RoB 2: Some concerns
Canfora EE et al. [15] Randomized, double-blind, three-condition crossover trial; lean and overweight/obese men	2′-FL, 12 g, with or without resistant starch Duration: Acute single-day exposure	Increased circulating butyrate in both phenotypes; acetate and FFA responses were phenotype- and treatment-dependent	No exercise test; fecal microbiota and SCFAs did not show clear parallel changes; the source of circulating SCFAs remains unresolved	RoB 2: Some concerns
Jackson PP et al. [66] Four-arm randomized, double-blind, placebo-controlled trial; 96 randomized working adults	2′-FL, 2 g/day, alone or with fructo-oligosaccharides Duration: 4 weeks	Microbiome changes were more consistent with fructo-oligosaccharides or the combination; several mood and stress indicators were reported	Independent effects of 2′-FL were limited; mood and stress were among multiple secondary outcomes	RoB 2: Low risk for the primary microbiome outcome
Ko J et al. [38] Randomized, double-blind, placebo-controlled lifestyle trial; 41 adults who were overweight	2′-FL, 3 g/day Duration: 12 weeks	Comparable weight loss and no significant omnibus treatment interaction for the primary body composition set or aerobic capacity; favorable patterns were mainly observed within groups or in post hoc analyses, with a small within-group decrease in BMC	Diet/activity co-intervention, numerous outcomes, unadjusted Fisher LSD comparisons, and discrepancies between some narrative claims and interaction tests limit the ability to attribute findings	RoB 2: Some concerns
Jacobs JP et al. [67] Open-label, multidose, longitudinal study; 32 healthy adults, 30 completed	Donor-derived complex HMO concentrate, 1.8–18 g/day Duration: 7 days plus follow-up	Dose-related bifidogenic response, community succession, and changes in circulating metabolites and cytokines; no reported clinical safety signals	No concurrent control group, eight participants per dose, complex mixture, short exposure period, and exploratory multi-omics analysis	ROBINS-I: Serious for causal effect estimation; descriptive value retained
Ryan JJ et al. [69] Open-label, single-arm pilot study; 12 adults with IBS or ulcerative colitis, using a batch-fermented formulation	2′-FL-containing multicomponent nutritional formula Duration: 6 weeks	Reported improvements in gastrointestinal quality of life, symptoms, *Bifidobacterium*, *F. prausnitzii*, and fecal SCFAs	The lack of a control group, the very small sample size, subjective outcomes, and a composite intervention preclude attributing the results to 2′-FL	ROBINS-I: Serious limitations for causal effect estimation; exploratory evidence only
Jantscher-Krenn E et al. [56] Prospective longitudinal pilot cohort; 31 pregnant adults	Exposure to endogenous gestational HMO Duration: Gestational weeks 10–35	Sixteen HMOs detected in serum, with patterns related to gestational age and secretor status	Pregnancy-specific endogenous exposure; observational associations cannot establish oral pharmacokinetics or effect	JBI longitudinal cohort appraisal; descriptive evidence on systemic availability
Vazquez E et al. [55] Oral pharmacokinetic study in adult and suckling rats	2′-FL, 6′-SL, or LNnT Duration: Single-dose time course	Plasma detection and urinary excretion supported partial systemic availability; 2′-FL exposure was dose-dependent in suckling rats	Preclinical bridge only; absolute bioavailability, tissue distribution, and effective exposure in adult humans remain unknown	Structured preclinical PK assessment: moderate support, limited translational relevance

Abbreviations: 2′-FL, 2′-fucosyllactose; 6′-SL, 6′-sialyllactose; BMC, bone mineral content; FFA, free fatty acid; GI, gastrointestinal; GLP-1, glucagon-like peptide-1; HMO, human milk oligosaccharide; IBS, irritable bowel syndrome; IL-6, interleukin-6; LNnT, lacto-N-neotetraose; PK, pharmacokinetics; SCCAI, Simple Clinical Colitis Activity Index; SCFA, short-chain fatty acid.

**Table 4:** Falsifiable hypotheses and priority designs for HMO–exercise translation.

Falsifiable Hypothesis	Current Evidence Basis and Testable Prediction	Required Measurements	Failure Criterion	Priority Population and Design
H1. Gut-dependent pathway	Basis: Studies in adults support structure- and host-dependent responses of the microbiome, metabolites, and the intestinal barrier; exercise-related effects on muscle remain primarily in the preclinical stage, and the full chain of human mediation has not yet been elucidated [53,63]. Prediction: Disrupting microbiota function or transferring candidate microbial metabolites will attenuate the muscle or recovery response to a defined HMO.	Verified HMO exposure; metagenomic or functional microbiome profiling; targeted microbial metabolites; intestinal permeability; serial systemic biomarkers; muscle respiration or protein turnover; prespecified recovery or performance outcome.	The functional effect persists despite a validated disruption of the microbiota or the candidate metabolite pathway, or the proposed mediator does not temporally precede and statistically mediate the outcome.	Mechanistic crossover trial in adults, stratified by baseline microbiome, with frequent serial sampling; parallel perturbation or rescue study for causal validation.
H2. Systemic pathway	Basis: Intact HMOs can be detected systemically at low concentrations, and extra-intestinal effects are biologically plausible, but exercise-state PK/PD and direct skeletal-muscle targeting in humans have not been established [12,54,55]. Prediction: Circulating intact HMOs or host-derived products will exhibit structure-specific dose–exposure relationships and temporally aligned tissue responses that persist even after accounting for the gut-dependent pathway.	Targeted plasma and urine HMO assays; stable-isotope tracing where feasible; pharmacokinetic (PK) and pharmacodynamic (PD) studies under exercise conditions; tissue uptake or target-engagement assays; muscle biopsy or validated measures of muscle function; gut-related covariates.	No reproducible systemic exposure, target engagement, or dose–response relationship is observed, or the putative extraintestinal effect disappears after controlling for gut-derived mediators.	Dose-escalation PK/PD study at rest and during standardized exercise, followed by a mechanistic randomized trial assessing target engagement at the tissue level.
H3. Structure-specific effects	Basis: Cross-study signals differ among 2′-FL, 3′-SL, and 6′-SL, but heterogeneity in formulation, dose, model, and exercise prevents causal attribution to glycan structure. Prediction: Under identical exposure and exercise conditions, individual HMOs will produce reproducible, non-equivalent effects on prespecified proximal and functional outcomes.	Chemically verified HMO identity and purity; exposure-matched multi-arm dosing; standardized exercise protocol; shared core outcomes; multiplicity-adjusted head-to-head comparisons; interaction tests.	Exposure-matched head-to-head studies consistently demonstrate equivalent effects with narrow uncertainty, or apparent differences are explained by dose, formulation, population, or model.	Multi-arm, randomized, dose-ranging trial comparing 2′-FL, 3′-SL, and 6′-SL, with a priori equivalence/non-inferiority margins where appropriate.
H4. Temporal Exercise → HMO Regulation	Basis: Acute maternal exercise did not materially alter milk 3′-SL or 6′-SL in one human study, whereas chronic maternal exercise models implicate 3′-SL in offspring metabolic phenotypes [39,40]. Prediction: Longitudinal training or adaptation during pregnancy and lactation—rather than a single acute bout—will produce a reproducible within-person change in selected milk HMOs that reverses or attenuates with detraining.	Repeated milk HMO profiling before and after acute exercise, across training periods, and during washout; standardized lactation stage, diet, and exercise load; plasma and urine exposure; maternal and infant covariates.	No reproducible within-person temporal association is observed across acute, chronic, and washout phases, or changes are fully explained by lactation stage, diet, or other time-varying confounders.	A prospective repeated-measures cohort of physically active lactating participants, with nested standardized acute-exercise and training/detraining phases.
H5. Muscle-adaptation phenotype	Basis: Preclinical studies suggest effects on muscle protein homeostasis, slow-fiber/OXPHOS features, and substrate metabolism, whereas the available human trials have null primary or omnibus treatment tests and only limited exploratory signals [34,36,37]. Prediction: Changes in a prespecified proximal muscle phenotype will precede, mediate, and predict any improvement in recovery or training adaptation; a uniform benefit across all performance domains is not expected.	Muscle protein synthesis/breakdown; fiber phenotype; mitochondrial respiration and OXPHOS; substrate oxidation; validated neuromuscular and endurance outcomes; serial timing; adherence, adverse events, and diet/training control.	Proximal muscle or metabolic changes do not precede or mediate functional effects, fail to replicate, or performance changes occur without a coherent, direction-consistent phenotype.	Adequately powered, preregistered randomized controlled trial (RCT) of training in older adults and trained athletes, with mechanistic substudies and independent replication.

Abbreviations: HMO, human milk oligosaccharide; OXPHOS, oxidative phosphorylation; PK/PD, pharmacokinetics/pharmacodynamics; RCT, randomized controlled trial.

## Data Availability

The original contributions presented in this study are included in the article and the Supplementary Materials. Further inquiries may be directed to the corresponding author.

## Supplementary Materials

The following supporting information can be downloaded at https://www.mdpi.com/article/10.3390/nu18203417/s1. Supplementary File S1: Complete search strategies for all five bibliographic databases; Table S1: Reports not retrieved after documented full-text retrieval attempts; Table S2: Reports excluded after full-text assessment and their primary reasons for exclusion; Table S3: Mapping of the 40 included reports to 38 independent studies; Table S4: Overall evidence matrix across the direct, mechanistic, and adult translational evidence layers; Tables S5A–S5E: Design-specific risk-of-bias and methodological-quality assessments using RoB 2, ROBINS-I, the JBI longitudinal cohort checklist, SYRCLE, and the structured in vitro/ex vivo appraisal framework; Tables S6A–S6C: Source data underlying Figure 2, Figure 3 and Figure 4. Figure 1 and Figure 5 are conceptual syntheses and do not rely on independent numerical datasets.

## Abbreviations

The following abbreviations are used in this manuscript:

2′-FL	2′-Fucosyllactose
3-FL	3-Fucosyllactose
3′-SL	3′-Sialyllactose
5RM	Five-repetition maximum
6′-SL	6′-Sialyllactose
A/I	Association or indirect evidence
ADAM17	A disintegrin and metalloproteinase 17
AKT	Protein kinase B
AKT1	Protein kinase B alpha
AMPK	AMP-activated protein kinase
BMC	Bone mineral content
CI	Confidence interval; controlled intervention in Figure 4
DM	Direct measurement without causal intervention
DSS	Dextran sulfate sodium
EGF	Epidermal growth factor
EGFR	Epidermal growth factor receptor
ERK	Extracellular signal-regulated kinase
FFA	Free fatty acid
FFAR2	Free fatty acid receptor 2
FFAR3	Free fatty acid receptor 3
FMT	Fecal microbiota transplantation
FOS	Fructooligosaccharide
FOXO1	Forkhead box O1
Fuc	Fucose
FUT2	α1,2-Fucosyltransferase 2
FUT3	α1,3/4-Fucosyltransferase 3
Gal	Galactose
GI	Gastrointestinal
Glc	Glucose
GLP-1	Glucagon-like peptide-1
GNE	UDP-N-acetylglucosamine 2-epimerase/N-acetylmannosamine kinase
GOS	Galactooligosaccharide
GPR41	G-protein-coupled receptor 41
GPX	Glutathione peroxidase
GPR43	G protein-coupled receptor 43
HB-EGF	Heparin-binding EGF-like growth factor
HDAC	Histone deacetylase
HFD	High-fat diet
HIIT	High-intensity interval training
HMO	Human milk oligosaccharide
IBS	Irritable bowel syndrome
IL-6	Interleukin-6
JBI	Joanna Briggs Institute
LNnT	Lacto-N-neotetraose
LNT	Lacto-N-tetraose
LPS	Lipopolysaccharide
LSD	Least significant difference
MEDLINE	Medical Literature Analysis and Retrieval System Online
MHC	Myosin heavy chain
MICT	Moderate-intensity continuous training
MuRF1	Muscle RING-finger protein-1
Neu5Ac	N-Acetylneuraminic acid
NF-κB	Nuclear factor kappa B
Nrf2	Nuclear factor erythroid 2-related factor 2
OSF	Open Science Framework
OXPHOS	Oxidative phosphorylation
P/R	Perturbation or rescue evidence
PGC-1α	Peroxisome proliferator-activated receptor gamma coactivator 1-alpha
PI3K	Phosphoinositide 3-kinase
PI3K/AKT	Phosphoinositide 3-kinase/protein kinase B
PICOS	Population, intervention, comparator, outcomes, and study design
PK	Pharmacokinetics
PK/PD	Pharmacokinetics/pharmacodynamics
PRISMA	Preferred Reporting Items for Systematic Reviews and Meta-Analyses
RCT	Randomized controlled trial
REST	Rest condition
RoB 2	Risk of Bias 2
ROBINS-I	Risk Of Bias In Non-randomized Studies of Interventions
SCCAI	Simple Clinical Colitis Activity Index
SCFA	Short-chain fatty acid
SHIME	Simulator of the Human Intestinal Microbial Ecosystem
SIRT1	Sirtuin 1
STZ	Streptozotocin
SWiM	Synthesis Without Meta-analysis
SYRCLE	Systematic Review Centre for Laboratory Animal Experimentation
TNBS	2,4,6-Trinitrobenzene sulfonic acid
UCP1	Uncoupling protein 1
VANT	Ventilatory anaerobic threshold
VO2max	Maximal oxygen uptake
VO_2_	Oxygen uptake
ZO-1	Zonula occludens-1
