# Supplementary material for: Human Milk Oligosaccharides in Exercise Nutrition: Structure-Specific Evidence, Human Efficacy, and Translational Bottlenecks
Source: Nutrients. 2026 Oct 18;18(20):3417. doi: 10.3390/nu18203417 (PMC13708468; doi:10.3390/nu18203417)
Supplement: Supplementary file 1 [file nutrients-18-03417-s001.zip › Supplementary_Material.pdf]

Supplementary Material

This supplementary file accompanies the critical translational review entitled "Human Milk Oligosaccharides in Exercise Nutrition: Structure-Specific Evidence, Human Efficacy, and Translational Bottlenecks". It provides the methods and audit-level data required to reproduce the literature identification, eligibility decisions, study-family linkage, evidence-layer assignment and design-specific appraisal. The review protocol and methodological decisions were registered in the Open Science Framework (OSF; https://doi.org/10.17605/OSF.IO/YVTS8).

Supplementary Methods

Review scope and eligibility framework

The review used a critical translational design with a structured literature search and three non-mutually exclusive evidence layers. Layer A comprised direct evidence linking a human milk oligosaccharide (HMO) exposure, supplementation or exercise-related HMO response to exercise, training, physical performance, fatigue, skeletal muscle or body-composition outcomes. Layer B comprised HMO studies addressing exercise-relevant mechanisms, including skeletal-muscle biology, mitochondrial function, substrate use, energy metabolism or oxidative stress. Layer C comprised adult translational evidence on safety, tolerability, microbiome responses, microbial metabolites, intestinal-barrier outcomes, systemic availability, absorption or pharmacokinetics. Human, animal, ex vivo and in vitro studies were eligible when they informed at least one layer. Reviews were used for evidence navigation but were not included as primary evidence reports.

Information sources and record management

PubMed/MEDLINE, Embase, Web of Science Core Collection, Scopus and SPORTDiscus were searched from inception through 6–7 September 2026 without language, year, human/animal or publication-type restrictions. The executed database syntax is reproduced in Supplementary File S1. Embase Clinical Trials records were managed as a separate registry stream and were not merged with bibliographic records. Database exports were standardized and deduplicated using DOI, PMID and conflict-safe exact normalized-title matching; candidate fuzzy duplicates were adjudicated manually. Backward and forward citation tracking was used for included reports and prespecified sentinel studies.

Study selection and report retrieval

After deduplication, 2,511 records entered title/abstract screening. Two reviewers (CQ and SG) independently verified retained and uncertain records and confirmed all full-text eligibility decisions; disagreements were resolved by discussion. Of 311 reports sought for retrieval, 124 could not be obtained after public-source and manual retrieval attempts. The remaining 187 reports were assessed in full text; 147 were excluded with one primary reason and 40 reports were included. Reports not retrieved and full-text exclusions are listed separately in Supplementary Tables S1 and S2. Computational tools were used for record normalization, prioritization and extraction support, but no automated decision determined final eligibility.

Study-family linkage and data extraction

Reports were linked before synthesis using cohort, protocol, sample, intervention, comparator, setting and outcome fingerprints. Complementary reports retained their non-overlapping information but contributed only once to the count of independent studies. Forty included reports represented 38 studies. Extracted fields included population or model, HMO structure and exposure, dose, route and duration, exercise context, comparator, outcome domain, effect direction, mechanistic findings, study limitations and translation distance. Layer assignment was non-exclusive. No pooled effect estimate was calculated because the evidence was heterogeneous in design, model, exposure, exercise paradigm and outcome definition.

Risk-of-bias and study-quality appraisal

Appraisal was matched to study design. Critical results from randomized parallel and crossover trials were assessed using RoB 2; non-randomized interventions were assessed using ROBINS-I against an explicit target trial; the prospective longitudinal exposure study was evaluated descriptively using the JBI cohort framework; animal studies were assessed across the ten SYRCLE domains; and in vitro or ex vivo studies were evaluated with a structured mechanism checklist. RoB 2 and ROBINS-I judgements are result-specific. SYRCLE results are reported only at domain level; no unsupported overall animal-study score was assigned. Reporting insufficiency was coded as Unclear rather than High risk unless evidence supported a high-risk judgement.

Supplementary Tables

Supplementary Table S1. Reports sought but not retrieved

Report title	Year	DOI	Candidate layer	Retrieval status
Unlocking the power of human milk and infant feeding: Understanding how nutrition and early microbiota interaction shapes health programming.	2025	10.1016/j.chom.2025.05.014	C	No full text obtained after public and manual retrieval attempts.
The relationship between human milk, a functional nutrient, and microbiota.	2023	10.1080/10408398.2021.2008301	C	No full text obtained after public and manual retrieval attempts.
In vitro fermentation of human milk oligosaccharides by individual Bifidobacterium longum-dominant infant fecal inocula.	2022	10.1016/j.carbpol.2022.119322	B	No full text obtained after public and manual retrieval attempts.
Human milk oligosaccharides, infant growth, and adiposity over the first 4 months of lactation.	2021	10.1038/s41390-020-01328-y	A	No full text obtained after public and manual retrieval attempts.
Lactodifucotetraose, a human milk oligosaccharide, attenuates platelet function and inflammatory cytokine release.	2016	10.1007/s11239-015-1331-2	C	No full text obtained after public and manual retrieval attempts.
Human milk oligosaccharide supplementation in irritable bowel syndrome patients: A parallel, randomized, double-blind, placebo-controlled study.	2020	10.1111/nmo.13920	C	No full text obtained after public and manual retrieval attempts.
Human milk composition and infant growth.	2018	10.1097/mco.0000000000000466	A	No full text obtained after public and manual retrieval attempts.
Effect of supplementation with select human milk oligosaccharides on artificially reared newborn rats.	2022	10.1017/s0007114521005146	A	No full text obtained after public and manual retrieval attempts.
Select human milk oligosaccharide supplementation in post-weanling rats affects metabolism and gut microbiota into adulthood.	2023	10.1002/oby.23731	C	No full text obtained after public and manual retrieval attempts.
Cross-feeding between Bifidobacterium infantis and Anaerostipes caccae on lactose and human milk oligosaccharides.	2021	10.3920/bm2020.0005	B	No full text obtained after public and manual retrieval attempts.
Associations of Early Gut Microbiome and Metabolome with Growth and Body Composition of Preterm Infants Within the First 6 Months.	2024	10.1089/bfm.2023.0258	A	No full text obtained after public and manual retrieval attempts.
Time-resolved growth of diverse human-associated Akkermansia on human milk oligosaccharides.	2026	10.1101/2025.07.07.663590	C	Public or publisher link identified, but full text could not be obtained after manual attempts.
Adv Nutr. 2022 Feb 1;13(1):318-327. doi: 10.1093/advances/nmab102.	Not reported	10.1093/advances/nmab102	C	Public or publisher link identified, but full text could not be obtained after manual attempts.
Dosing a synbiotic of human milk oligosaccharides and B. infantis leads to reversible engraftment in healthy adult microbiomes without antibiotics.	2022	10.1016/j.chom.2022.04.001	C	No full text obtained after public and manual retrieval attempts.
Reproducing the bifidogenic effect of human milk in formula-fed infants: why and how?	2005	10.1080/08035320510043501	C	No full text obtained after public and manual retrieval attempts.
Human breast milk oligosaccharides attenuate necrotizing enterocolitis in rats by suppressing mast cell accumulation, DPPI activity and TLR4 expression in ileum tissue, and regulating mitochondrial damage of Caco-2 cells.	2020	10.1016/j.intimp.2020.106881	B	No full text obtained after public and manual retrieval attempts.
Isolation and characterization of Bifidobacterium spp. from breast milk with different human milk oligosaccharides utilization and anti-inflammatory capacity.	2024	10.1016/j.foodres.2024.115092	B	No full text obtained after public and manual retrieval attempts.
Modulation of bacterial translocation in mice mediated through lactose and human milk oligosaccharides.	2011	10.1016/j.anaerobe.2011.09.002	C	No full text obtained after public and manual retrieval attempts.
In vitro fermentation characteristics of select nondigestible oligosaccharides by infant fecal inocula.	2013	10.1021/jf305056f	B	No full text obtained after public and manual retrieval attempts.

Report title	Year	DOI	Candidate layer	Retrieval status
Assessment of the synbiotic properites of human milk oligosaccharides and Bifidobacterium longum subsp. infantis in vitro and in humanised mice.	2017	10.3920/bm2016.0138	B	No full text obtained after public and manual retrieval attempts.
In Vitro Infant Fecal Fermentation Characteristics of Human Milk Oligosaccharides Were Controlled by Initial Microbiota Composition More than Chemical Structure.	2022	10.1002/mnfr.202200098	B	No full text obtained after public and manual retrieval attempts.
The combination of human milk oligosaccharides 3-fucosyllactose and 3'-sialyllactose alleviates LPS-induced damage to intestinal epithelial cells.	2026	10.1039/d5fo05266a	B	No full text obtained after public and manual retrieval attempts.
Bifidobacterium as Keystone Species Driving Microbial Interactions in the Infant Digestive Tract.	2026	10.1146/annurev-food-052924-065321	B	No full text obtained after public and manual retrieval attempts.
Advances and Challenges of 2'-Fucosyllactose: Physiological Function and Biotechnological Production.	2026	10.1021/acs.jafc.5c11962	C	No full text obtained after public and manual retrieval attempts.
Human milk and related oligosaccharides as prebiotics.	2013	10.1016/j.copbio.2013.01.008	C	No full text obtained after public and manual retrieval attempts.
Bosom Buddies: The Symbiotic Relationship Between Infants and Bifidobacterium longum ssp. longum and ssp. infantis. Genetic and Probiotic Features.	2016	10.1146/annurev-food-041715-033151	C	No full text obtained after public and manual retrieval attempts.
In vitro infant fecal fermentation characteristics of bovine milk osteopontin and 2'-fucosyllactose combination.	2025	10.1016/j.foodres.2025.116980	B	No full text obtained after public and manual retrieval attempts.
Impact of 2'-fucosyllactose on adult gut microbiota composition and metabolome based on colonic fermentation and prebiotic quantitative evaluation.	2025	10.1111/1750-3841.70084	C	No full text obtained after public and manual retrieval attempts.
Gellan gum-sialoglycan conjugates: a mucin mimic for alleviating inflammation in Caco-2 cells and modulating gut microbiota in the elderly.	2025	10.1016/j.ijbiomac.2025.143478	C	No full text obtained after public and manual retrieval attempts.
Precision modulation of dysbiotic adult microbiomes with a human-milk-derived synbiotic reshapes gut microbial composition and metabolites.	2023	10.1016/j.chom.2023.08.004	B/C	No full text obtained after public and manual retrieval attempts.
Impact of non-digestible carbohydrates and prebiotics on immunity, infections, inflammation and vaccine responses: a systematic review of evidence in healthy humans and a discussion of mechanistic proposals.	2026	10.1080/10408398.2025.2514700	C	No full text obtained after public and manual retrieval attempts.
Bifidobacterium infantis and 2'-fucosyllactose supplementation in early life may have potential long-term benefits on gut microbiota, intestinal development, and immune function in mice.	2023	10.3168/jds.2023-23367	C	No full text obtained after public and manual retrieval attempts.
Validation of collection and anaerobic fermentation techniques for measuring prebiotic impact on gut microbiota.	2024	10.1016/j.phrs.2024.107169	C	No full text obtained after public and manual retrieval attempts.
High-Level Productivity of Lacto-N-neotetraose in Escherichia coli by Systematic Metabolic Engineering.	2023	10.1021/acs.jafc.2c08772	B	No full text obtained after public and manual retrieval attempts.
A pilot trial for efficacy confirmation of 6'-sialyllactose supplementation in GNE myopathy: Randomized, placebo-controlled trial.	2025	10.1016/j.ymgme.2024.108614	A	No full text obtained after public and manual retrieval attempts.
Evolution of the gut microbiome in infancy: recent advances.	2026	10.1097/mco.0000000000001209	C	No full text obtained after public and manual retrieval attempts.
Prebiotic oligosaccharides in early life alter gut microbiome development in male mice while supporting influenza vaccination responses.	2019	10.3920/bm2018.0098	C	No full text obtained after public and manual retrieval attempts.
What Does Healthy Microbiome Development Look Like? State of the Art and Beyond.	2024	10.1159/000540144	C	No full text obtained after public and manual retrieval attempts.
Effect of 2'-fucosyllactose and hydrolysis degree on oxidation, Maillard reaction, and physical stability of whey protein hydrolysate emulsions.	2026	10.1016/j.foodres.2026.120211	B	No full text obtained after public and manual retrieval attempts.
Maternal-Infant Gut Microbiota Transmission and the Early Origins of Metabolic Liver Diseases: Mechanisms and Interventional Opportunities.	2026	10.1093/nutrit/nuag074	C	No full text obtained after public and manual retrieval attempts.
The complex-type oligosaccharide binding lectin Datura stramonium agglutinin detects type II A muscle fibres in the branchial biceps from man and cat.	1997	10.1023/a:1018624715326	B	No full text obtained after public and manual retrieval attempts.
Localized agglutinin staining in muscle capillaries from normal and very old atrophic human muscle using winged bean (Psophocarpus tetragonolobus) lectin.	1997	10.1007/s004180050086	B	No full text obtained after public and manual retrieval attempts.
Relationship Between Oligosaccharides and Glycoconjugates Content in Human Milk and the Development of the Gut Barrier.	2019	10.1111/1541-4337.12400	C	No full text obtained after public and manual retrieval attempts.
Gut Microbiota Development: Influence of Diet from Infancy to Toddlerhood.	2021	10.1159/000517912	C	No full text obtained after public and manual retrieval attempts.
Lacto-N-Triose II Alleviates DSS-Induced Colitis via Gut Microbiota and Metabolites Associated with the PI3K-Akt Pathway.	2026	10.1021/acs.jafc.5c11151	B	No full text obtained after public and manual retrieval attempts.
Butyrate-producing colonic clostridia: picky glycan utilization specialists.	2023	10.1042/ebc20220125	C	No full text obtained after public and manual retrieval attempts.
Effect of Bifidobacterium crudilactis and 3'-sialyllactose on the toddler microbiota using the SHIME® model.	2020	10.1016/j.foodres.2020.109755	C	No full text obtained after public and manual retrieval attempts.
Multiomics Analysis Reveals the Therapeutic Effect of GRAS Yeast-Derived 2'-Fucosyllactose in DSS-Induced Colitis.	2025	10.1021/acs.jafc.5c08620	B	No full text obtained after public and manual retrieval attempts.
Human milk oligosaccharide 2'-fucosyllactose attenuates oxidative stress-induced intestinal barrier injury in mice via regulating microbial tryptophan metabolism.	2026	10.1039/d5fo05555e	B	No full text obtained after public and manual retrieval attempts.
Increased Intake of Foods Providing Alpha-Linolenic Acid Modifies Nutritional and Nonnutritional Characteristics of Breast Milk: A Randomized Clinical Trial.	2026	10.1016/j.tjnut.2026.101787	C	No full text obtained after public and manual retrieval attempts.
Inhibitory effects of 6'-sialyllactose on angiotensin II-induced proliferation, migration, and osteogenic switching in vascular smooth muscle cells.	2022	10.1007/s12272-022-01404-3	B	No full text obtained after public and manual retrieval attempts.
Chicory inulin enhances fermentation of 2'-fucosyllactose by infant fecal microbiota and differentially influences immature dendritic cell and T-cell cytokine responses under normal and Th2-polarizing conditions.	2021	10.1039/d1fo00893e	B	No full text obtained after public and manual retrieval attempts.
3-Fucosyllactose and 3'-sialyllactose combined with Lacticaseibacillus rhamnosus 1.0320 alleviate necrotizing enterocolitis by modulating the gut microbiota and regulating NF-κB/Nrf2 pathways.	2026	10.1039/d5fo05215g	B	No full text obtained after public and manual retrieval attempts.
2'-Fucosyllactose (2'-FL) alleviates choline-deficient fat diet-induced nonalcoholic steatohepatitis (NASH) by remodeling intestinal flora.	2025	10.1007/s10068-025-02034-3	B	Public or publisher link identified, but full text could not be obtained after manual attempts.
2'-Fucosyllactose promotes Lactobacillus rhamnosus KLDS 8001 to repair LPS-induced damage in Caco-2 cells.	2022	10.1111/jfbc.14059	B	No full text obtained after public and manual retrieval attempts.
Modeled structural basis for the recognition of α2-3-sialyllactose by soluble Klotho.	2017	10.1096/fj.201700043r	B	Public or publisher link identified, but full text could not be obtained after manual attempts.
Recognition of early and late stages of bladder cancer using metabolites and machine learning.	2019	10.1007/s11306-019-1555-9	A/B	No full text obtained after public and manual retrieval attempts.
Constitutive repressor activity of CD33 on human monocytes requires sialic acid recognition and phosphoinositide 3-kinase-mediated intracellular signaling.	2005	10.1002/eji.200425273	B	No full text obtained after public and manual retrieval attempts.
Potential small effector molecules restoring cellular defects due to sialic acid biosynthetic enzyme deficiency: Pathological relevance to GNE myopathy.	2024	10.1016/j.bcp.2024.116199	A/B	No full text obtained after public and manual retrieval attempts.
Continuous assay of neuraminidase activity by a bienzyme system immobilized in an artificial membrane.	1987	10.1002/bit.260300204	B	No full text obtained after public and manual retrieval attempts.
N-Acetylneuraminic acid and 3'-sialyllactose supplementation unlock shared and unique atheroprotective mechanisms through the gut-liver-coronary axis in hypercholesterolemic LDLR(-/-) mice.	2026	10.1039/d5fo04031k	B	No full text obtained after public and manual retrieval attempts.
Inhibitory effect of gut microbiota-fermented trisaccharide GuFGa (Glc-Fuc-Gal) on Campylobacter jejuni: insights from transcriptomic and metabolomic analyses.	2026	10.1007/s42995-026-00394-1	B	Public or publisher link identified, but full text could not be obtained after manual attempts.

Report title	Year	DOI	Candidate layer	Retrieval status
[Animal model of distal myopathy with rimmed vacuoles/hereditary inclusion body myopathy and preclinical trial with sugar compounds].	2010	Not reported	A	No full text obtained after public and manual retrieval attempts.
Effects of 2'-fucosyllactose on the composition and metabolic activity of intestinal microbiota from piglets after in vitro fermentation.	2024	10.1002/jsfa.13037	B	No full text obtained after public and manual retrieval attempts.
2'-Fucosyllactose evokes colonization of Alloprevotella and alleviates renal injury in hyperuricemia mice.	2025	10.1039/d5fo00580a	B	No full text obtained after public and manual retrieval attempts.
Galactooligosaccharide or 2'-Fucosyllactose Modulates Gut Microbiota and Inhibits LPS/TLR4/NF-κB Signaling Pathway to Prevent DSS-Induced Colitis Aggravated by a High-Fructose Diet in Mice.	2023	10.1021/acs.jafc.2c08814	B	No full text obtained after public and manual retrieval attempts.
Bull sperm binding to oviductal epithelium is mediated by a Ca2+-dependent lectin on sperm that recognizes Lewis-a trisaccharide.	1998	10.1095/biolreprod59.1.39	B	No full text obtained after public and manual retrieval attempts.
Synergistic effect of 2'-fucosyllactose and osteopontin on intestinal mucosal immunity injury.	2025	10.1039/d4fo05287k	B	No full text obtained after public and manual retrieval attempts.
Comparative effect of sialic acid and 3'-sialyllactose on fecal microbiota fermentation and prebiotic activity in ETEC-challenged IPEC-J2 cells.	2025	10.1002/jsfa.14372	B	No full text obtained after public and manual retrieval attempts.
Protective effect of sialyllactose on the intestinal epithelium in weaned pigs upon enterotoxigenic Escherichia coli challenge.	2022	10.1039/d2fo02066a	B	No full text obtained after public and manual retrieval attempts.
Supplementing sialyllactose to colostrum replacer improved intestinal health and blood immunity by affecting the abundance of intestinal bacteria in dairy calves.	2025	10.3168/jds.2024-25899	B	No full text obtained after public and manual retrieval attempts.
Sialidase of rat hepatomas: qualitative and quantitative comparison with rat liver sialidase.	1984	Not reported	B	No full text obtained after public and manual retrieval attempts.
Molecular cloning and biochemical characterization of medaka (Oryzias latipes) lysosomal neu4 sialidase.	2014	10.1007/s10695-014-9940-9	B	No full text obtained after public and manual retrieval attempts.
Radial basis function neural network optimization algorithm based on dynamic inertial weight particle swarm optimization for separating overlapping peaks in ion mobility spectrometry.	2024	10.1002/rcm.9700	A	Public or publisher link identified, but full text could not be obtained after manual attempts.
Solubilization and affinity chromatography of a sialidase from human liver.	1982	10.1515/bchm2.1982.363.2.1097	B	No full text obtained after public and manual retrieval attempts.
Context-dependent functionality of 2′-fucosyllactose with Bifidobacterium breve M-16 V and short-chain galacto−/long-chain fructo-oligosaccharides in infant gut fermentation	2026	10.1016/j.foodres.2026.120387	C	No full text obtained after public and manual retrieval attempts.
The Gut–Liver Axis in Preterm Neonates: Implications of Dysbiosis-induced Liver Injury	2026	10.1177/09732179261440740	B	No full text obtained after public and manual retrieval attempts.
Preterm nutrition and brain development	2025	10.1136/bmjnph-2023-000749	B	No full text obtained after public and manual retrieval attempts.
Bifidobacteria-mediated immune system imprinting early in life	2021	10.1016/j.cell.2021.05.030	B	No full text obtained after public and manual retrieval attempts.
Human milk oligosaccharides and gut microbiota	2020	10.36290/ped.2020.071	C	No full text obtained after public and manual retrieval attempts.
Molecular characterization of pig UDP-N-acetylglucosamine-2-epimerase/N- acetylmannosamine kinase (Gne) gene: Effect of dietary sialic acid supplementation on gene expression in piglets	2007	Not reported	A	No full text obtained after public and manual retrieval attempts.
Cloning of human liver cytosolic sialidase from genomic DNA using splicing by overlap extension and its characterization	2005	Not reported	A	No full text obtained after public and manual retrieval attempts.
Deciphering the differential effects of human milk oligosaccharides (HMOs) on infant and adult microbiota: insights from a novel “gut microbe-host interaction” model	2026	10.1016/j.jff.2026.107470	B/C	No full text obtained after public and manual retrieval attempts.
Human milk oligosaccharides ameliorate intestinal barrier in aging honey bee by regulating gut microbiota	2025	10.1016/j.fbio.2025.105954	B	No full text obtained after public and manual retrieval attempts.
Human milk oligosaccharides improve intestinal barrier integrity and immune homeostasis through the microbial metabolite butyrate	2026	10.1016/j.fbio.2026.108309	B	No full text obtained after public and manual retrieval attempts.
Lacto-N-neotetraose alleviates obesity in mice by modulating the gut microbiota and short-chain fatty acid metabolism	2026	10.1016/j.idairyj.2025.106419	B	No full text obtained after public and manual retrieval attempts.
2′-Fucosyllactose alters the composition and activity of gut microbiota from formula-fed infants receiving complementary feeding in a validated intestinal model	2019	10.1016/j.jff.2019.103484	B	No full text obtained after public and manual retrieval attempts.
2′-Fucosyllactose Alleviates Hair Loss in Testosterone-Induced Androgenic Alopecia Mice	2025	10.1155/jfbc/5625208	B	No full text obtained after public and manual retrieval attempts.
Intake of neutral core human milk oligosaccharides (HMOs) during early life improves long-term bone quality	2025	10.1016/j.jnutbio.2025.109968	A	Public or publisher link identified, but full text could not be obtained after manual attempts.
3-Fucosyllactose alleviates DSS-induced mouse colitis through modulation of the PI3K-Akt signaling pathway: An integrated multi-omics analysis	2025	10.1016/j.jff.2025.106789	B	No full text obtained after public and manual retrieval attempts.
Integrated transporter engineering and metabolic flux redistribution for efficient production of 3-fucosyllactose in Escherichia coli	2026	10.1016/j.fbio.2026.109441	A	No full text obtained after public and manual retrieval attempts.
Comparative analysis of two Bifidobacterium longum subsp. infantis strains: genomic and phenotypic insights into 2′-fucosyllactose utilization	2025	10.1016/j.fbio.2025.107978	B	No full text obtained after public and manual retrieval attempts.
Relative fermentation of oligosaccharides from human milk and plants by gut microbes	2017	10.1007/s00217-016-2730-0	C	No full text obtained after public and manual retrieval attempts.
Maillard reaction impairs the prebiotic properties of 2′-fucosyllactose after conjugation with α-lactalbumin	2026	10.1016/j.idairyj.2025.106517	A	No full text obtained after public and manual retrieval attempts.
In vitro digestion and fermentation of sialyllactoses by infant gut microflora	2016	10.1016/j.jff.2015.12.002	B	No full text obtained after public and manual retrieval attempts.
The Biological Activity of Human Milk Oligosaccharides	2025	10.3724/j.pibb.2025.0070	B	No full text obtained after public and manual retrieval attempts.
Beneficial actions of breast milk Why and how?	2015	Not reported	C	No full text obtained after public and manual retrieval attempts.
The prebiotics 2′-fucosyllactose prevent high-fat diet induced obesity via the promotion of thermogenesis and modulation of gut microbiota	2024	10.1016/j.jff.2024.106287	B	No full text obtained after public and manual retrieval attempts.
Amelioration of inflammation in D-galactose-induced aging C57BL/6J mice via sialoglycan-enriched soybean protein isolate	2025	10.1016/j.fbio.2025.106964	B	No full text obtained after public and manual retrieval attempts.
In vitro faecal fermentation metabolites of 2'-fucosyllactose protect against intestinal epithelial injury: Infant enterotype effects	2024	10.1016/j.idairyj.2023.105860	B	No full text obtained after public and manual retrieval attempts.
Integrated metabolomic and transcriptomic analysis reveals the molecular mechanism underlying efficient 2'-fucosyllactose utilization by Bifidobacterium longum subsp. infantis	2026	10.1016/j.fbio.2026.109253	B	No full text obtained after public and manual retrieval attempts.
Oligosaccharide-lactoferrin shell-crosslinked particles for selective targeting of proteins to probiotic bacteria in the colon	2021	10.1016/j.foodhyd.2021.106973	A	No full text obtained after public and manual retrieval attempts.
S-layer protein preparations from Bifidobacterium bifidum DNG6 cultured with 2′-fucosyllactose attenuate LPS-induced inflammatory responses in NCM460 cells	2026	10.1016/j.idairyj.2026.106802	A/B	No full text obtained after public and manual retrieval attempts.
Improvement of major depressive disorder through microorganism-host interaction mechanism of 2'-fucosyllactose combined with Bifidobacterium	2025	10.13381/j.cnki.cjm.202512009	B	No full text obtained after public and manual retrieval attempts.
FUNCTIONS AND INDIVIDUAL MECHANISMS OF INFLUENCE OF BIFIDOBACTERIA ON THE STATE OF HEALTH OF INFANTS (LITERARY REVIEW)	2025	10.24061/2413-4260.xv.1.55.2025.23	C	No full text obtained after public and manual retrieval attempts.
Identification and characterization of a novel α2,3-sialyltransferase from Basilea psittacipulmonis for 3′-sialyllactose biosynthesis	2026	10.1016/j.ijbiomac.2026.151981	A	No full text obtained after public and manual retrieval attempts.

Report title	Year	DOI	Candidate layer	Retrieval status
Impact of dietary sialoglycopeptides glycosylation on pathogenic microorganisms and gut microbiota in elderly individuals	2025	10.13995/j.cnki.11-1802/ts.042076	C	No full text obtained after public and manual retrieval attempts.
Current State of infant-type Bifidobacteria in probiotic functional research	2025	10.20043/j.cnki.mpm.202503158	C	No full text obtained after public and manual retrieval attempts.
Carbohydrate Nutrition	2025	10.1016/c2022-0-01619-6	A	No full text obtained after public and manual retrieval attempts.
Human Milk Oligosaccharides Improve the Intestinal Barrier in D-Galactose-induced Aging Mice	2026	10.13386/j.issn1002-0306.2025010265	B	No full text obtained after public and manual retrieval attempts.
How Nursing Mothers Protect Their Babies with Bifidobacteria	2022	10.1002/9781119762621.ch2	B	No full text obtained after public and manual retrieval attempts.
Effects of Human Milk Oligosaccharides on Intestinal Flora and Cognitive Function of Infants	2024	10.16429/j.1009-7848.2024.08.044	C	No full text obtained after public and manual retrieval attempts.
Globesity and Increasing Noncommunicable Diseases	2024	10.1159/000534995	A	No full text obtained after public and manual retrieval attempts.
Early diet and the infant gut microbiome: how breastfeeding and solid foods shape the microbiome	2023	10.3920/978-90-8686-839-1_5	C	No full text obtained after public and manual retrieval attempts.
FUNCTIONS AND INDIVIDUAL MECHANISMS OF INFLUENCE OF BIFIDOBACTERIA ON THE STATE OF HEALTH OF INFANTS (LITERARY REVIEW)	2024	10.24061/2413-4260.xiv.4.54.2024.26	C	No full text obtained after public and manual retrieval attempts.
Human milk oligosaccharides: basic mechanisms of functional effects and potential for disease prevention in children	2024	10.20953/1727-5784-2024-3-44-58	C	No full text obtained after public and manual retrieval attempts.
Human Milk Microbes-Summary and Research Gaps	2017	10.1016/b978-0-12-802725-7.00017-8	A	No full text obtained after public and manual retrieval attempts.
Novel prebiotics and next-generation probiotics: opportunities and challenges	2021	10.1016/b978-0-12-819815-5.00055-0	B	No full text obtained after public and manual retrieval attempts.
Breast milk: Effects of breast milk oligosaccharides on the growth and development of infants (Part 3)	2019	10.4067/s0717-75182019000500644	C	No full text obtained after public and manual retrieval attempts.
Erratum regarding missing ethics statements in previously published articles (Journal of Functional Foods (2019) 63, (S175646461930516X), (10.1016/j.jff.2019.103592))	2020	10.1016/j.jff.2020.103913	B/C	No full text obtained after public and manual retrieval attempts.
Human Milk Oligosaccharides as Modulators of Intestinal and Systemic Immunity	2017	10.1016/b978-0-12-802725-7.00008-7	C	No full text obtained after public and manual retrieval attempts.
Milk oligosaccharides	2011	Not reported	C	No full text obtained after public and manual retrieval attempts.
Molecular pathogenesis and therapeutic strategy in GNE myopathy	2016	10.1007/978-4-431-55678-7_4	B	No full text obtained after public and manual retrieval attempts.
Oligosaccharides in milk: Their benefits and future utilization	2010	10.5398/medpet.2010.33.3.189	C	No full text obtained after public and manual retrieval attempts.

Note. These reports were not subjected to full-text eligibility assessment and are therefore not counted among full-text exclusions. Public-source and manual retrieval attempts were completed; n = 124.

Supplementary Table S2. Reports excluded after full-text assessment and the single primary reason for exclusion

Report title	DOI	Primary reason for exclusion
Human milk oligosaccharide-sharing by a consortium of infant derived Bifidobacterium species.	10.1038/s41598-022-07904-y	Infant/lactation-only evidence without an exercise-relevant mechanism
Sialylated human milk oligosaccharides program cognitive development through a non-genomic transmission mode.	10.1038/s41380-021-01054-9	No sufficient exercise, skeletal-muscle or exercise-metabolism relevance
In vitro competition with Bifidobacterium strains impairs potentially pathogenic growth of Clostridium perfringens on 2'-fucosyllactose.	10.1080/19490976.2025.2478306	No sufficient exercise, skeletal-muscle or exercise-metabolism relevance
2'-Fucosyllactose inhibits human norovirus replication in human intestinal enteroids.	10.1101/2024.05.30.596597	No sufficient exercise, skeletal-muscle or exercise-metabolism relevance
Uptake of fucosylated type I human milk oligosaccharide blocks by Bifidobacterium longum subsp. infantis.	10.1128/mbio.00368-25	Infant/lactation-only evidence without an exercise-relevant mechanism
Human Milk Oligosaccharides, Growth, and Body Composition in Very Preterm Infants.	10.3390/nu16081200	Infant/lactation-only evidence without an exercise-relevant mechanism
Human Milk Oligosaccharides and Bacterial Profile Modulate Infant Body Composition during Exclusive Breastfeeding.	10.3390/ijms23052865	Infant/lactation-only evidence without an exercise-relevant mechanism
Bifidobacterium species associated with breastfeeding produce aromatic lactic acids in the infant gut.	10.1038/s41564-021-00970-4	Infant/lactation-only evidence without an exercise-relevant mechanism
Selective human milk oligosaccharide utilization by members of the Bifidobacterium pseudocatenulatum taxon.	10.1128/aem.00648-24	No eligible adult translational evidence
An Extensively Hydrolyzed Formula Supplemented with Two Human Milk Oligosaccharides Modifies the Fecal Microbiome and Metabolome in Infants with Cow's Milk Protein Allergy.	10.3390/ijms241411422	Infant/lactation-only evidence without an exercise-relevant mechanism
Supplementation with short-chain fatty acids and a prebiotic improves clinical outcome in Parkinson's disease: a randomized double-blind prospective study.	10.1038/s41598-025-29692-x	Exposure was not a human milk oligosaccharide
Early Life Exposure to Human Milk Oligosaccharides Reduces Allergic Response in a Murine Asthma Model.	10.1155/2023/9603576	Infant/lactation-only evidence without an exercise-relevant mechanism
Simulation and modeling of dietary changes in the infant gut microbiome.	10.1093/femsec/fiy140	Infant/lactation-only evidence without an exercise-relevant mechanism
Genome-scale metabolic modeling of the human milk oligosaccharide utilization by Bifidobacterium longum subsp. infantis.	10.1128/msystems.00715-23	Infant/lactation-only evidence without an exercise-relevant mechanism
Characterization of Bifidobacterium kashiwanohense that utilizes both milk- and plant-derived oligosaccharides.	10.1080/19490976.2023.2207455	No eligible adult translational evidence
Linking Human Milk Oligosaccharides, Infant Fecal Community Types, and Later Risk To Require Antibiotics.	10.1128/mbio.03196-19	Infant/lactation-only evidence without an exercise-relevant mechanism
Human milk and mucosa-associated disaccharides impact on cultured infant fecal microbiota.	10.1038/s41598-020-68718-4	Infant/lactation-only evidence without an exercise-relevant mechanism
In silico analysis of the human milk oligosaccharide glycome reveals key enzymes of their biosynthesis.	10.1038/s41598-022-14260-4	HMO biosynthesis/production only; no eligible biological outcome
Human milk oligosaccharide 2'-fucosyllactose promotes melanin degradation via the autophagic AMPK-ULK1 signaling axis.	10.1038/s41598-022-17896-4	No sufficient exercise, skeletal-muscle or exercise-metabolism relevance
Infant gut microbiome is enriched with Bifidobacterium longum ssp. infantis in Old Order Mennonites with traditional farming lifestyle.	10.1111/all.14877	Infant/lactation-only evidence without an exercise-relevant mechanism
Fecal metatranscriptomics and glycomics suggest that bovine milk oligosaccharides are fully utilized by healthy adults.	10.1016/j.jnutbio.2020.108340	No sufficient exercise, skeletal-muscle or exercise-metabolism relevance
Human Milk Oligosaccharides versus Streptococcus: How a Human-Made Natural Product Protects Us from Pathogens.	10.1128/msphere.00049-22	Review, commentary or background source without eligible original evidence
Determination of Seven Human Milk Oligosaccharides (HMOs) in Infant Formula and Adult Nutritionals: First Action 2022.07.	10.1093/jaoacint/qsae001	Infant/lactation-only evidence without an exercise-relevant mechanism
Breastfeeding and the major fermentation metabolite lactate determine occurrence of Peptostreptococcaceae in infant feces.	10.1080/19490976.2023.2241209	Infant/lactation-only evidence without an exercise-relevant mechanism

Report title	DOI	Primary reason for exclusion
Factors affecting early-life intestinal microbiota development.	10.1016/j.nut.2020.110812	Review, commentary or background source without eligible original evidence
Longitudinal change of selected human milk oligosaccharides and association to infants' growth, an observatory, single center, longitudinal cohort study.	10.1371/journal.pone.0171814	Infant/lactation-only evidence without an exercise-relevant mechanism
Immunomodulation by Human Milk Oligosaccharides: The Potential Role in Prevention of Allergic Diseases.	10.3389/fimmu.2020.00801	Review, commentary or background source without eligible original evidence
Early-life immune imprinting by a B. infantis-HMO synbiotic shapes infant immune signatures and protects against experimental respiratory disease.	10.1016/j.xcrm.2026.102935	Infant/lactation-only evidence without an exercise-relevant mechanism
Human Milk Oligosaccharide Concentrations and Infant Intakes Are Associated with Maternal Overweight and Obesity and Predict Infant Growth.	10.3390/nu13020446	Infant/lactation-only evidence without an exercise-relevant mechanism
Gestational diabetes, human milk oligosaccharide concentrations, and their links to infant weight gain and the gut microbiome in a United States observational cohort.	10.1016/j.ajcnut.2026.101235	Infant/lactation-only evidence without an exercise-relevant mechanism
Dual human milk oligosaccharide-fibre utilisation is a selection cue for the weaning gut microbiome.	10.1038/s41467-026-73297-5	No eligible adult translational evidence
Age-dependent associations of human milk oligosaccharides with body size and composition up to 4 years of age.	10.1016/j.ajcnut.2023.02.016	Infant/lactation-only evidence without an exercise-relevant mechanism
Human Milk Oligosaccharides Modulate Fecal Microbiota and Are Safe for Use in Children With Overweight: A Randomized Controlled Trial.	10.1097/mpg.0000000000003205	No eligible adult translational evidence
Combining galacto-oligosaccharides and 2'-fucosyllactose alters their fermentation kinetics by infant fecal microbiota and influences AhR-receptor dependent cytokine responses in immature dendritic cells.	10.1039/d2fo00550f	Infant/lactation-only evidence without an exercise-relevant mechanism
Human milk oligosaccharide 2'-fucosyllactose supplementation improves gut barrier function and signaling in the vagal afferent pathway in mice.	10.1039/d1fo00658d	No sufficient exercise, skeletal-muscle or exercise-metabolism relevance
The principal fucosylated oligosaccharides of human milk exhibit prebiotic properties on cultured infant microbiota.	10.1093/glycob/cws138	Infant/lactation-only evidence without an exercise-relevant mechanism
Associations between human milk oligosaccharides and infant body composition in the first 6 mo of life.	10.3945/ajcn.115.115451	Infant/lactation-only evidence without an exercise-relevant mechanism
Human Breast Milk NMR Metabolomic Profile across Specific Geographical Locations and Its Association with the Milk Microbiota.	10.3390/nu10101355	No sufficient exercise, skeletal-muscle or exercise-metabolism relevance
Human Milk Oligosaccharide Supplementation Affects Intestinal Barrier Function and Microbial Composition in the Gastrointestinal Tract of Young Sprague Dawley Rats.	10.3390/nu12051532	No sufficient exercise, skeletal-muscle or exercise-metabolism relevance
HMOs Exert Marked Bifidogenic Effects on Children's Gut Microbiota Ex Vivo, Due to Age-Related Bifidobacterium Species Composition.	10.3390/nu15071701	No eligible adult translational evidence
Utilization of major fucosylated and sialylated human milk oligosaccharides by isolated human gut microbes.	10.1093/glycob/cwt065	No sufficient exercise, skeletal-muscle or exercise-metabolism relevance
Human milk oligosaccharides protect bladder epithelial cells against uropathogenic Escherichia coli invasion and cytotoxicity.	10.1093/infdis/jit464	No sufficient exercise, skeletal-muscle or exercise-metabolism relevance
Infant Formula Supplemented with Biotics: Current Knowledge and Future Perspectives.	10.3390/nu12071952	Review, commentary or background source without eligible original evidence
Human Milk Microbiome and Microbiome-Related Products: Potential Modulators of Infant Growth.	10.3390/nu14235148	Review, commentary or background source without eligible original evidence
Associations between Maternal Nutrition and the Concentrations of Human Milk Oligosaccharides in a Cohort of Healthy Australian Lactating Women.	10.3390/nu15092093	No sufficient exercise, skeletal-muscle or exercise-metabolism relevance
Novel Genes and Metabolite Trends in Bifidobacterium longum subsp. infantis Bi-26 Metabolism of Human Milk Oligosaccharide 2'-fucosyllactose.	10.1038/s41598-019-43780-9	Infant/lactation-only evidence without an exercise-relevant mechanism
"OMICS" in Human Milk: Focus on Biological Effects on Bone Homeostasis.	10.3390/nu16223921	Review, commentary or background source without eligible original evidence
Systemic Availability of Human Milk Oligosaccharides in Infants and Adults: A Narrative Review.	10.1016/j.advnut.2025.100488	Review, commentary or background source without eligible original evidence
Human Milk Oligosaccharide Profiles and Associations with Maternal Nutritional Factors: A Scoping Review.	10.3390/nu13030965	Review, commentary or background source without eligible original evidence
Bifidobacterium bifidum ATCC 15696 and Bifidobacterium breve 24b Metabolic Interaction Based on 2'-O-Fucosyl-Lactose Studied in Steady-State Cultures in a Freter-Style Chemostat.	10.1128/aem.02783-18	No sufficient exercise, skeletal-muscle or exercise-metabolism relevance
Human Milk Oligosaccharides Modulating Inflammation in Infants, Adults, and Older Individuals-From Concepts to Applications.	10.1016/j.advnut.2025.100433	Review, commentary or background source without eligible original evidence
Microbial composition and in vitro fermentation patterns of human milk oligosaccharides and prebiotics differ between formula-fed and sow-reared piglets.	10.3945/jn.111.154427	Infant/lactation-only evidence without an exercise-relevant mechanism
Clinical Studies on the Supplementation of Manufactured Human Milk Oligosaccharides: A Systematic Review.	10.3390/nu15163622	Review, commentary or background source without eligible original evidence
A quantitative and comprehensive method to analyze human milk oligosaccharide structures in the urine and feces of infants.	10.1007/s00216-013-6817-1	Infant/lactation-only evidence without an exercise-relevant mechanism
Bifidobacterium longum subspecies infantis: champion colonizer of the infant gut.	10.1038/pr.2014.156	Review, commentary or background source without eligible original evidence
Longitudinal analysis of human milk oligosaccharides and mucin-2-glycans in infants reveals enrichment of sulfated and glucuronic acid-bearing HMOs.	10.1016/j.jbc.2026.113099	Infant/lactation-only evidence without an exercise-relevant mechanism
Nursing our microbiota: molecular linkages between bifidobacteria and milk oligosaccharides.	10.1016/j.tim.2010.03.008	Review, commentary or background source without eligible original evidence
Human Milk Oligosaccharide LNnT Attenuates Colonic Barrier Dysfunction and Associated Cognitive Impairment via Modulating Sphingolipid Metabolism and Gut Microbiota.	10.3390/molecules31091410	No sufficient exercise, skeletal-muscle or exercise-metabolism relevance
Inefficient Metabolism of the Human Milk Oligosaccharides Lacto-N-tetraose and Lacto-N-neotetraose Shifts Bifidobacterium longum subsp. infantis Physiology.	10.3389/fnut.2018.00046	Infant/lactation-only evidence without an exercise-relevant mechanism
Butyrate Levels in the Transition from an Infant- to an Adult-Like Gut Microbiota Correlate with Bacterial Networks Associated with Eubacterium Rectale and Ruminococcus Gnavus.	10.3390/genes11111245	Infant/lactation-only evidence without an exercise-relevant mechanism
The Protective Effects of 2'-Fucosyllactose against E. Coli O157 Infection Are Mediated by the Regulation of Gut Microbiota and the Inhibition of Pathogen Adhesion.	10.3390/nu12051284	No sufficient exercise, skeletal-muscle or exercise-metabolism relevance
Bifidobacteria and Their Health-Promoting Effects.	10.1128/microbiolspec.bad-0010-2016	Review, commentary or background source without eligible original evidence
Assessment of stool collection and storage conditions for in vitro human gut model studies.	10.1016/j.mimet.2021.106230	No eligible adult translational evidence
Oral supplementation with Bifidobacterium longum ssp. infantis and 2'-fucosyllactose revives gut microbiota perturbation and intestinal and immune developmental delay following early-life antibiotic challenge in BALB/c mice.	10.3168/jds.2024-24912	Infant/lactation-only evidence without an exercise-relevant mechanism
2'-Fucosyllactose ameliorates aging-related osteoporosis by restoring gut microbial and innate immune homeostasis.	10.1016/j.jare.2024.11.017	No sufficient exercise, skeletal-muscle or exercise-metabolism relevance
A bifidobacterial enzyme orchestrates ecology and function of infant gut bacterial community.	10.64898/2026.04.28.718440	Infant/lactation-only evidence without an exercise-relevant mechanism
Biochemical and structural basis of sialic acid utilization by gut microbes.	10.1016/j.jbc.2023.102989	No eligible adult translational evidence
Maternal obesity and the human milk metabolome: associations with infant body composition and postnatal weight gain.	10.1093/ajcn/nqy334	Infant/lactation-only evidence without an exercise-relevant mechanism
Relationships between the Intakes of Human Milk Components and Body Composition of Breastfed Infants: A Systematic Review.	10.3390/nu15102370	Review, commentary or background source without eligible original evidence

Report title	DOI	Primary reason for exclusion
Extensive Study of Breast Milk and Infant Growth: Protocol of the Cambridge Baby Growth and Breastfeeding Study (CBGS-BF).	10.3390/nu13082879	Protocol or registry report without eligible results
Safety of lacto-N-neotetraose as a novel food ingredient pursuant to Regulation (EC) No 258/97.	10.2903/j.efsa.2015.4183	Review, commentary or background source without eligible original evidence
Gastrointestinal Journey of Human Milk Oligosaccharides: From Breastfeeding Origins to Functional Roles in Adults.	10.3390/microorganisms14010029	Review, commentary or background source without eligible original evidence
Statement on the safety of lacto-N-neotetraose and 2'-O-fucosyllactose as novel food ingredients in food supplements for children.	10.2903/j.efsa.2015.4299	Review, commentary or background source without eligible original evidence
Human Milk Oligosaccharide Composition Is Associated With Excessive Weight Gain During Exclusive Breastfeeding-An Explorative Study.	10.3389/fped.2019.00297	Infant/lactation-only evidence without an exercise-relevant mechanism
Comprehensive metabolic profiling of chronic low-grade inflammation among generally healthy individuals.	10.1186/s12916-017-0974-6	Exposure was not a human milk oligosaccharide
Safety of 2'-O-fucosyllactose as a novel food ingredient pursuant to Regulation (EC) No 258/97.	10.2903/j.efsa.2015.4184	Review, commentary or background source without eligible original evidence
Clinical evidence and mechanistic pathways of human milk oligosaccharide supplementation for health benefits: an updated review.	10.3389/fnut.2025.1599678	Review, commentary or background source without eligible original evidence
[Development of therapy for distal myopathy with rimmed vacuoles].	10.5692/clinicalneurol.49.852	Exposure was not a human milk oligosaccharide
Why Are Bifidobacteria Important for Infants?	10.3390/microorganisms10020278	Review, commentary or background source without eligible original evidence
Peracetylated N-acetylmannosamine, a synthetic sugar molecule, efficiently rescues muscle phenotype and biochemical defects in mouse model of sialic acid-deficient myopathy.	10.1074/jbc.m111.297051	Exposure was not a human milk oligosaccharide
Seeding the Future: How Feeding Mode Shapes the Infant Gut Microbiota.	10.3390/microorganisms14030719	Review, commentary or background source without eligible original evidence
Randomised controlled trial of exercise training during lactation on breast milk composition in breastfeeding people with overweight/obesity: a study protocol for the MILKSHAKE trial.	10.1136/bmjsem-2023-001751	Protocol or registry report without eligible results
The Potential Role of Human Milk Oligosaccharides in Irritable Bowel Syndrome.	10.3390/microorganisms10122338	Review, commentary or background source without eligible original evidence
Bacteroides thetaiotaomicron Fosters the Growth of Butyrate-Producing Anaerostipes caccae in the Presence of Lactose and Total Human Milk Carbohydrates.	10.3390/microorganisms8101513	No sufficient exercise, skeletal-muscle or exercise-metabolism relevance
Tolerability and safety of the intake of bovine milk oligosaccharides extracted from cheese whey in healthy human adults.	10.1017/jns.2017.2	Exposure was not a human milk oligosaccharide
Fucosylated human milk oligosaccharide-utilizing bifidobacteria regulate the gut organic acid profile of infants.	10.12938/bmfh.2023-069	Infant/lactation-only evidence without an exercise-relevant mechanism
Author Correction: Human milk oligosaccharide 2'-fucosyllactose promotes melanin degradation via the autophagic AMPK-ULK1 signaling axis.	10.1038/s41598-022-20541-9	No sufficient exercise, skeletal-muscle or exercise-metabolism relevance
Microbial Glycoside Hydrolases in the First Year of Life: An Analysis Review on Their Presence and Importance in Infant Gut.	10.3389/fmicb.2021.631282	Review, commentary or background source without eligible original evidence
Bifidobacterium longum subsp. infantis ATCC 15697 and Goat Milk Oligosaccharides Show Synergism In Vitro as Anti-Infectives against Campylobacter jejuni.	10.3390/foods9030348	Infant/lactation-only evidence without an exercise-relevant mechanism
Comparative Genomic Analysis of Novel Bifidobacterium longum subsp. longum Strains Reveals Functional Divergence in the Human Gut Microbiota.	10.3390/microorganisms9091906	No eligible adult translational evidence
Bridging preclinical and clinical gut microbiota research using the ex vivo SIFR(®) technology.	10.3389/fmicb.2023.1131662	No eligible adult translational evidence
2'-Fucosyllactose Attenuates Fusobacterium nucleatum Virulence and Modulates the Oral Microbiota.	10.3390/microorganisms14071603	No sufficient exercise, skeletal-muscle or exercise-metabolism relevance
Secretor status-dependent modulation of in vitro immune responses by human milk oligosaccharides.	10.3389/fnut.2026.1791158	No eligible adult translational evidence
In vitro metabolic capacity of carbohydrate degradation by intestinal microbiota of adults and pre-frail elderly.	10.1038/s43705-021-00065-5	No sufficient exercise, skeletal-muscle or exercise-metabolism relevance
Supplementation with 2'-fucosyllactose, a prebiotic human milk oligosaccharide (HMO), in a magnesium-containing medical food reduces chemotherapy-induced mucositis in Wistar rats.	10.3389/fnut.2026.1859479	No sufficient exercise, skeletal-muscle or exercise-metabolism relevance
A Scoping Review of Gut Microbiome and Bifidobacterium Research in Zimbabwe: Implications for Future Studies.	10.2147/phmt.s414766	Review, commentary or background source without eligible original evidence
Trophic Interactions of Infant Bifidobacteria and Eubacterium hallii during L-Fucose and Fucosyllactose Degradation.	10.3389/fmicb.2017.00095	Infant/lactation-only evidence without an exercise-relevant mechanism
In-silico analysis of Bifidobacterium bifidum strain 900791 genome in the context of the B. bifidum pangenome.	10.3389/fcimb.2026.1744409	No sufficient exercise, skeletal-muscle or exercise-metabolism relevance
2-Fucosyllactose Metabolism by Bifidobacteria Promotes Lactobacilli Growth in Co-Culture.	10.3390/microorganisms11112659	No eligible adult translational evidence
Microbiome Composition in Pediatric Populations from Birth to Adolescence: Impact of Diet and Prebiotic and Probiotic Interventions.	10.1007/s10620-020-06092-x	Review, commentary or background source without eligible original evidence
Gut-brain-immune interactions in neonatal hypoxic-ischemic brain injury.	10.1186/s40348-026-00252-1	Review, commentary or background source without eligible original evidence
Probiotic Bifidobacterium bifidum strains desialylate MUC13 and increase intestinal epithelial barrier function.	10.1038/s41598-025-92125-2	No eligible adult translational evidence
Microbiota Supplementation with Bifidobacterium and Lactobacillus Modifies the Preterm Infant Gut Microbiota and Metabolome: An Observational Study.	10.1016/j.xcrm.2020.100077	Infant/lactation-only evidence without an exercise-relevant mechanism
Lossless Compression of Intracortical Extracellular Neural Recordings using Non-Adaptive Huffman Encoding.	10.1109/embc44109.2020.9176352	Ineligible content or study design
2'-Fucosyllactose Increases the Abundance of Blautia in the Presence of Extracellular Fucosidase-Possessing Bacteria.	10.3389/fmicb.2022.913624	No eligible adult translational evidence
Sialylated human milk oligosaccharides prevent intestinal inflammation by inhibiting toll like receptor 4/NLRP3 inflammasome pathway in necrotizing enterocolitis rats.	10.1186/s12986-020-00534-z	No sufficient exercise, skeletal-muscle or exercise-metabolism relevance
Soluble klotho binds monosialoganglioside to regulate membrane microdomains and growth factor signaling.	10.1073/pnas.1620301114	Exposure was not a human milk oligosaccharide
The potential of human milk oligosaccharides in ameliorating traumatic brain injury-induced cognitive impairment in mice.	10.1080/20565623.2026.2622241	No sufficient exercise, skeletal-muscle or exercise-metabolism relevance
Ratio-Dependent Regulation of Butyrate Production by 2'-Fucosyllactose and Fructooligosaccharides in a Bifidobacterium bifidum-Faecalibacterium prausnitzii Co-Culture.	10.3390/microorganisms14081812	No sufficient exercise, skeletal-muscle or exercise-metabolism relevance
2'-Fucosyllactose mitigates cognitive deficits in Alzheimer models: targeting amyloid pathology, oxidative stress, and synaptic plasticity.	10.3389/fphar.2025.1598030	No sufficient exercise, skeletal-muscle or exercise-metabolism relevance
Colonization Resistance in the Infant Gut: The Role of B. infantis in Reducing pH and Preventing Pathogen Growth.	10.3390/ht9020007	Infant/lactation-only evidence without an exercise-relevant mechanism
2'-Fucosyllactose Attenuates Particulate Matter-Induced Inflammation via Inhibition of Hypoxia-Inducible Factor in Keratinocytes.	10.1248/bpb.b18-00963	No sufficient exercise, skeletal-muscle or exercise-metabolism relevance
Sialic acid-based probiotic intervention in lactating mothers improves the neonatal gut microbiota and immune responses by regulating sialylated milk oligosaccharide synthesis via the gut-breast axis.	10.1080/19490976.2024.2334967	Infant/lactation-only evidence without an exercise-relevant mechanism
2'-Fucosyllactose Suppresses Angiogenesis and Alleviates Toxic Effects of 5-Fu in a HCT116 Colon Tumor-Bearing Model.	10.3390/molecules27217255	No sufficient exercise, skeletal-muscle or exercise-metabolism relevance
Key bacterial taxa and metabolic pathways affecting gut short-chain fatty acid profiles in early life.	10.1038/s41396-021-00937-7	Infant/lactation-only evidence without an exercise-relevant mechanism
Fecal microbiome and metabolome of infants fed bovine MFGM supplemented formula or standard formula with breast-fed infants as reference: a randomized controlled trial.	10.1038/s41598-019-47953-4	Infant/lactation-only evidence without an exercise-relevant mechanism

Report title	DOI	Primary reason for exclusion
Human Milk, More Than Simple Nourishment.	10.3390/children8100863	Review, commentary or background source without eligible original evidence
Selected Protective Mechanisms of Human Milk Against Intestinal Protozoal Infections in Infants.	10.3390/cimb47080674	Review, commentary or background source without eligible original evidence
Preterm Infant Fecal Microbiota and Metabolite Profiles Are Modulated in a Probiotic Specific Manner.	10.1097/mpg.0000000000003570	Infant/lactation-only evidence without an exercise-relevant mechanism
Gene-trait matching across the Bifidobacterium longum pan-genome reveals considerable diversity in carbohydrate catabolism among human infant strains.	10.1186/s12864-017-4388-9	No sufficient exercise, skeletal-muscle or exercise-metabolism relevance
Maternal fucosyltransferase 2 status affects the gut bifidobacterial communities of breastfed infants.	10.1186/s40168-015-0071-z	Infant/lactation-only evidence without an exercise-relevant mechanism
Milk sialyllactose influences colitis in mice through selective intestinal bacterial colonization.	10.1084/jem.20101098	No eligible adult translational evidence
Comparison of the Bifidogenic Effects of Goat and Cow Milk-Based Infant Formulas to Human Breast Milk in an in vitro Gut Model for 3-Month-Old Infants.	10.3389/fnut.2020.608495	Infant/lactation-only evidence without an exercise-relevant mechanism
Effects of Antioxidants in Human Milk on Bronchopulmonary Dysplasia Prevention and Treatment: A Review.	10.3389/fnut.2022.924036	Review, commentary or background source without eligible original evidence
Persistence of Supplemented Bifidobacterium longum subsp. infantis EVC001 in Breastfed Infants.	10.1128/msphere.00501-17	Infant/lactation-only evidence without an exercise-relevant mechanism
A Mediterranean diet plan in lactating women with obesity reduces maternal energy intake and modulates human milk composition - a feasibility study.	10.3389/fnut.2024.1303822	No sufficient exercise, skeletal-muscle or exercise-metabolism relevance
Factors influencing gut microbial colonization: A host-microbe-environment interaction perspective.	10.1016/j.crfs.2026.101361	Review, commentary or background source without eligible original evidence
Development and validation of a prediction model for 1-year all-cause rehospitalisation after discharge in patients with heart failure with preserved ejection fraction based on the systemic immune-inflammation index.	10.3389/fcvm.2026.1821564	Ineligible content or study design
African Elephant Milk Short Saccharide and Metabolite Composition and Their Changes over Lactation.	10.3390/ani13030544	Exposure was not a human milk oligosaccharide
A novel function for a glucose analog of blood group H antigen as a mediator of leukocyte-endothelial adhesion via intracellular adhesion molecule 1.	10.1074/jbc.m213052200	Exposure was not a human milk oligosaccharide
Personalized modeling of gut microbiome metabolism throughout the first year of life.	10.1038/s43856-024-00715-4	Infant/lactation-only evidence without an exercise-relevant mechanism
Characterization of the Cultivable Microbiota in Fresh and Stored Mature Human Breast Milk.	10.3389/fmicb.2019.02666	No sufficient exercise, skeletal-muscle or exercise-metabolism relevance
Insights into the utilisation of 1,2-propanediol and interactions with the cell envelope of Clostridium perfringens.	10.1186/s13099-025-00689-1	No sufficient exercise, skeletal-muscle or exercise-metabolism relevance
Endo-1,3(4)-β-Glucanase-Treatment of Oat β-Glucan Enhances Fermentability by Infant Fecal Microbiota, Stimulates Dectin-1 Activation and Attenuates Inflammatory Responses in Immature Dendritic Cells.	10.3390/nu12061660	Exposure was not a human milk oligosaccharide
Evaluation of Risk and Preventive Factors for Necrotizing Enterocolitis in Premature Newborns. A Systematic Review of the Literature.	10.3389/fped.2022.874976	Review, commentary or background source without eligible original evidence
Breastfeeding and the developmental origins of mucosal immunity: how human milk shapes the innate and adaptive mucosal immune systems.	10.1097/mog.0000000000000778	Review, commentary or background source without eligible original evidence
Evaluating the prebiotic effect of oligosaccharides on gut microbiome wellness using in vitro fecal fermentation.	10.1038/s41538-023-00195-1	No eligible adult translational evidence
A mimetic peptide of α2,6-sialyllactose promotes neuritogenesis.	10.4103/1673-5374.270313	Exposure was not a human milk oligosaccharide
Oligofructose alone and in combination with 2'fucosyllactose induces physiologically relevant changes in γ-aminobutyric acid and organic acid production compared to sole 2'fucosyllactose supplementation: an in vitro study.	10.1093/femsec/fiad100	No sufficient exercise, skeletal-muscle or exercise-metabolism relevance
Microbiota-dependent influence of prebiotics on the resilience of infant gut microbiota to amoxicillin/clavulanate perturbation in an in vitro colon model.	10.3389/fmicb.2023.1131953	Infant/lactation-only evidence without an exercise-relevant mechanism
Prebiotics and SCFA Alleviate Parkinson's-Related Constipation: Results from a Randomized Controlled Trial.	10.1007/s40120-026-00972-6	Exposure was not a human milk oligosaccharide
HUMSCs repair CCl_4-induced chronic liver injury in rats via metabolic regulation.	10.1186/s13619-026-00282-0	HMO appeared only as an incidental marker, not an exposure or mechanism
Influence of microbially fermented 2´-fucosyllactose on neuronal-like cell activity in an in vitro co-culture system.	10.3389/fnut.2024.1351433	No sufficient exercise, skeletal-muscle or exercise-metabolism relevance
Hyaluronic Acid-Based Injectable Dual-Network Hydrogel for 6'-Sialyllactose Mimetic Peptide Delivery and Microenvironment Reconstruction in Brachial Plexus Root Avulsion.	10.1002/adhm.71497	Exposure was not a human milk oligosaccharide
Rat-liver lysosomal sialidase. Solubilization, substrate specificity and comparison with the cytosolic sialidase.	10.1111/j.1432-1033.1984.tb08159.x	Exposure was not a human milk oligosaccharide
ABSENCE OF ST3GAL2 AND ST3GAL4 SIALYLTRANSFERASE EXPRESSIONS IN THE NURSE CELL OF TRICHINELLA SPIRALIS	10.15547/bjvm.2020-0006	Exposure was not a human milk oligosaccharide
Biosynthesis of human milk oligosaccharides (HMOs) in glycoengineered human cells	10.1093/glycob/cwag048	HMO biosynthesis/production only; no eligible biological outcome

Note. One primary reason was assigned per report using the frozen eligibility framework; n = 147.

Supplementary Table S3. Included reports, evidence layers and study-family linkage

Study family	Report	Report title	Layer	Relationship	DOI
Family 1	Jacobs et al., 2023	Human milk oligosaccharides modulate the intestinal microbiome of healthy adults.	C	Single Report Study	10.1038/s41598-023-41040-5
Family 2	Estes et al., 2026	Efficacy of Human Milk Oligosaccharide 6'-Sialyllactose Supplementation on Exercise Performance and Training Adaptations.	A/B	Single Report Study	10.3390/nu18111743
Family 3	Park et al., 2024	6'-Sialyllactose Enhances Exercise Performance via Increased Muscle Mass and Strength.	A/B	Companion Report Same Cohort	10.3390/nu16162600
Family 4	Ko et al., 2024	Effects of Human Milk Oligosaccharide 2'-Fucosyllactose Ingestion on Weight Loss and Markers of Health.	A/C	Single Report Study	10.3390/nu16193387
Family 5	Schalich et al., 2024	A human milk oligosaccharide prevents intestinal inflammation in adulthood via modulating gut microbial metabolism.	B	Single Report Study	10.1128/mbio.00298-24
Family 6	Vazquez et al., 2017	Major human milk oligosaccharides are absorbed into the systemic circulation after oral administration in rats.	C	Single Report Study	10.1017/s0007114516004554
Family 3	Park et al., 2024	6'-Sialyllactose Alleviates Muscle Fatigue through Reduced Blood Lactate Level after Treadmill Exercise in Mice.	A/B	Companion Report Same Cohort	10.3390/nu16172957
Family 7	Carter et al., 2025	A human milk oligosaccharide alters the microbiome, circulating hormones, and metabolites in a randomized controlled trial of older adults.	B/C	Single Report Study	10.1016/j.xcrm.2025.102256
Family 8	Jantscher-Krenn et al., 2019	Evidence of human milk oligosaccharides in maternal circulation already during pregnancy: a pilot study.	C	Single Report Study	10.1152/ajpendo.00320.2018
Family 9	Šuligoj et al., 2020	Effects of Human Milk Oligosaccharides on the Adult Gut Microbiota and Barrier Function.	C	Single Report Study	10.3390/nu12092808
Family 10	Kim et al., 2022	Randomized, triple-blind, placebo-controlled study to evaluate the safety of 6'-Sialyllactose in healthy adults.	C	Single Report Study	10.1016/j.yrtph.2021.105110
Family 11	Reens et al., 2024	Tunable control of B. infantis abundance and gut metabolites by co-administration of human milk oligosaccharides.	B/C	Single Report Study	10.1080/19490976.2024.2304160
Family 12	Kennedy et al., 2026	2'-Fucosyllactose modulates gut microbiota and systemic metabolism in mild-to-moderate ulcerative colitis: a randomized crossover trial.	B/C	Single Report Study	10.1093/ecco-jcc/jjag120

Study family	Report	Report title	Layer	Relationship	DOI
Family 13	Ryan et al., 2021	Impact of 2'-Fucosyllactose on Gut Microbiota Composition in Adults with Chronic Gastrointestinal Conditions: Batch Culture Fermentation Model and Pilot Clinical Trial Findings.	C	Single Report Study	10.3390/nu13030938
Family 14	Harris et al., 2020	Exercise-induced 3'-sialyllactose in breast milk is a critical mediator to improve metabolic health and cardiac function in mouse offspring.	A/B	Single Report Study	10.1038/s42255-020-0223-8
Family 15	Firrman et al., 2024	The Bifidogenic Effect of 2'Fucosyllactose Is Driven by Age-Specific Bifidobacterium Species, Demonstrating Age as an Important Factor for Gut Microbiome Targeted Precision Medicine.	C	Single Report Study	10.3390/nu17010151
Family 16	Elison et al., 2016	Oral supplementation of healthy adults with 2'-O-fucosyllactose and lacto-N-neotetraose is well tolerated and shifts the intestinal microbiota.	C	Single Report Study	10.1017/s0007114516003354
Family 17	Vliex et al., 2025	2'-Fucosyllactose supplementation results in a transient improvement in gut microbial resilience after vancomycin use in adults with overweight or obesity: a randomized, double-blind, placebo-controlled intervention.	C	Single Report Study	10.1080/19490976.2025.2580693
Family 18	Li et al., 2025	2'-Fucosyllactose attenuates aging-related metabolic disorders through modulating gut microbiome-T cell axis.	B	Single Report Study	10.1111/acel.14343
Family 19	Go et al., 2024	6'-sialyllactose prevents dexamethasone-induced muscle atrophy by controlling the muscle protein degradation pathway.	A/B	Single Report Study	10.1016/j.bbrc.2024.150892
Family 20	Bajic et al., 2024	HMOs Impact the Gut Microbiome of Children and Adults Starting from Low Predicted Daily Doses.	C	Single Report Study	10.3390/metabo14040239
Family 21	Yonekawa et al., 2014	Sialyllactose ameliorates myopathic phenotypes in symptomatic GNE myopathy model mice.	B	Single Report Study	10.1093/brain/awu210
Family 22	Sato et al., 2024	Sialyllactose Enhances the Short-Chain Fatty Acid Production and Barrier Function of Gut Epithelial Cells via Nonbifidogenic Modification of the Fecal Microbiome in Human Adults.	C	Single Report Study	10.3390/microorganisms12020252
Family 23	Wang et al., 2022	2'-Fucosyllactose Ameliorates Oxidative Stress Damage in d-Galactose-Induced Aging Mice by Regulating Gut Microbiota and AMPK/SIRT1/FOXO1 Pathway.	B	Single Report Study	10.3390/foods11020151
Family 24	Arellano et al., 2023	3'sialyllactose and 6'sialyllactose enhance performance in endurance-type exercise through metabolic adaptation.	A/B	Single Report Study	10.1002/fsn3.3559
Family 25	Sato et al., 2025	Comparative Analysis of Fucosyllactose-Induced Changes in Adult Gut Microbiota and Short-Chain Fatty Acid Production Using the Simulator of Human Intestinal Microbial Ecosystem Model.	C	Single Report Study	10.3746/pnf.2025.30.4.331
Family 26	Phan et al., 2026	Effect of human milk oligosaccharide on the liver-skeletal muscle axis in streptozotocin-induced metabolic stress.	A/B	Single Report Study	10.4196/kjpp.26.014
Family 27	Lemoine et al., 2025	Unaltered 3'-sialyllactose and 6'-sialyllactose concentrations in human milk acutely after endurance exercise: a randomized crossover trial.	A	Single Report Study	10.3389/fnut.2025.1638430
Family 28	Lee et al., 2025	Synbiotic modulation of adult gut microbiome by 2'-fucosyllactose and Bifidobacterium longum subsp. infantis EFEL8008.	C	Single Report Study	10.20517/mrr.2025.35
Family 29	Canfora et al., 2023	2'-fucosyllactose alone or combined with resistant starch increases circulating short-chain fatty acids in lean men and men with prediabetes and obesity.	B/C	Single Report Study	10.3389/fnut.2023.1200645
Family 30	Kaur et al., 2025	2'-Fucosyllactose transactivates EGF receptor in intestinal epithelial cells for prevention of colitis in adulthood.	B	Single Report Study	10.1016/j.isci.2025.114308
Family 31	Baek et al., 2021	3'-Sialyllactose Protects SW1353 Chondrocytic Cells From Interleukin-1β-Induced Oxidative Stress and Inflammation.	B	Single Report Study	10.3389/fphar.2021.609817
Family 32	Jackson et al., 2023	Inulin-type fructans and 2'fucosyllactose alter both microbial composition and appear to alleviate stress-induced mood state in a working population compared to placebo (maltodextrin): the EFFICAD Trial, a randomized, controlled trial.	C	Single Report Study	10.1016/j.ajcnut.2023.08.016
Family 33	Tran-Duc et al., 2025	Sialyllactose Alleviates Streptozotocin-Induced Diabetes through Anti-Inflammatory and Metabolic Modulation.	B	Single Report Study	10.4062/biomolther.2025.083
Family 34	Chen et al., 2023	Effect of 2'-Fucosyllactose on Beige Adipocyte Formation in 3T3-L1 Adipocytes and C3H10T1/2 Cells.	B	Single Report Study	10.3390/foods12224137
Family 35	Yu et al., 2024	Protective effect of 6'-Sialyllactose on LPS-induced macrophage inflammation via regulating Nrf2-mediated oxidative stress and inflammatory signaling pathways.	B	Single Report Study	10.4196/kjpp.2024.28.6.503
Family 36	Lee et al., 2020	2'-fucosyllactose Supplementation Improves Gut-Brain Signaling and Diet-Induced Obese Phenotype and Changes the Gut Microbiota in High Fat-Fed Mice.	B	Single Report Study	10.3390/nu12041003
Family 37	Kim et al., 2024	2′-Fucosyllactose improved muscle health in middle-aged mice performing forced swimming exercise	A/B	Single Report Study	10.1016/j.jff.2024.106567
Family 38	Zhang et al., 2025	3′-Sialyllactose regulates glucose and lipid metabolic disorders via the gut-liver axis in mice fed a high-fat diet	A/B	Companion Report Shared Program	10.26599/fshw.2024.9250185
Family 38	Zhang et al., 2025	The effect of anti-imbalance on glycolipid metabolic homeostasis via the liver–gut axis intervened by 3′-sialyllactose in obese mice	B	Companion Report Shared Program	10.1002/fft2.499

Note. Forty included reports represented 38 independent studies. Two linked report pairs were retained as separate reports but counted once at study level. A, direct evidence; B, mechanistic support; C, adult translation.

Supplementary Table S4. Overall A/B/C evidence matrix for the 40 included reports

Report or study family	Layer	Design	Evidence domain	Direction	Translation distance	Critical boundary	Synthesis confidence
Estes et al., 2026	A/B	Human RCT	exercise training/substrate oxidation	mixed	direct adult exercise	small male sample; multiplicity; benefits inconsistent at 12 wk	Low–moderate
Park et al., 2024	A/B	Animal	muscle mass/strength/endurance	positive	mouse exercise	shared cohort; no pathway blockade	Moderate
Ko et al., 2024	A/C	Human RCT	weight loss/body composition/aerobic fitness	mixed	direct adult lifestyle program	co-intervention; multiplicity; small BMC decline	Low–moderate
Park et al., 2024	A/B	Animal	lactate/OXPHOS/slow MHC	positive	mouse exercise	shared cohort; fatigue partly biomarker-inferred	Moderate
Harris et al., 2020	A/B	Mixed human/animal	exercise-induced milk 3′-SL/cross-generational metabolism	positive mechanistic	human exposure plus mouse causality	reverse direction vs supplementation; global knockout pleiotropy	Moderate–high animal; low human outcome
Go et al., 2024	A/B	Cell+animal	muscle atrophy/proteolysis	positive	dexamethasone atrophy model	disease model; no direct target blockade	Moderate
Arellano et al., 2023	A/B	C. elegans	endurance/AMPK/adenosine/metabolic shift	positive	invertebrate exercise	major species/dose translation gap	Moderate within model; very low translation
Phan et al., 2026	A/B	Animal	liver–muscle metabolic stress	positive	STZ mouse	n=5/group; prophylactic; no exercise	Low
Kim et al., 2024	A/B	Animal	middle-age muscle/PI3K-AKT/oxidative stress	positive	forced-swim mouse	multiplicity; pathway associations not necessity	Low–moderate
Zhang et al., 2025	A/B	Animal	gut–liver metabolism/energy expenditure	positive	HFD mouse	no monotonic dose response; activity secondary	Low–moderate
Lemoine et al., 2025	A	Human crossover RCT	acute exercise effect on milk 3′-SL/6′-SL	null	direct lactating human exercise exposure	acute design; only two HMOs; no supplementation or downstream outcome	Moderate–high
Zhang et al., 2025	B	Animal	gut–liver multi-omics/activity	positive	HFD mouse	overlapping study family; omics correlations	Low–moderate
Carter et al., 2025	B/C	Human RCT	microbiome/systemic exposure/metabolism	mixed	healthy older adults	primary cytokine endpoint negative; responder/post-hoc analyses	Moderate
Reens et al., 2024	B/C	Gnotobiotic animal	dose→B. infantis→microbial metabolites	positive	adult-human microbiota in mice	few donors; no host performance outcomes	Moderate mechanistic; low

Report or study family	Layer	Design	Evidence domain	Direction	Translation distance	Critical boundary	Synthesis confidence
							translation
Kennedy et al., 2026	B/C	Human crossover RCT	UC symptoms/microbiome/metabolome	mixed	adult disease population	primary clinical endpoint negative; PP biology analysis	Moderate
Canfora et al., 2023	B/C	Human crossover RCT	acute circulating SCFA/substrate metabolism	mixed	adult men	acute; phenotype-dependent; exploratory multiplicity	Low–moderate
Schalich et al., 2024	B	Animal+cell	microbiota/pantothenate/barrier	positive mechanistic	colitis models	rescue does not prove full mediation	Moderate–high mechanistic
Li et al., 2025	B	Animal	ageing gut–T-cell/metabolism	mixed	aged female mouse	skeletal muscle mass null; causal axis untested	Low
Yonekawa et al., 2014	B	Animal+human cells	systemic exposure→muscle sialylation/function	positive mechanistic	GNE myopathy	high dose; disease-specific biology	Moderate–high disease mechanism
Wang et al., 2022	B	Animal	SCFA/GPR43/AMPK-SIRT1/antioxidant	positive associative	d-gal ageing mouse	no blockade; Fisher LSD/multiplicity	Low
Kaur et al., 2025	B	Cell+organoid+animal	ADAM17/HB-EGF/EGFR barrier	positive mechanistic	intestinal models	other targets possible; no muscle/systemic outcomes	Moderate–high mechanistic
Baek et al., 2021	B	Cell	cartilage inflammation/oxidative stress	positive	SW1353 cells	single transformed cell line; exposure relevance unknown	Low
Tran-Duc et al., 2025	B	Animal	systemic anti-inflammatory/metabolic effects	positive	STZ mouse, intraperitoneal	non-oral route; acute prophylaxis; weak muscle inference	Very low for nutrition translation
Chen et al., 2023	B	Cell	AMPK-dependent adipocyte browning	positive mechanistic	adipocyte models	high in-vitro exposure; compound C off-target; no respiration	Moderate within model; low translation
Yu et al., 2024	B	Cell+animal	Nrf2/NF-κB vascular inflammation	positive	LPS model, intraperitoneal	dose-unit inconsistencies; animal n unclear; non-oral	Very low–low
Lee et al., 2020	B	Animal	microbiota/satiety/adiposity	mixed	HFD mouse	effect only at 10% w/v; liver lipids null; no mediation	Low
Jacobs et al., 2023	C	Nonrandom human intervention	complex HMO microbiome/multi-omics	positive exploratory	healthy adults	no placebo; 8/dose; mixture attribution impossible	Low–moderate
Vazquez et al., 2017	C	Animal PK	oral absorption/urinary excretion	positive PK	rat adult/pup	no absolute human bioavailability or tissue exposure	Moderate PK; low adult translation
Jantscher-Krenn et al., 2019	C	Human observational	circulating endogenous HMO	association	pregnancy	endogenous pregnancy physiology, not oral supplementation	Low–moderate for availability
Šuligoj et al., 2020	C	In vitro/ex vivo	microbiota→SCFA→barrier	positive mechanistic	adult microbiota/cells/organoids	no oral clinical confirmation; complex supernatant	Moderate mechanistic
Kim et al., 2022	C	Human RCT	6′-SL safety	neutral/safe	healthy adults	n=60 insufficient for rare events; efficacy not assessed	Moderate safety
Ryan et al., 2021	C	Nonrandom human+ex vivo	IBS/UC symptoms/microbiota/SCFA	positive exploratory	adult GI disease	n=12; open label; composite nutritional formula	Low
Firrman et al., 2024	C	Ex vivo	age-dependent microbiome response	heterogeneous positive	human donor microbiota	n=6/age; ex-vivo prediction	Moderate mechanistic; low clinical
Elison et al., 2016	C	Human RCT	2′-FL/LNnT tolerability/microbiome	positive microbiome/safe	healthy adults	2 wk; multiple arms; surrogate outcomes	Moderate
Vliex et al., 2025	C	Human RCT	post-antibiotic resilience/metabolic health	transient/mixed	overweight/obese adults	2-wk signal absent at 8 wk; most metabolic outcomes null	Moderate
Bajic et al., 2024	C	Ex vivo	low-dose structure-specific metabolites	positive dose response	child/adult donor microbiota	predicted doses; n=6/group; untargeted multiplicity	Low–moderate
Sato et al., 2024	C	Ex vivo+cell	nonbifidogenic SCFA/barrier	positive mechanistic	adult microbiota/Caco-2	n=3 donors; complex supernatant; no clinical test	Low–moderate
Sato et al., 2025	C	Ex vivo	2′-FL/3-FL SCFA timing	positive	3 Japanese adult donors	n=3; no host component	Low–moderate
Lee et al., 2025	C	In vitro/ex vivo	2′-FL+B. infantis synbiotic metabolites	positive mechanistic	adult fecal fermentation	n=3; 24 h; PICRUSt2 predictions; no host outcome	Low
Jackson et al., 2023	C	Human RCT	microbiome/mood with 2′-FL±OF	mixed	working adults with mild symptoms	secondary mood multiplicity; independent 2′-FL effect limited	Moderate

Note. Layer assignment was non-exclusive. Directional coding is descriptive and is not a quantitative quality score or vote-counting estimate.

Supplementary Table S5A. RoB 2 result-level judgements for randomized studies

Study	Result assessed	Variant	D1	D2	D3	D4	D5	Period/carryover	Overall
Estes et al., 2026	Change in aerobic capacity/substrate oxidation	Parallel RoB 2	Some concerns	Low	Low	Low	Some concerns	NA	Some concerns
Ko et al., 2024	Change in body fat percentage/fat-free mass	Parallel RoB 2	Some concerns	Low	Low	Low	Some concerns	NA	Some concerns
Carter et al., 2025	Change in Cytokine Response Score at 6 weeks	Parallel RoB 2	Low	Low	Some concerns	Low	Low	NA	Some concerns
Kennedy et al., 2026	SCCAI clinical response	Crossover RoB 2	Low	Low	Some concerns	Low	Low	Low	Some concerns
Canfora et al., 2023	Postprandial circulating acetate response	Crossover RoB 2	Low	Low	Some concerns	Low	Some concerns	Low	Some concerns
Kim et al., 2022	Serious and gastrointestinal adverse events	Parallel RoB 2	Low	Low	Low	Low	Low	NA	Low
Elison et al., 2016	Gastrointestinal tolerance and Bifidobacterium response	Parallel RoB 2	Some concerns	Low	Low	Low	Low	NA	Some concerns
Vliex et al., 2025	Gut microbial resilience after supplementation	Parallel RoB 2	Low	Low	Low	Low	Low	NA	Low
Jackson et al., 2023	Bifidobacterium count/composition	Parallel RoB 2	Low	Low	Low	Low	Low	NA	Low
Lemoine et al., 2025	Acute post-exercise milk 3′-SL and 6′-SL concentrations	Crossover RoB 2	Low	Low	Low	Low	Low	Low	Low

Note. D1, randomization process; D2, deviations from intended interventions; D3, missing outcome data; D4, measurement of the outcome; D5, selection of the reported result. Four results were Low risk and six had Some concerns; none was High risk.

Supplementary Table S5B. ROBINS-I result-level judgements for non-randomized interventions

Study	Result assessed	D1	D2	D3	D4	D5	D6	D7	Overall
Jacobs et al., 2023	Effect of 7-day HMO concentrate on adult microbiome	Serious	Low	Low	Low	Low	Moderate	Moderate	Serious
Ryan et al., 2021	Effect of 6-week 2′-FL-containing formula on GIQLI	Serious	Moderate	Low	Low	Moderate	Moderate	Moderate	Serious

Note. D1, confounding; D2, selection of participants; D3, classification of interventions; D4, deviations from intended interventions; D5, missing data; D6, measurement of outcomes; D7, selection of the reported result. Serious judgements limit causal interpretation but do not remove descriptive value.

Supplementary Table S5C. JBI-aligned appraisal of the prospective longitudinal exposure study

Study	Design	Result assessed	Strength	Main bias concern	Decision
Jantscher-Krenn et al., 2019	Prospective longitudinal pilot cohort	Prenatal circulating HMO concentrations and associations with body composition	Appropriate for detection/association hypothesis generation	Selection and residual confounding likely; repeated measures and direct HPLC/MS confirmation are strengths	Do not label with ROBINS-I; retain as descriptive systemic-availability evidence

Note. This study was not assessed with ROBINS-I because it was retained as descriptive systemic-availability evidence rather than as an intervention effect estimate.

Supplementary Table S5D1. SYRCLE domain-level judgements for animal studies: domains 1–5

Study	Sequence generation	Baseline characteristics	Allocation concealment	Random housing	Blinding of caregivers
Park et al., 2024	Low	Low	Unclear	Unclear	Unclear
Park et al., 2024	Low	Low	Unclear	Unclear	Unclear
Harris et al., 2020	Low	Unclear	Unclear	Unclear	Unclear
Go et al., 2024	Unclear	Unclear	Unclear	Unclear	Unclear
Phan et al., 2026	Low	Low	Unclear	Unclear	Unclear
Kim et al., 2024	Low	Low	Unclear	Unclear	Unclear
Zhang et al., 2025	Low	Low	Unclear	Unclear	Unclear
Zhang et al., 2025	Low	Low	Unclear	Unclear	Unclear
Reens et al., 2024	Low	Low	Unclear	Unclear	Unclear
Vazquez et al., 2017	Unclear	Unclear	Unclear	Unclear	Unclear
Schalich et al., 2024	Unclear	Unclear	Unclear	Unclear	Unclear
Li et al., 2025	Low	Unclear	Unclear	Unclear	Unclear
Yonekawa et al., 2014	Unclear	Low	Unclear	Unclear	Unclear
Wang et al., 2022	Low	Low	Unclear	Unclear	Unclear
Kaur et al., 2025	Unclear	Unclear	Unclear	Unclear	Unclear
Tran-Duc et al., 2025	Low	Low	Unclear	Unclear	Unclear
Yu et al., 2024	Low	Unclear	Unclear	Unclear	Unclear
Lee et al., 2020	Low	Low	Unclear	Unclear	Unclear

Note. Low, High and Unclear are domain judgements. Unreported procedures were coded Unclear rather than High.

Supplementary Table S5D2. SYRCLE domain-level judgements for animal studies: domains 6–10

Study	Random outcome assessment	Blinding of outcome assessor	Incomplete outcome data	Selective reporting	Other bias	Domain pattern summary
Park et al., 2024	Unclear	Unclear	Low	Unclear	Low/Unclear	No confirmed High-risk domain; predominantly Unclear because reporting is incomplete
Park et al., 2024	Unclear	Unclear	Low	Unclear	Low/Unclear	No confirmed High-risk domain; predominantly Unclear because reporting is incomplete
Harris et al., 2020	Unclear	Unclear	Low	Unclear	Low/Unclear	No confirmed High-risk domain; predominantly Unclear because reporting is incomplete
Go et al., 2024	Unclear	Unclear	Low	Unclear	Low/Unclear	No confirmed High-risk domain; predominantly Unclear because reporting is incomplete
Phan et al., 2026	Unclear	Unclear	Low	Unclear	Low/Unclear	No confirmed High-risk domain; predominantly Unclear because reporting is incomplete
Kim et al., 2024	Unclear	Unclear	Low	Unclear	Low/Unclear	No confirmed High-risk domain; predominantly Unclear because reporting is incomplete
Zhang et al., 2025	Unclear	Unclear	Low	Unclear	Unclear	No confirmed High-risk domain; predominantly Unclear because reporting is incomplete
Zhang et al., 2025	Unclear	Unclear	Low	Unclear	Unclear	No confirmed High-risk domain; predominantly Unclear because reporting is incomplete
Reens et al., 2024	Unclear	Unclear	Low	Unclear	Low/Unclear	No confirmed High-risk domain; predominantly Unclear because reporting is incomplete
Vazquez et al., 2017	Unclear	Unclear	Low	Unclear	Low/Unclear	No confirmed High-risk domain; predominantly Unclear because reporting is incomplete
Schalich et al., 2024	Unclear	Unclear	Low	Unclear	Low/Unclear	No confirmed High-risk domain; predominantly Unclear because reporting is incomplete
Li et al., 2025	Unclear	Unclear	Low	Unclear	Low/Unclear	No confirmed High-risk domain; predominantly Unclear because reporting is incomplete
Yonekawa et al., 2014	Unclear	Unclear	Unclear	Unclear	Low/Unclear	No confirmed High-risk domain; predominantly Unclear because reporting is incomplete
Wang et al., 2022	Unclear	Unclear	Low	Unclear	Low/Unclear	No confirmed High-risk domain; predominantly Unclear because reporting is incomplete
Kaur et al., 2025	Unclear	Low	Low	Unclear	Low/Unclear	No confirmed High-risk domain; predominantly Unclear because reporting is incomplete
Tran-Duc et al., 2025	Unclear	Unclear	Low	Unclear	Low/Unclear	No confirmed High-risk domain; predominantly Unclear because reporting is incomplete
Yu et al., 2024	Unclear	Unclear	Low	Unclear	Unclear	No confirmed High-risk domain; predominantly Unclear because reporting is incomplete
Lee et al., 2020	Unclear	Unclear	Low	Unclear	Low/Unclear	No confirmed High-risk domain; predominantly Unclear because reporting is incomplete

Note. No overall SYRCLE grade was assigned because the tool does not provide a validated algorithm for a single overall rating.

Supplementary Table S5E. Structured appraisal of in vitro and ex vivo mechanism studies

Study	Model relevance	Biological replication	Exposure relevance	Controls	Causal perturbation	Functional endpoint	Multiplicity reporting	Confidence	Main limitation
Šuligoj et al., 2020	High	Moderate	Moderate	Strong	Moderate	Strong	Moderate	Moderate	Complex fermented supernatant and no human intervention.
Firrman et al., 2024	High	Moderate	Moderate	Strong	Weak	Moderate	Moderate	Moderate	Small age strata and ex-vivo prediction.
Bajic et al., 2024	High	Moderate	Low–moderate	Strong	Weak	Moderate	Weak	Low–moderate	Predicted dose mapping and untargeted multiplicity.
Sato et al., 2024	High	Low	Moderate	Adequate	Weak	Moderate	Moderate	Low–moderate	Only three donors; no metabolite-specific mediation.
Sato et al., 2025	High	Low	Moderate	Adequate	Weak	Moderate	Moderate	Low–moderate	Three donors and no host-cell component.
Lee et al., 2025	Moderate	Low	Low	Adequate	Weak	Moderate	Weak	Low	Short fermentation and predicted functional genes.
Baek et al., 2021	Low–moderate	Moderate	Low	Adequate	Weak	Moderate	Moderate	Low	Single transformed chondrocyte-like line.
Chen et al., 2023	Moderate	Moderate	Low	Strong	Moderate	Moderate	Moderate	Moderate	High concentration and pharmacologic inhibitor specificity.
Arellano et al., 2023	Low for humans	Moderate	Low	Strong	Strong genetic	Strong within model	Moderate	Moderate	Invertebrate physiology and dose translation.

Note. Confidence reflects within-model mechanistic support and is interpreted separately from translation to healthy exercising adults.

Supplementary Table S6A. Source data for Figure 2 (PRISMA 2020 flow diagram)

Stage	Source or event	Count	Audit note
Identification	PubMed/MEDLINE	1151	abstract TXT contains 1151 unique PMID

Stage	Source or event	Count	Audit note
Identification	Embase Articles	398	RIS contains 398 records
Identification	Embase Clinical Trials	25	user confirmed on 2026-09-08; separate register stream
Identification	Web of Science batch 1	1000	records 1-1000
Identification	Web of Science batch 2	228	records 1001-1228
Identification	Scopus	1985	RIS contains 1985 records
Identification	SPORTDiscus RIS	25	S3 final result set
Identification	TOTAL BIBLIOGRAPHIC RECORDS	4787	before cross-database deduplication
Identification	TOTAL TRIAL REGISTRATIONS	25	keep separate from bibliographic records
Deduplication	total duplicates removed	2276	2261 high-confidence plus 15 manually adjudicated
Deduplication	final records after deduplication	2511	title-abstract screening baseline
Screening	records screened	2511	final deduplicated screening baseline
Screening	records excluded	2200	1992 proposed exclusions plus 208 insufficient-information records confirmed excluded by user
Retrieval	reports sought for retrieval	311	records retained after title-abstract screening
Retrieval	reports not retrieved	124	311 sought; 187 verified retrieved; 124 not retrieved after public and manual retrieval attempts.
Eligibility	reports assessed for eligibility	187	187 retrieved reports assessed; CQ and SG confirmed 2026-09-11.
Eligibility	reports excluded	147	113 scope/design exclusions plus 34 background/protocol reports; reason breakdown recorded in final screening table.
Included	studies included	38	40 included reports consolidated into study families; two paired-report families identified.
Included	reports included	40	Original evidence reports after CQ and SG full-text confirmation.

Note. Embase Clinical Trials (n = 25) formed a separate registry stream and were not included in the 4,787 bibliographic records.

Supplementary Table S6B. Source data for Figure 3 (direction-of-evidence map)

Study	Evidence group	Model	HMO	Endurance/aerobic	Strength/muscle	Fatigue/recovery	Body composition/metabolism	Mechanistic biomarkers	Safety/tolerability	Scope note
stes et al. [30] Resistance-training CT	Human direct	Recreationally active men	6′-SL	Mixed	Null	Not assessed	Null	Mixed	No major signal	Small male-only proof-of-concept trial; transient signals
o et al. [31] Lifestyle-intervention CT	Human direct	Adults with overweight	2′-FL	Mixed	Not assessed	Not assessed	Mixed	Mixed	Caution	Co-intervention and multiplicity; BMC reduction reported
emoine et al. [34] Acute crossover al	Human reverse-direction	Lactating women	Endogenous 3′-SL/6′-SL	Not assessed	Not assessed	Not assessed	Not assessed	Null	No major signal	Exercise→HMO study; acute milk-HMO response was null
rk et al. [25,26] Mouse study mily	Animal/experimental direct	Male C57BL/6J mice	6′-SL	Positive	Positive	Positive	Not assessed	Positive	No major signal	Two reports from one cohort; pathway necessity no tested
o et al. [27] Dexamethasone rophy model	Animal/experimental direct	Mouse + C2C12	6′-SL	Positive	Positive	Positive	Not assessed	Positive	Not assessed	Disease model; allocation and blinding incompletel reported
rellano Spadaro et al. [28] Worm durance model	Animal/experimental direct	C. elegans	3′-SL/6′-SL	Positive	Not assessed	Positive	Mixed	Positive	Not assessed	Genetic dependence within model; major translatio distance
im et al. [32] Forced-swimming odel	Animal/experimental direct	Middle-aged male mice	2′-FL	Positive	Positive	Mixed	Not assessed	Positive	Not assessed	Multiple doses and outcomes; pathways mostly associative
arris et al. [33] Maternal-exercise ogramme	Animal reverse-direction	Human sampling + mice	3′-SL	Not assessed	Not assessed	Not assessed	Positive	Positive	Not assessed	Exercise→milk HMO→offspring; not athlete supplementation
an et al. [44] Metabolic-stress odel	Animal adjacent	STZ-treated male mice	Sialyllactose	Not assessed	Not assessed	Not assessed	Positive	Positive	Not assessed	n=5/group; no exercise-performance endpoint
ang et al. [45,60] HFD study mily	Animal adjacent	HFD-fed male mice	3′-SL	Contextual	Not assessed	Not assessed	Positive	Positive	Not assessed	Spontaneous activity only; no prescribed exercise

Note. Cells describe reported direction within each model and are not effect sizes. Not assessed indicates that the domain was not evaluated.

Supplementary Table S6C. Source data for Figure 4 (translational evidence ladder)

Context	Evidence stage	Number of studies	Evidence grade	Display label
Adult exercise	Adult feasibility & safety	1	CI	n = 1 CI
Adult exercise	Exercise / training outcomes	3	CI	n = 3 CI
Adult exercise	Microbiome, metabolites & barrier	0	—	0
Adult exercise	Muscle & metabolic mechanisms	1	CI	n = 1 CI
Adult exercise	Systemic exposure	0	—	0
Adult non-exercise	Adult feasibility & safety	9	CI	n = 9 CI
Adult non-exercise	Exercise / training outcomes	0	—	0
Adult non-exercise	Microbiome, metabolites & barrier	7	CI	n = 7 CI
Adult non-exercise	Muscle & metabolic mechanisms	0	—	0
Adult non-exercise	Systemic exposure	2	DM	n = 2 DM
Animal or mixed model	Adult feasibility & safety	0	—	0
Animal or mixed model	Exercise / training outcomes	7	P/R	n = 7 P/R
Animal or mixed model	Microbiome, metabolites & barrier	7	P/R	n = 7 P/R
Animal or mixed model	Muscle & metabolic mechanisms	8	P/R	n = 8 P/R
Animal or mixed model	Systemic exposure	2	P/R	n = 2 P/R
In vitro/ex vivo	Adult feasibility & safety	0	—	0
In vitro/ex vivo	Exercise / training outcomes	0	—	0
In vitro/ex vivo	Microbiome, metabolites & barrier	5	P/R	n = 5 P/R
In vitro/ex vivo	Muscle & metabolic mechanisms	1	P/R	n = 1 P/R
In vitro/ex vivo	Systemic exposure	0	—	0

Note. Grades are the calibrated categories used in the figure: I, internal mechanistic evidence; P, preclinical evidence; CI, clinical indirect evidence; CD, clinical direct evidence. Counts reflect independent studies, not reports.

Figures 1 and 5 are conceptual synthesis figures rather than plots derived from a numerical dataset. Their evidence basis is documented in Supplementary Tables S3–S5 and in the manuscript text; dashed links identify unverified causal relations.

Supplementary File S1. Full electronic search strategies

The following syntax reproduces the database-specific strategies used for the structured search. No date, language, publication-type, human/animal or age limits were applied. The Embase strategy is the executed FINAL v2.2 version; automatic mapping options were disabled because controlled terms and fields were specified explicitly.

PubMed/MEDLINE (records exported: 1,151)

```
(
  "human milk oligosaccharide"[tiab]
  OR "human milk oligosaccharides"[tiab]
  OR ("Oligosaccharides"[Mesh] AND "Milk, Human"[Mesh])
  OR fucosyllactos*[tiab]
  OR sialyllactos*[tiab]
  OR "lacto-N-neotetraose"[tiab]
  OR "lacto N neotetraose"[tiab]
  OR LNnT[tiab]
  OR "lacto-N-tetraose"[tiab]
  OR "lacto N tetraose"[tiab]
  OR difucosyllactos*[tiab]
  OR "difucosyl lactose"[tiab]
)
AND
(
  "Exercise"[Mesh]
  OR "Exercise Training"[tiab]
  OR "Resistance Training"[Mesh]
  OR exercis*[tiab]
  OR train*[tiab]
  OR "physical activ*"[tiab]
  OR "physical performance"[tiab]
  OR "exercise performance"[tiab]
  OR endurance[tiab]
  OR fatigue[tiab]
  OR "fatigue resistance"[tiab]
  OR "Muscle, Skeletal"[Mesh]
  OR "skeletal muscle"[tiab]
  OR "skeletal muscles"[tiab]
  OR myotube*[tiab]
  OR myocyte*[tiab]
  OR "muscle mass"[tiab]
  OR "muscle strength"[tiab]
  OR "muscle function"[tiab]
  OR hypertroph*[tiab]
  OR atroph*[tiab]
  OR "Body Composition"[Mesh]
  OR "body composition"[tiab]
  OR "fat-free mass"[tiab]
  OR "lean mass"[tiab]
  OR "Energy Metabolism"[Mesh]
  OR metabol*[tiab]
  OR mitochondri*[tiab]
  OR "oxidative phosphorylation"[tiab]
  OR OXPHOS[tiab]
  OR AMPK[tiab]
  OR "PI3K"[tiab]
  OR AKT[tiab]
  OR "substrate oxidation"[tiab]
  OR "fat oxidation"[tiab]
  OR "lipid oxidation"[tiab]
  OR "substrate utilization"[tiab]
  OR "metabolic flexibility"[tiab]
  OR "glucose metabolism"[tiab]
  OR "lipid metabolism"[tiab]
  OR glycolipid[tiab]
  OR "energy expenditure"[tiab]
  OR "oxidative stress"[tiab]
  OR lactate[tiab]
  OR "gut-muscle"[tiab]
  OR "gut muscle"[tiab]
  OR (
      (
        "Adult"[Mesh]
        OR "Young Adult"[Mesh]
        OR "Middle Aged"[Mesh]
        OR "Aged"[Mesh]
        OR adult*[tiab]
        OR "middle aged"[tiab]
        OR older[tiab]
        OR elderly[tiab]
        OR volunteer*[tiab]
      )
      AND
      (
        "Gastrointestinal Microbiome"[Mesh]
        OR microbiom*[tiab]
        OR microbiota[tiab]
        OR "short-chain fatty acid"[tiab]
        OR "short-chain fatty acids"[tiab]
        OR SCFA*[tiab]
        OR "intestinal barrier"[tiab]
        OR "gut barrier"[tiab]
        OR "gut permeability"[tiab]
        OR inflamm*[tiab]
        OR immun*[tiab]
        OR "systemic availability"[tiab]
        OR bioavailab*[tiab]
        OR absorp*[tiab]
        OR pharmacokinetic*[tiab]
        OR plasma[tiab]
        OR serum[tiab]
        OR urine[tiab]
        OR tolerab*[tiab]
        OR safety[tiab]
      )
  )
)
```

Embase.com FINAL v2.2 (records exported: 398 articles; 25 trial registrations in the separate registry stream)

```
(
  'human milk oligosaccharide*':ti,ab,kw OR fucosyllactos*:ti,ab,kw OR sialyllactos*:ti,ab,kw
  OR 'lacto-N-neotetraose':ti,ab,kw OR 'lacto N neotetraose':ti,ab,kw OR LNnT:ti,ab,kw
  OR 'lacto-N-tetraose':ti,ab,kw OR 'lacto N tetraose':ti,ab,kw
  OR difucosyllactos*:ti,ab,kw OR 'difucosyl lactose':ti,ab,kw
)
AND
(
  'exercise'/exp OR 'physical activity'/exp OR 'skeletal muscle'/exp OR 'body composition'/exp
  OR exercis*:ti,ab,kw OR 'exercise training':ti,ab,kw OR 'resistance training':ti,ab,kw
  OR 'strength training':ti,ab,kw OR 'endurance training':ti,ab,kw OR 'aerobic training':ti,ab,kw
  OR 'physical activ*':ti,ab,kw OR 'physical performance':ti,ab,kw OR 'exercise performance':ti,ab,kw
  OR endurance:ti,ab,kw OR fatigue:ti,ab,kw OR 'fatigue resistance':ti,ab,kw
  OR 'skeletal muscle*':ti,ab,kw OR myotube*:ti,ab,kw OR myocyte*:ti,ab,kw
  OR 'muscle mass':ti,ab,kw OR 'muscle strength':ti,ab,kw OR 'muscle function':ti,ab,kw
  OR hypertroph*:ti,ab,kw OR atroph*:ti,ab,kw OR 'body composition':ti,ab,kw
  OR 'fat-free mass':ti,ab,kw OR 'lean mass':ti,ab,kw OR mitochondri*:ti,ab,kw
  OR 'oxidative phosphorylation':ti,ab,kw OR OXPHOS:ti,ab,kw OR AMPK:ti,ab,kw
```

```
  OR PI3K:ti,ab,kw OR AKT:ti,ab,kw OR 'substrate oxidation':ti,ab,kw
  OR 'fat oxidation':ti,ab,kw OR 'lipid oxidation':ti,ab,kw OR 'substrate utilization':ti,ab,kw
  OR 'metabolic flexibility':ti,ab,kw OR 'energy expenditure':ti,ab,kw
  OR 'oxidative stress':ti,ab,kw OR lactate:ti,ab,kw OR 'gut-muscle':ti,ab,kw OR 'gut muscle':ti,ab,kw
  OR (
    ('adult'/exp OR adult*:ti,ab,kw OR 'young adult*':ti,ab,kw OR 'middle aged':ti,ab,kw
     OR older:ti,ab,kw OR elderly:ti,ab,kw OR volunteer*:ti,ab,kw)
    AND
    (microbiom*:ti,ab,kw OR microbiota:ti,ab,kw OR 'short-chain fatty acid*':ti,ab,kw
     OR SCFA*:ti,ab,kw OR 'intestinal barrier':ti,ab,kw OR 'gut barrier':ti,ab,kw
     OR 'gut permeability':ti,ab,kw OR 'systemic availability':ti,ab,kw
     OR bioavailab*:ti,ab,kw OR absorp*:ti,ab,kw OR pharmacokinetic*:ti,ab,kw)
  )
)
```

Web of Science Core Collection (records exported: 1,228)

```
TS=(
  "human milk oligosaccharide*"
  OR fucosyllactos*
  OR sialyllactos*
  OR "lacto-N-neotetraose"
  OR "lacto N neotetraose"
  OR LNnT
  OR "lacto-N-tetraose"
  OR "lacto N tetraose"
  OR difucosyllactos*
  OR "difucosyl lactose"
)
AND
TS=(
  exercis*
  OR train*
  OR "physical activ*"
  OR "physical performance"
  OR "exercise performance"
  OR endurance
  OR fatigue
  OR "fatigue resistance"
  OR "skeletal muscle*"
  OR myotube*
  OR myocyte*
  OR "muscle mass"
  OR "muscle strength"
  OR "muscle function"
  OR hypertroph*
  OR atroph*
  OR "body composition"
  OR "fat-free mass"
  OR "lean mass"
  OR metabol*
  OR mitochondri*
  OR "oxidative phosphorylation"
  OR OXPHOS
  OR AMPK
  OR PI3K
  OR AKT
  OR "substrate oxidation"
  OR "fat oxidation"
  OR "lipid oxidation"
  OR "substrate utilization"
  OR "metabolic flexibility"
  OR "glucose metabolism"
  OR "lipid metabolism"
  OR glycolipid
  OR "energy expenditure"
  OR "oxidative stress"
  OR lactate
  OR "gut-muscle"
  OR "gut muscle"
  OR (
      (adult* OR "young adult*" OR "middle aged" OR older OR elderly OR volunteer*)
      AND
      (
        microbiom*
        OR microbiota
        OR "short-chain fatty acid*"
        OR SCFA*
        OR "intestinal barrier"
        OR "gut barrier"
        OR "gut permeability"
        OR inflamm*
        OR immun*
        OR "systemic availability"
        OR bioavailab*
        OR absorp*
        OR pharmacokinetic*
        OR plasma
        OR serum
        OR urine
        OR tolerab*
        OR safety
      )
  )
)
```

Scopus (records exported: 1,985)

```
TITLE-ABS-KEY (
  "human milk oligosaccharide*"
  OR fucosyllactos*
  OR sialyllactos*
  OR "lacto-N-neotetraose"
  OR "lacto N neotetraose"
  OR LNnT
  OR "lacto-N-tetraose"
  OR "lacto N tetraose"
  OR difucosyllactos*
  OR "difucosyl lactose"
)
AND
TITLE-ABS-KEY (
  exercis*
  OR train*
  OR "physical activ*"
  OR "physical performance"
  OR "exercise performance"
  OR endurance
  OR fatigue
  OR "fatigue resistance"
  OR "skeletal muscle*"
  OR myotube*
```

```
  OR myocyte*
  OR "muscle mass"
  OR "muscle strength"
  OR "muscle function"
  OR hypertroph*
  OR atroph*
  OR "body composition"
  OR "fat-free mass"
  OR "lean mass"
  OR metabol*
  OR mitochondri*
  OR "oxidative phosphorylation"
  OR OXPHOS
  OR AMPK
  OR PI3K
  OR AKT
  OR "substrate oxidation"
  OR "fat oxidation"
  OR "lipid oxidation"
  OR "substrate utilization"
  OR "metabolic flexibility"
  OR "glucose metabolism"
  OR "lipid metabolism"
  OR glycolipid
  OR "energy expenditure"
  OR "oxidative stress"
  OR lactate
  OR "gut-muscle"
  OR "gut muscle"
  OR (
      (adult* OR "young adult*" OR "middle aged" OR older OR elderly OR volunteer*)
      AND
      (
        microbiom*
        OR microbiota
        OR "short-chain fatty acid*"
        OR SCFA*
        OR "intestinal barrier"
        OR "gut barrier"
        OR "gut permeability"
        OR inflamm*
        OR immun*
        OR "systemic availability"
        OR bioavailab*
        OR absorp*
        OR pharmacokinetic*
        OR plasma
        OR serum
        OR urine
        OR tolerab*
        OR safety
      )
  )
)
```

SPORTDiscus (EBSCOhost) (records exported: 25)

```
S1 =
TI (
  "human milk oligosaccharide*"
  OR fucosyllactos*
  OR sialyllactos*
  OR "lacto-N-neotetraose"
  OR "lacto N neotetraose"
  OR LNnT
  OR "lacto-N-tetraose"
  OR "lacto N tetraose"
  OR difucosyllactos*
  OR "difucosyl lactose"
)
OR AB (
  "human milk oligosaccharide*"
  OR fucosyllactos*
  OR sialyllactos*
  OR "lacto-N-neotetraose"
  OR "lacto N neotetraose"
  OR LNnT
  OR "lacto-N-tetraose"
  OR "lacto N tetraose"
  OR difucosyllactos*
  OR "difucosyl lactose"
)
OR SU (
  "human milk oligosaccharide*"
  OR fucosyllactos*
  OR sialyllactos*
  OR "lacto-N-neotetraose"
  OR "lacto N neotetraose"
  OR LNnT
  OR "lacto-N-tetraose"
  OR "lacto N tetraose"
  OR difucosyllactos*
  OR "difucosyl lactose"
)

S2 =
TI (
  exercis*
  OR train*
  OR "physical activ*"
  OR "physical performance"
  OR "exercise performance"
  OR endurance
  OR fatigue
  OR "fatigue resistance"
  OR "skeletal muscle*"
  OR myotube*
  OR myocyte*
  OR "muscle mass"
  OR "muscle strength"
  OR "muscle function"
  OR hypertroph*
  OR atroph*
  OR "body composition"
  OR "fat-free mass"
  OR "lean mass"
  OR metabol*
  OR mitochondri*
  OR "oxidative phosphorylation"
  OR OXPHOS
  OR AMPK
  OR PI3K
```

```
  OR AKT
  OR "substrate oxidation"
  OR "fat oxidation"
  OR "lipid oxidation"
  OR "substrate utilization"
  OR "metabolic flexibility"
  OR "glucose metabolism"
  OR "lipid metabolism"
  OR glycolipid
  OR "energy expenditure"
  OR "oxidative stress"
  OR lactate
  OR "gut-muscle"
  OR "gut muscle"
  OR (
      (adult* OR "young adult*" OR "middle aged" OR older OR elderly OR volunteer*)
      AND
      (
        microbiom*
        OR microbiota
        OR "short-chain fatty acid*"
        OR SCFA*
        OR "intestinal barrier"
        OR "gut barrier"
        OR "gut permeability"
        OR inflamm*
        OR immun*
        OR "systemic availability"
        OR bioavailab*
        OR absorp*
        OR pharmacokinetic*
        OR plasma
        OR serum
        OR urine
        OR tolerab*
        OR safety
      )
  )
)
OR AB (
  exercis*
  OR train*
  OR "physical activ*"
  OR "physical performance"
  OR "exercise performance"
  OR endurance
  OR fatigue
  OR "fatigue resistance"
  OR "skeletal muscle*"
  OR myotube*
  OR myocyte*
  OR "muscle mass"
  OR "muscle strength"
  OR "muscle function"
  OR hypertroph*
  OR atroph*
  OR "body composition"
  OR "fat-free mass"
  OR "lean mass"
  OR metabol*
  OR mitochondri*
  OR "oxidative phosphorylation"
  OR OXPHOS
  OR AMPK
  OR PI3K
  OR AKT
  OR "substrate oxidation"
  OR "fat oxidation"
  OR "lipid oxidation"
  OR "substrate utilization"
  OR "metabolic flexibility"
  OR "glucose metabolism"
  OR "lipid metabolism"
  OR glycolipid
  OR "energy expenditure"
  OR "oxidative stress"
  OR lactate
  OR "gut-muscle"
  OR "gut muscle"
  OR (
      (adult* OR "young adult*" OR "middle aged" OR older OR elderly OR volunteer*)
      AND
      (
        microbiom*
        OR microbiota
        OR "short-chain fatty acid*"
        OR SCFA*
        OR "intestinal barrier"
        OR "gut barrier"
        OR "gut permeability"
        OR inflamm*
        OR immun*
        OR "systemic availability"
        OR bioavailab*
        OR absorp*
        OR pharmacokinetic*
        OR plasma
        OR serum
        OR urine
        OR tolerab*
        OR safety
      )
  )
)
OR SU (
  exercis*
  OR train*
  OR "physical activ*"
  OR "physical performance"
  OR "exercise performance"
  OR endurance
  OR fatigue
  OR "fatigue resistance"
  OR "skeletal muscle*"
  OR myotube*
  OR myocyte*
  OR "muscle mass"
  OR "muscle strength"
  OR "muscle function"
  OR hypertroph*
  OR atroph*
  OR "body composition"
```

```
  OR "fat-free mass"
  OR "lean mass"
  OR metabol*
  OR mitochondri*
  OR "oxidative phosphorylation"
  OR OXPHOS
  OR AMPK
  OR PI3K
  OR AKT
  OR "substrate oxidation"
  OR "fat oxidation"
  OR "lipid oxidation"
  OR "substrate utilization"
  OR "metabolic flexibility"
  OR "glucose metabolism"
  OR "lipid metabolism"
  OR glycolipid
  OR "energy expenditure"
  OR "oxidative stress"
  OR lactate
  OR "gut-muscle"
  OR "gut muscle"
  OR (
      (adult* OR "young adult*" OR "middle aged" OR older OR elderly OR volunteer*)
      AND
      (
        microbiom*
        OR microbiota
        OR "short-chain fatty acid*"
        OR SCFA*
        OR "intestinal barrier"
        OR "gut barrier"
        OR "gut permeability"
        OR inflamm*
        OR immun*
        OR "systemic availability"
        OR bioavailab*
        OR absorp*
        OR pharmacokinetic*
        OR plasma
        OR serum
        OR urine
        OR tolerab*
        OR safety
      )
  )
)

S3 = S1 AND S2
```
